# The Essays, Cases, Intelligence, &c.: F—G

**Published:** 1820

**Authors:** 


FABÆ PICHURIM, its efficacy in the fluor albus, vi. 396; analysis of its constituent particles, 377; its varieties, ibid.; oil extracted from it, 378; how to be used, ibid.

FABAS, M. his account of the mineral springs in the higher Pyrenees, i. 414.

FABER, Tanaquil, account of his curious work, entitled Strychnomania, xxv. 385.

FABRE, M. his case of polypus in the stomach rejected by vomiting, xxxiv. 254.

FABRICIUS, (Hieronymus,) an anatomist of the 16th century, an account of his works, ii. 68; his observation on the character of Galen, 165; particulars of his discoveries in anatomy, 166, 168; his inaccuracy in regard to the functions of the pulmonary vessels, 371; his description of the arterial canal, 372; and of the venas ductus, 373; he corrected an error respecting the course of the aorta, 459; his account of the omentum, iii. 73; on the human cæcum, 75; his observations on the uterus, 160; on the liquor amnii, ibid.; denied the existence of the allantois, 161; on the optic nerves and their communication with the brain, iii. 357; on a supposed cavity in the same, 358.

FABRICIUS, J. C. review of his entomological work, entitled Illustratio Iconographica Insectorum, iv. 450.

FABRONI, M. his attempt to prove that alcohol is not essential to the formation of ether, i. 301; his letter on galvanic electricity, x. 54.

FACE, erysipelas in it cured by the application of hot flour, i. 153; on the complaint named the Fothergillian face-ache, ii. 465; case of that complaint successfully treated, 466; observations on the facial nerve, iii. 362; observations on the dolor facei, or tic douloureux, 575; beneficially treated with datura stramonium, ibid.; on the presentation of the face towards the os pubis, iv. 163; how to remedy in that case, 164; the erysipelas of the face contagious, 301; remarks on the history and nature of the disease termed dolor facei, vi. 158; proposal for altering its name to prosopalgy, 159; enumeration of writers on that subject, ibid.; generally attacks women, ibid.; its cessation during pregnancy, 161; and also in intermittents, ibid.; belongs to the class of convulsive disorders, 162; its symptoms described, ibid.; seat of the pain, 163; remote and internal causes, ibid.; its exanthematous acrimony, 243; variety of appearances observed in that complaint, ibid.; cases of it, 244, 246; a remarkable disorder in the face relieved by galvanism, viii. 527; union by the first intention necessary in wounds of the face, ix. 167; singular case of it in a man, x. 420; cured by incision, 421; peculiarly affected by psoriasis diffusa, xii. 97; swellings in the face produced by the common night-shade, 136; on the Fothergillian face-ache, xiii. 367; benefit of sal ammoniac and vinegar in pains of the face, xiii. 510; extraordinary ulcerations of the face, which returned after being apparently cured, xiv. 116; benefit of the arsenical solution in that case, 117; its singular appearance in a disorder peculiar to miners, xv. 93; xvii. 474; case of dolor facei, where the dissection of the nerve had no effect, 230; case of successful operation in the same complaint, xvi. 178; disorders in the face hereditary, xviii. 135; tic doloureux cured with cicuta, 136; cured by mercurial salivation, 176; Fowler's mineral solution beneficial in the same complaint, 252; case of tic douloureux cured by calomel and opium, xx. 180; eruptions on the face in a case of leucorrhœa, xxi. 40; benéfit of cantharides in that complaint, 41; history of an unsuccessful case of dolor facei, xxv. 167; how to treat favus when it attacks the face, 498; case of deformity of the face and throat from burning, removed by surgical operation, xxvi. 158; case of tic douloureux cured by arsenic, 494; observations on the physiognomic appearances peculiar to certain diseases, xxvii. 38; on the portraits of hypochondriacs, 40; on those of epileptics, 41; case of the efficacy of arsenical solution in a disorder of the face, 281; on the varieties of the human countenance in different climates, xxviii. 489; brief account of the notices taken of the tic douloureux, xxxi. 179; description of that disease, 181; both sides of the face seldom affected, 182; how excited, ibid.; relieved by amusement, 183; symptoms which distinguish neuralgia from trismus, ibid.; symptoms distinguishing neuralgia of the face from odontalgia, 184; symptoms that distinguish neuralgia and rheumatism of the face, 186; symptoms distinguishing neuralgia from hemicrania, 187; cases in which the disorder was removed by pills composed of equal parts of hyoscyamus, valerian, and oxide of zinc, 380, 381; benefit of conium maculatum in that complaint, 383; observations on that remedy, 384; advice respecting it, 385; on the operation of dividing diseased nerves, ibid.; various stimulants efficacious in the complaint, 386; cured by mercurial salivation, 387; benefit of aqua ammoniæ in that disorder, ibid.; cases of it, 390; case of paralysis of the face, 497; example of symptoms resembling tic douloureux, 499; case of eruptions on the face, xxxii. 165; case of tic douloureux cured by the external application of tar, 219; case of tic douloureux which seemed to be relieved by belladonna after an ineffectual operation, 433.

FÆCES, whether they remain indurated in the bowels after bilious diarrhœa, vi. 492; want of retentive power over the, xiii. 129; on those of fowls, xxix. 508; ambergris in the whale supposed to be fæces, xxxi. 45; olive oil concreted into fæces, 46.

FACIOLA HEPATICA, an insect found on vegetables, supposed to generate the rot in sheep, xi. 440.

FACTS, advice in regard to the reporting of medical cases as facts on imperfect evidence, xxvii. 115.

FAGUS, *(Castanea,)* or the chesnut, botanical description of it, xvi. 73; starch made from the nut, 74; uses of the wood, ibid. *Fagus Sylvatica,* or the beech-tree described, 74; giddiness caused by eating the nuts, ibid.; oil made into butter from it, ibid.; uses of the leaves and wood, ibid.

FAIRFIELD, in North America, list of professors and students in the Medical Institution at, xxviii. 79.

FAIRLESS, Mr. his description of the life-boat built at Shields, iii. 286.

FAIRY RINGS, not caused by electricity, but fungi, xvii. 197.

FAITHORN, John, on a supposed failure of vaccine inoculation, xii. 360; notice of his Facts and Observations on the nature and treatment of Liver Complaints, xxxii. 170.

FAIVRE, G. on the effect of opium and mercury externally applied by friction in venereal complaints, iii. 168.

FALCHENBERGIUS, his observations on the effects of smoking tobacco on the brain, xxv. 139.

FALCONER, Dr. William, his observations on the pulse translated into German, i. 89; his enquiry into the composition and effects of the Portland powder as a remedy in gout, v. 417; account of his treatise on the Bath waters, vi. 414; on the cure of typhus by bathing, xii. 249; on the similarity of ancient and modern opinions respecting the morbus cardiacus, xv. 377; on the use of the Bath waters in disorders of the hip-joint, 565; on the indiscriminate use of mercurial preparations, xxv. 61; on the bad effects of calomel in hepatitis, 62.

FALLOPIAN TUBES, the manner in which they convey the semen to the ovaria, ii. 246; the media of communication from the uterus to the ovaria, 323; historical account of their discovery, iii. 159; extraordinary instance of encysted dropsy of the, xi. 73; confounded with extra-uterine pregnancy, 75; observations on such cases, 78; their offices in generation, xvii. 493; their uses in generation, 498; time taken up in the passing of the semen through them to the ovarium, xxiii. 280; their appearance in a case of sudden death, xxvii. 180; on secretions in them, xxxii. 162.

FALLOPIUS, Gabriel, an illustrious anatomist of the sixteenth century, his observations on gun-shot wounds, ii. 57; professional character of him, 67; his accurate view of the anatomy of the ear, 160; his description of the ventricles of the brain, 161; on the cartilages of the sternum, 162; on the composition of a muscle, 163; his correction of errors respecting the muscles, 164; observations on the muscles of the tongue, 165; his excellence as a practical operator, 280; his exact description of the arterial canal, 371; his account of the foramen ovale, 372; on the anastomosis between the carotids and vertebral arteries, 460; on the blood vessels of the brain, ibid.; corrects an error of Vesalius on the external jugular veins, 461; on the anastomosis of the vena sine pari, 462; his discovery of the lymphatics, 463; on the origin of ruptures in females, iii. 73; his observations on the panacas, 74; on the human cœcum, 75; his discovery of the tubes in the kidneys, 77; his remark on the uvula, 78; describes the course of the cornea lachrymelia, 79; his account of the ciliary processes, ibid.; the vesiculæ seminales discovered by him, 157; he first described the clitoris, ibid.; on the hymen, 158; on the muscular fibres of the uterus, ibid.; his discovery of the tubes which go by his name, 159; acquainted with the corpora lutea, ibid.; his opinion on the urine of the fœtus, 161; he demonstrated that the optic nerve was covered by the dura mater, 260; on the impropriety of dividing the nerves into pairs, ibid.; observations by him on the optic nerves, 357; his correction of an error in respect to the muscles of the eye, 358; on the pathetic nerve, 359; the accuracy of his observations on the nerves, 362; on the nerve of the voice, iv. 340; importance of his discoveries, 343; his description of the sarcocele of Egypt, xxv. 289.

FAMILIES, review of medical admonitions addressed to, i. 310; announcement of a new edition of that work, 403; advice to them on the treatment of diseases and accidents, 491; cautions to parents on the management of their children, ii. 394; practical observations on the physical treatment of children, v. 293; review of the Family Physician or Domestic Medical Friend, by Alex. Thomson, M.D. vi. 176; account of a practical treatise on Diet for their use, 178; on the children of the poor, x. 573; caution to families in regard to children's disorders, xi. 454; remonstrance to women on their treatment of infants, 467; a family poisoned by the evolution of septic gas, xvii. 225; dangerous effects of meddling with family medicine, 291; evil effects of empiricism in children, ibid.; why families seldom hold estates long, xviii. 181; remarkable history of an hæmorrhagic disposition in one, xx. 69; review of a new medical compendium for, xxi. 167; considerations on the nature and treatment of hereditary diseases, 229, 281; some families distinguished by large heads, 233; particular conformations in certain families, 235; external deformity descendant in many cases, ibid.; hereditary affections of the nervous system, 237; scrofula a common taint, 283; on the hereditary nature of gout, 285; marriage corrects family diseases, 296; advice to families on the subject of various accidents, xxix. 492; on the hereditary properties of diseases, xxxii. 140; account of a remarkable disposition to hæmorrhage in the individuals of the same family, xxxiii. 9.

FANATICISM, remarkable instance of religious, iv. 15; farther observations on the same, 106; extraordinary history of madness produced by it in a whole family, viii. 311; extraordinary case of voluntary abstinence on a religious account, xv. 510; remarkable effects of fanaticism in several towns in Cornwall, xxxi. 373; observations on that account, by way of reply, 464; remarks in answer to the objections and statement advanced on that case, xxxii. 32; on the case of Joanna Southcott, 310; anecdotes of that extraordinary imposture, xxxiii. 138.

FARCY, utility of cantharides in that complaint, xviii. 557; horses inoculated with the matter of farcy, xxv. 281.

FARDEAU, M. his observations on the strangulated scrotal hernia, ix. 260.

FARINA, that of plants a combination of carbon, hydrogen, and oxygen, i. 172; on the soluble and digestive quality of farinaceous seeds, xxii. 403.

FARISH, Thomas, his remarkable case of calculus, xvii. 64.

FARMING, its connexion with physical science, i. 45; account of an extraordinary crop of corn, 47; questions on the improvement of soils by vegetation, xxvi. 82; extraordinary production of oats, xxix. 84; application of chemistry to agriculture, xxx. 13.

FARMS, the cow-pox breaks out in several near London, i. 11.

FARO ISLANDS, remedy for the scurvy used by the inhabitants of the, xv. 70.

FARQUHAR, Sir Walter, attempt to refute his opinions on the benefit of ice in burns, iv. 454; his evidence in favour of the vaccine inoculation before a Committee of the House of Commons, vii. 492; letter addressed to him on the treatment of cancerous diseases, viii. 295; letter to him on the subject of a particular complaint in the bowels, ix. 183; letter to him from Ceylon on the progress of vaccination in the East, 457; on infectious fevers in London, xii. 161.

FARQUHARSON, Dr. William, his testimony in favour of vaccination, xii. 13; his account of the Vaccine Institution at Edinburgh, xiii. 286; his letter on medical reform, xvii. 195; his report on vaccination in Scotland, xviii. 110.

FARR, Dr. recommendation of his treatise on Medical Jurisprudence, vii. 283; review of his Elements of Medical Jurisprudence, xxxii. 326.

FARRE, Dr. J. R. review of his edition of Mr. Saunders's posthumous work on the diseases of the eye, xxvii. 229, 335; xxviii. 20; his remonstrance to Mr. Stevenson on his obligations to, and treatment of Mr. Saunders, xxviii. 467; review of his treatise on the Morbid Anatomy of the Liver, part the first, xxix. 152; reply to him by Mr. Stevenson, 199; character of his work on the morbid alterations of the structure of the liver, xxx. 8; his cases of cynanche laryngea, 165; appendix to that paper, descriptive of the symptoms, 236; his pathological researches on malformations of the heart, xxxii. 513; xxxiii. 49; account of his second fasciculus on the Morbid Anatomy of the Liver, xxxiv. 72; his case of diffused lymph in the cavities of the pleura and pericardium, 132; case of aneurism of the aorta, 146.

FARRELL, Dr. Charles, his observations on ophthalmia and its consequences, xxv. 522.

FARRIERS, observations on the general ignorance of, xviii. 555.

FASHION, that there is one in philosophy as well as in other pursuits, xiv. 387, *note*; injurious effects of it upon health, xv. 91; its influence in medical practice, xvii. 267; xxviii. 45.

FASTING, its benefit in several diseases, i. 43; advantageous in fever, 44; extraordinary history of an imposture in pretended abstinence at Osnabruck, iv. 86; pernicious effects of rigid abstinence on a religious account, 147; detection of an imposture in regard to excessive abstinence, 187; detection of an impostor who pretended to live without food, vii. 194; case of voluntary abstinence on a religious account carried to fatal excess, xv. 510; account of the case of Anne Moore, of Tutbury, xx. 402; farther particulars of that case, with pathological remarks, 527; additions to that statement, xxi. 61; extraordinary case of Mary Thomas, 96; particulars of the abstinence of Anne Moore, of Tutbury, by Benjamin Granger, xxii. 337; suspicions of imposture in that case, xxiv. 309; observations on extraordinary instances recorded by various writers, 312; publication of portraits of two women remarkable for their long abstinence, xxv. 85; necessity of it in wounds of the intestines, xxvii. 61; observations proving the imposture of Anne Moore, xxix. 109; announcement of an account, with portraits of Mary Thomas and Anne Moore, 349; examination of the imposture of Anne Moore, 408; compared with Anna Kinker, 409; farther particulars of the detection of the imposture of Anne Moore, 469; observations on that circumstance, xxx. 21; remarks on extraordinary histories of fasting of long duration, 103; generally confined to the female sex, 107; farther observations on the case of Anne Moore, 109; enlarged account of Mary Thomas of Merionethshire, who existed many years without food; and of Anne Moore, commonly called the fasting woman of Tutbury, 157; memorial addressed by Dr. Henderson to the committee on the case of Anne Moore, 187; remarks on that case, 189; extraordinary cases of, 338; explanation in regard to that imposture, 377; account of the second watching of her, 516; a case of extraordinary abstinence considered as miraculous, xxxi. 50.

FAT, that of pork and mutton detrimental, i. 51; the basis of every vegetable oil, 76; combination of carbon and hydrogen in that of animals, 169; rancid fat serviceable in the composition of mercurial ointment, 357; easy method of combining quicksilver with fat, ii. 389; remarkable case in which part of a female body was converted into fatty matter, iii. 10; nutritious quality of that of mammiferous animals, 66; nature of that of the sea-turtle, 68; one of the principal remedies in poison, vi. 70; quality of the perfumed fat of different animals, 343; resemblance of the ear-wax to the fat of the gall, 475; on the peculiar quality of the fat of cancerous breasts, vii. 163; distillation of fat, ix. 78; extraordinary quantity in the intestines of a man who died of diabetes, xiv. 269; the parts of the animal body changed into fatty matter after death, xvi. 302; the accumulation of it an occasional cause of pulmonary consumption, xix. 339; remarks on corpulence as a disease, xxv. 449; cases of extraordinary obesity, 450; on that of the intestines, xxix. 507; chemical examination of the fatty matter of the brain, xxx. 58, 60; on the formation of fat in the intestines of living animals, xxxi. 43; instance of a female corpse converted into fatty matter, 44; solid masses of fat found in the human colon, 45; fat formed in the intestines and voided with the fæces, 46; experiments on the conversion of animal matter into fat, 47; formed by the means of bile, 49; necessary for the growth of animals, 50; account of experiments on the nature of fat in animal bodies, xxxiv. 435.

FAUBERGE, Dr. on the pernicious effects of mercury in scrofulous cases, xxii. 494.

FAUCES, emetic tartar injected into a vein to expel an obstruction of the, xi. 190; on relieving spasms of the, 545.

FAULKNER, Dr. A. B. on the degree of exercise necessary in dyspepsia, xv. 273; review of his Considerations on the expediency of an hospital for officers on foreign service, xxv. 348.

FAUST, Dr. his plan for the extirpation of the small-pox, i. 83; recommends surgical instruments to be dipped in oil, xiv. 480.

FAVUS, prevalence of that kind of herpetic eruption, xiv. 198; description of the complaint, 199; efficacy of the unguentum picis liquidæ in it, xxv. 498; description of the pustules of that genus, xxx. 415.

FAWELL, Thomas, his remarkable case of superfœtation, xxii. 48; observations on that case, 293.

FAWSSETT, Dr. his treatment of fractured skull, iii. 25; his observations and cases on the dangerous effects of inoculating with spurious vaccine matter, vi. 117.

FAYERMAN, Arnall Thomas, his successful case of hydrothorax, xxix. 369; benefit of blisters and mercurial frictions in that case, 370; remarks on the case, 373.

FEAR, its debilitating influence in febrile diseases, i. 202; hydrophobia produced by it, xvi. 438; calculated to produce atony, xviii. 465; its extraordinary effect in disposing the body to receive the infection of the plague, xxii. 199.

FEARON, Henry, his observations on cancer, vii. 166.

FEATHERS, danger of beds and pillows made of, ii. 394.

FEBURE, Professor, review of his Recherches et Decouvertes sur la Nature du Fluide Nervieux, vi. 175.

FEBURIER, M. his observations on the ascent of sap in vegetables, xxx. 251.

FECUNDATION, on that of different animals, ii. 245; manner of its taking place, 324; erroneous opinions on the corporea lutea in that process of nature, v. 587; animals capable of it only at particular periods, xviii. 179; on the heat evolved in the process of, xx. 499.

FEES, observations on those of physicians, xi. 182; curious anecdotes respecting those paid formerly, xii. 427, 428; singular letter from a patient to his physician on that subject, xix. 350; anecdotes respecting those of the old physicians in England, xxvii. 115; the charges made in Scotch universities for medical degrees, xxx. 468; proposed plan for the regulation of them, xxxi. 324.

FEET, objections to keeping them warm, i. 259; prejudicial custom of bathing them in warm water, ibid.; new machine for curing distorted ones, by Mr. Robert Watt, with a plate, iv. 93; that invention claimed by Mr. Sheldrake, 192; on the hydropic appearance of the feet in asthma, 262; cases of the cure of the club foot, with engraved representations, 493; reply of Mr. Watt on that subject to Mr. Sheldrake, 498; on the most efficient means of curing distortions in the legs and feet, with cases, v. 9; reply to Mr. Sheldrake on his claim to originality, 119; his answer, 264; Mr. Watt's rejoinder on the same, 426; case of contraction of the tendo achillis successfully treated, 456; farther illustrations, with plates, of the cure of distorted feet, 457; on the practice of delivering by the feet in midwifery, viii. 215; effects of galvanic electricity on the, 321; on the virtue of the campanula convolvulus in swellings of the, xii. 129; case of ampu ation of the foot, without an effusion of blood, 514; on cold applications to them in colic, xiii. 21; case of ruptured tendo achillis, 494; the lichen applied to them by the Laplanders, to prevent excoriations, xx. 267; experiments on cutaneous absorption by immersing them in infusion of madder and logwood, xxxi. 80.

FELIX, Dr. on the use of oil of turpentine in burns, xiv. 398; his practice of copious bleeding in the beginning of typhus, xxviii. 476.

FELLOWES, Sir James, his account of the Gibraltar fever, xxi. 248; on the pestilential winds of the Mediterranean, xxxiv. 406; account of his reports of the pestilential disorders of Andalusia which appeared at Cadiz, 498.

FELTHAM, John, review of his popular view of the structure of the human body, ix. 476.

FEMALES, account of a practical treatise on the diseases peculiar to, i. 310; on the fecundation in different, ii. 246; a female body converted into fat, iii. 10; cause of ruptures in, 73; why peculiarly liable to chlorotic affections, 108, 215; anatomical discoveries in their organs of generation, 157; the greater number of monstrous fœtuses of that sex, 500; enlarged clitoris in them endemical in some countries, iv. 3; operation of amputation practised in such cases, 4; on their intemperance, 15; free from angina pectoris, 30; spontaneous combustion peculiar to them, 45; their superstitious propensity, 147; on the treatment of those who have a distorted pelvis, v. 312; on the uses of the corpora lutea in the ovarium, 587; more liable to dolor facei than men, vi. 160; how affected by venereal complaints, 183; pregnant women affected by phlegmasia dolens, and why, 228; on the cause of convulsions during pregnancy, 280; effect of emetics on those of irritable tempers, viii. 68; on the causes of barrenness in them, 237; on the functions of the placenta in pregnancy, ix. 259; on the constitutional diseases of women, 279; on the extraction of the stone from women, xi. 16; operation upon them in cases of crural hernia, 53; description of the Highland women, xiii. 29; malformation of the genital organs in a female child, 179; on the malformation of genital organs, 180; one born without a bladder, 575; remarkable tumour in the abdomen of one, xiv. 79; on the menstrual discharges, xv. 187; on the manner in which nutriment is conveyed from the mother to the fœtus, 229; on the supposed influence of the imagination of the mother, 249; on the formation of the human ovum, 271; fœtus found in the abdomen of an old woman, 276; remarks on the case, 277; case of an ossified fœtus and uterus in a woman sixty years old, 279; origin of burning the placenta, xvi. 54; observations on superfœtation, xvii. 489; on the opinions of Dr. Osborn respecting the necessity of pain and difficulty in human parturition, xviii. 450; on the constitution of women in different countries, 451; observations on the difficulty of parturition, 545; accused of plagiarism, xx. 181; cases of the principal diseases of the female organs of generation, xxi. 29; wretched state of those employed in the stocking manufactories, 426; case of artificial dilatation of the urethra, xxii. 155; importance of attending to the functions of the uterus, xxiii. 518; singular case of vicarious menstruation, xxv. 24; proportions of deaths in child-bed to births, 214; case of morbid enlargement of the clitoris, 236; description of those of Madeira, 426; peculiar disease among them in that island, 427; remarks on the treatment of sore nipples, 445; extraordinary peculiarity in a female Hottentot, 385; farther account of the same object, xxvi. 16; a fœtus contained in the body of one fifty-two years, 167; disorders among those of Iceland, xxvii. 123; their longevity in that island, 126; on the diseases of their organs of generation, 139; pathology of the female organs of generation, 141; observations on leucorrhœa, 142; on excoriations in them, 143; case of abortion from diseased placenta, 168; on the causes of sudden death in pregnancy, 181; singular custom among some women after child-birth, 381; facts and observations on quickening, 441; on ascertaining the time of conception, 443; farther remarks on quickening, xxviii. 41; extraordinary instance of premature puberty in a female child, 58; account of the introduction of the term man-midwife, 100; impropriety of that practice, 101; extraordinary history of worms in the bladder of one, 154; peculiarity in the menstrual blood of a woman, 402; on the earliest period of gestation when a child may be born alive, 432; cases of prolapses of the uterus, xxix. 84; synopsis of the different kinds of difficult parturition, 423; account of a woman born of white parents, part of whose skin was black, 508; obstructions rare among those of Lapland, xxxi. 52; proportion of ruptures in, 169; the sexual connexion an intellectual passion in human nature, 213; on the female organs of generation, 214; recipe for chapped nipples, 258; practice of midwifery among the American Indians, 303; observations on those diseases of females which are attended with discharges, xxxii. 161, 239, 336; extraordinary account of spontaneous combustion in one, 164; uncommon disease, by which the sexes are apparently changed, 165; why madness is more frequent among them than with males, 241; substance extracted from the vagina of an old woman, 249; account of a disease affecting the extremity of the meatus urinarius of the female, 427; account of a woman in whom a fœtus was retained fifty-two years, xxxiii. 65, 147; the imagination of the mother no cause of malformation in children, 155; cases of hernia in females, 245; on cancer in the breasts of, xxxiv. 151; parturition easy among the women of Sicily, 186; description of the wax figure of one in an advanced stage of pregnancy, 187; practice of man-midwifery reprobated, 290; the practice vindicated, 450.

FEMORAL ARTERY, on the connexion between it and the axillary, ix. 177; cases of amputation, where it was successfully compressed at the groin, 499; another case of the same, xvi. 14; successfully tied the third time, xxvii. 177; case of popliteal aneurism, in which it was obliterated by a peculiar operation, xxxiv. 116.—See *Aneurism.*

FEMUR, case of lacerated, vi. 127; successfully treated by securing the artery, 128; on the dislocation of the, x. 88; case of luxation of the, 494; case of its luxation from an apparently slight cause, xviii. 475; mode of preventing the shortening of the limb in the case of broken, xxx. 516; on the proper treatment of dislocation of the, xxxii. 473; a seton introduced between the fractured extremities of the, xxxiii. 507.

FENCE, on the discovery of a membranous one in the urethra, xxv. 268.

FENNEL, *(anethum feniculum,)* a remedy in consumption, i. 384; botanical description of the, xii. 555; promotes the secretion of milk, ibid.; distilled water prepared from the seeds, ibid.

FENS, on the insalubrity of those of Essex, i. 255; farther inquiry into the causes of their insalubrity, vi. 493; observations on the atmosphere of, vii. 113; description of those of Lincolnshire, viii. 222; on the causes of their unhealthiness, 224; consumption common in fenny districts, 225; the sources of febrile diseases, xviii. 560; prevalence of agues in those parts of England, xx. 479; on the frequency of intermittents in the fenny parts of England, xxi. 91; horses affected by tertians there, 92; the papilio cratægi supposed to be connected with the diseases of the, ibid.; whether the rot in sheep originates in the, 93; description of the great level, ibid.; account of plants and insects peculiar to the, ibid.; account of an epidemic in the fenny lands near New York, xxiv. 365; on the birds peculiar to the English, xxvii. 380; on the alluvial soil of those of Lincolnshire and Cambridgeshire, xxx. 15.

FENWICK, Dr. on the oil of turpentine in the cure of tape-worm, xxviii. 38, 82.

FERBOR, M. his observations on the catacombs of Naples, iv. 114.

FERGUSON, Dr. Andrew, his essay on fever announced, v. 200; his observations on the diseases of the tongue, 247; on the efficacy of cold water in ulcers, vi. 19; description of the cell of Pananach, ibid.; account of the chalybeate spring at Aberdeen, 22; on the fevers of that city, vii. 321; on the deafness peculiar to blacksmiths, 423; his description of the continued fever, x. 232; his case of chancre cured in a new method, x. 499; on febrile contagion, xiii. 37; his observations on leeches, 299; case of ascites, 303; on the human temperaments, xxi. 198, 200; on the epidemic measles at Aberdeen, 359; objections to his opinions, 366; his explanation, 528.

——————, Hugh, his report on vaccination in Ireland, xviii. 103.

——————, William, his method of treating dysentery with calomel, xxviii. 34; observations on the same, 67; his observations on the venereal disease in Portugal, as affecting the constitutions of the British soldiers and the natives, xxxi. 496; character of that work, xxxiv. 235.

FERMENTATION, process of that of vegetable substances, i. 173; effects of electricity on that of vegetables, 200; injurious effects of fermented liquors, 260; vinous fermentation known to the ancients, xi. 411; difficulties attending it, 412; on the causes of vegetable, ibid.; how the saccharine matter is converted into alcohol and carbonic acid, ibid.; on that of the gooseberry juice, 413; substance found in it, 415; animal matter deposited in every kind, 416; ferment contains a quantity of carbon, 417; action of potash upon animal matter, ibid.; important effect of fermentation on sugar, 418; experiments on the same, ibid.; oxygen abstracted from sugar, 420; a new theory of ferment, 421, *note*; prize question on that subject, xii. 96; in the stomach the cause of diarrhœa, 106; fermented liquors the cause of arthritic and gravelly complaints, xvi. 164; albumen found in vegetable juices, which ferment without yeast, xvii. 198; new instrument for ascertaining the quantity of saccharine matter in unfermented wine, xx. 482; on the fermenting principle in the making of bread, xxii. 404; preparations instead of yeast, 405; the white of champignons undergoes fermentation, xxiii. 69; the datura used by the Chinese to ferment liquors, xxv. 383; on the existence of alcohol in fermented liquors, xxx. 392; alteration produced by it in rocou, xxxii. 435.—See *Yeast*.

FERMOR, William, his reflections on the cow-pox, with cases, iv. 257; extracts from his reports, 434; his example recommended, v. 17; on the existence of the cow-pox in different parts of England, 112, 118; his observations on failure in vaccine inoculation, and the causes, vi. 323; letter of Dr. Jenner to, 325; his cases of secondary small-pox, xii. 167; character of him, xvi. 131, 539; the first patron of vaccination, 540; his letters to Mr. Ring, 541, 544.

FERNELIUS, his anatomical discoveries, i. 396; on the bite of the viper, xi. 484; his notice of the changes sustained by the different organs of the body, xvi. 451; his observations on hereditary diseases, xxi. 229; on the propagation of elephantiasis, 287; on the causes of diseases, 331; on hereditary consumption, 381; on the lues venerea, xxix. 207; on diseases of the liver, xxxi. 430; in pemphigus, xxxii. 282.

FERNS, discovery of a new species of, iii. 284; description of the several kinds of them under the general denomination of Trichomanes, v. 372; botanical description of the female fern, (pteris aquilina,) xix. 542; used by the ancients in diet-drinks, ibid.; a popular remedy for worms and rickets, ibid.; used in Siberia instead of hops, for brewing ale, ibid.; a good lye for cleansing linen, 542; description of the polypodium, or male fern, xx. 157; its root a powerful vermifuge, 158; method of administering it, ibid.

FERRI, Alphonsus, his opinion on the poisonous nature of gun-shot wounds, ii. 56.

FERRI SULPHAS, form of preparing it, xxxiv. 495.

FERRIAR, Dr. John, on the success of digitalis in hæmorrhage, ii. 102; observations on his cases, iii. 202; on digitalis in hernia, 332; on croup, 539; on the appearance of dropsy in phthisis, iv. 243; his zeal for the Fever Institution at Manchester, 396; his reply to Dr. Hull on a case of phlegmatia dolens, v. 4; answer to him, 165; on the locality of diseases esteemed contagious, xiv. 342; his observations on hysteria, 395; on the conversion of diseases, xv. 131; on the powers of the digitalis in reducing the pulse, xvi. 231; on hydrothorax, 356; defended from the charge of plagiarism on the subject of the conversion of diseases, xx. 181; his definition of mania, xxi. 497; on digitalis in consumption, 327; on the virtue of the same in the reduction of the pulse, xxii. 93; recommends arsenical solution in hooping-cough, xxv. 115; on the use of digitalis, 116; on the virtues of tobacco as a diuretic, 233; his two cases of croup, xxvii. 199; his practice of depletion in that disorder censured, 208; his case of rabies, xxviii. 250; on calomel in mania, xxix. 212; on his cases of gastritis, xxx. 203; review of the fourth volume of his Medical Histories and Reflections, xxxi. 143; on the efficacy of elcterium in general dropsy, 144; benefit of the tonic plan of treatment in diabetes, 146; on the theory of that disease, ibid.; on the cause of phlegmasia dolens, xxxii. 190.

FERRO, Dr. of Vienna, adopts vaccination in his family, ii. 285; on the efficacy of the warm-bath in nervous fever, 466; on the treatment of petechiæ sine febre, x. 444.

FERRUM TARTARIZATUM, composition of a new solution of, xxviii. 244; beneficial in scrofulous cases, 245.

FERUSSAC, M. his discovery of fossilized shells in France, xxix. 520.

FESTUCA, or flote fescue grass, botanical description of, xi. 528; a good vermifuge for horses, ibid.

FETOR, causes of that of the human breath, ii. 464; method of correcting that of air affected by putrid meat, viii. 187; that of stools ascribed to bile, xii. 105; the cadaverous fetor in gangrene subdued by nitre, 483; on the fœtid grains in the sputa of consumptive patients, xiv. 30; fœtid vapour prevalent in China, xxiii. 283; peculiar one in the smut of wheat, xxiv. 37; offensive one emitted by the rattle-snake, 392; on that of dead bodies, 423.

FEVER, practical observations on the benefit of cold air and water in, i. 18; on catching cold in, 19; effects of abstinence in preventing, 44; cautions observed at sea to prevent, 94; on the action and use of the Peruvian bark in intermittents, 104; memoirs respecting the cure of a contagious typhus fever in Tuscany, ibid.; on the causes and cure of remittent bilious fevers, 105; on the different states of excitability in fever, 127; on the stimulating treatment in it, 128; prevalence of scarlatina anginosa in London, 135; on the causes and symptoms of the marsh fever, 139; caused by noxious exhalations, 140; its effects on different persons, ibid.; method of treatment at sea, 141; substitutes proposed for bark in intermittents, 187; account of a German translation of Dr. George Fordyce on fever, 200; debilitating effects of fear in febrile complaints, 202; prize question at Guy's Hospital on the subject, ibid.; an outline of the history and cure of contagious fevers, 207; analysis of Dr. Jackson's treatise on idiopathic fever, 225; observations on the fevers of the West Indies, 226; symptoms of the fever that appeared among the troops in Holland, 227; account of an essay on fevers, by Dr. Robert Robertson, 227; on the contagious fever of the British army in Holland, ibid.; fever among the troops at Cork, 228; on the endemic fever of St. Domingo, 229; the intermittent fever a variety of the endemic, caused by marsh miasmata, 236; means of purifying the air from contagion, 245; on the causes and cure of remitting bilious fever, 246; soils favourable to intermittents, 258; on the febrifuge virtues of lime, magnesia, and alkaline salts, 274; observations on the fever at Bombay, 348; abstract of the theory of puerperal fever, 381; various modern opinions on the same subject, 386; method of treatment, 388; efficacy of mineral and vegetable acids in typhus gravior, 394; on the changes produced in the air by the disorder, 397; singular case of petechiæ unattended with fever, 420; benefit of opiate frictions in typhus, 440; method of guarding against infectious fevers, 457; on contagions producing fever, 461; method of cure employed at Bombay, 462; on the remote causes of fever, 467; infection attributed to marsh miasmata, jails, hospitals, and ships, ibid.; endemic fever prevalent in luxuriant vegetation, 468; on the contagious fever of cities, 469; cases of contagious and endemic fever, 470; symptoms of febrile disease, ibid.; symptoms where impaired action is conspicuous, 471; symptoms where particular functions are deranged, 472; on irritated motions, 475; symptoms of endemic fever, 477; on fevers of type, 482; on the cure of fever in its different stages, 484; the hepatitis called a fever complaint in India, ii. 6; benefit of opiate friction in typhus, 103; instance of the same in inflammation, 107; prevalent among the poor, 112; analogy between hydrophobia and malignant, 174; benefit of bleeding in guarding against, 178; that practice controverted, 179; produced by septic fluids, 180; on the affinities of septic or pestilential fluids to other bodies, ibid.; on the non-existence of typhus contagion, 194; venesection censured, 234; benefit of opium in fever, 235, 236; formulary of administering it, 238; review of Dr. Wilson's treatise on febrile diseases, 300; on the proper manner of discussing the subject in medical enquiry, 338; opinions of the ancients on the causes of fever, ibid.; Dr. Miller on the treatment of febrile diseases, 373; efficacy of yeast observed in some cases of fever among children, 374; review of Dr. Fordyce's third dissertation on fever, 393; prevalence of typhus in London in 1799, 411; symptoms and treatment, 412; observations on the causes of the mesenteric fever, 423; opinion of Dr. Cullen on that complaint, 424; and of Dr. Darwin, who divides it into classes, ibid.; on hectic fever, 426; typhus ceases with frost, iii. 16; cases of the benefit of cold affusion in fever, 52, 55; on the sweatings in recovery from fever, 58; medicinal effects of carbonat of potass in puerperal fevers, 80, 165, 264, 363; observations on bilious fevers, 94; on the increase of febrile diseases in London, 109; fever produced by the application of venereal poison, 198; punch recommended instead of wine in typhus, 217; bleeding defended in fever, 226; on the inflammatory symptoms in pneumonia, 230; on the fevers among the poor, 298; proposals for preventing them, 300; benefit of cold affusion, 304; nitrous fumigation exploded, 322; on the treatment of nervous fever, 326; decrease of malignant fever in London, 393; mode of treatment, 394; benefit of the bark in childbed fever, 442; utility of nitrous fumigation in destroying contagion, 484; that opinion controverted, 526; beneficial effects of yeast in typhus fever, 533; venesection in fever censured, 538; benefit of cold water in scarlatina and cynanche, 556; account of the infectious malignant fever at Uxbridge in 1799, 561; account of the bilious malignant fever in New Hampshire in the same year, 568; an empirical remedy for all kinds of fevers discovered in Prussia, 586; beneficial effects of cold water in fever, iv. 26; general observations on febrifuge medicines, 51; effect of nitre in fever, 54; two instances of intermitting fever cured by flores arnicæ, 85; discovery of a specific for scarlet fever, ibid.; success of cold affusion in typhus, 105; malignant fever generated in a crouded ship in the Thames, 113; considered by Hippocrates as an effect of nature to get rid of a load, 151; his method of treating fevers, 152; cases of typhus, with delirium, 202; its effects on the brain, 203; on the malignant fever of Grenada, 284; historical notices of a catarrhal fever on board the Bellerophon, 290; review of a practical treatise on the different fevers of the West Indies, 354; observation on the jail fever, 356; mortality in London from continued fevers, 395; fever hospital projected, 396; historical sketch of the use of cold water in fever, 397; additional testimony of the efficacy of yeast in typhus, 401; difficulty of ascertaining the origin of fever in some cases, 491; observations on the use of yeast in typhus, 539; account of professor Reich's theory and treatment of fever, 556; oxygen the only sure remedy in fever, iv. 560; v. 178; remarks on the external use of cold affusion in fever, v. 12; prevalence in London of an alarming typhus, 14, 16; benefit of cold affusion in fevers of the low kind, 75; universality of the febrile contagion, 97; on the prevalence of fevers at Liverpool, 118; uncertainty of the theory and practice of this class of diseases, 130; variations in the mode of treatment, 132; the symptomatic fever of consumption yields to the foxglove, 133; caution recommended in the use of yeast as a remedy in fever, 143; description of a contagious fever in London, 145, *note*; muriatic acid given internally, 178; on the use of the acids in fever, 179; quantity requisite for a cure, 180; inconveniencies in the use of mineral acids, 181; on the malignant pestilential fever of the coast of Guinea, 184; increase of typhus in London, 194; on the delirium in fever, 198; case of pure inflammatory fever, 232; general success of the use of yeast in putrid fevers, 270; on the situation of the poor in the metropolis, with a plan for the institution of houses of recovery, 289; outline of the plan of a fever institution, 290; determination to the head a characteristic of fever, 301; Mr. Carlisle on his plan for the establishment of houses of recovery, 305; prize question on the cause of heat in fever, 314; comparative view of the theories of Cullen, Brown, and Darwin, on fever, 388, 577; decrease of catarrhal fevers in London, 393; prospectus of a new work on idiopathic fever, 400; beneficial effects of yeast in typhus, 422; on the classification of intermittents, 483; on the term hectic, as used by the Greek physicians, ibid.; description of the febris aphthosa, ibid; cases of the efficacy of yeast in typhus, 508; symptoms of a typhus in London, 510; explanation of Dr. Darwin's theory of fever, 577; on the utility of cold water in fever accounted for, 578; opium and wine recommended, 579; on the prevention of fever, 580; on the prevention of infections, 582; children affected by the blue fever, 586; institution for the prevention of contagious fever among the poor, 589; uncommon occurrence of fever in the metropolis, vi. 8; observations on the use of acids in fever, 87; cold vinegar successfully used externally in typhus, 107; ice and cold water beneficial in fever, ibid.; prevalence of fever in London, 111; the efficacy of yeast farther evinced, 112, 196; the synoche biliosa prevalent at Bath, 195; method of treating it, 197; on the prevalence of typhoid contagion, 198; review of a treatise on Febrile Diseases, by A. P. Wilson, M. D. 255, 365; on the proximate cause of fever, 256; case of typhus followed by amentia, 296; use of the antimonium tartarizatum in preventing bilious fever, 310; account of a preservative remedy against scarlet fever, 367; Dr. Currie on the practice of cold affusion in fevers, 381; efficacy of that practice proved in the barracks at Gosport, 382; advantage of the tepid affusion in topical inflammation, 385; benefit of opiate friction in, 387; diseases of the stomach attendant on typhus, 389; prevalence of the scarlet fever at Kettering, in Northamptonshire, 420; analysis of Hahneman's powder for scarlet fever, 475; on the effects of opium in febrile cases, 479; discharge of blood in synochus, 491; dysentery combined with typhus, 492; why flat and marshy situations are favourable to fevers, 493; calomel and James's powder recommended in the early stages, vii. 38; efficacy of affusion of vinegar, 39; observations on typhus fevers at Great Yarmouth, 54; the cortex soimydæ given in intermittents, 81; inquiries into idiopathic and combined fevers recommended, 96; on the effluvia produced in fever, 255; benefit of vinegar, ibid.; on the power of communicating contagion in fever, 256; on the new mode of treating fever, proposed by Reich, 262; profuse bleeding in fever, an ancient practice, 311; on the pestilential fever at Cadiz, 312; on the mercurial treatment of fever, 314; how pestilential fevers are affected by the atmosphere, 317; account of an autumnal fever at Aberdeen, 320; treated with sudorifics, 323; on the exhibition of vegetable acids in fever, 337; their advantages in typhus, 338; washing with vinegar recommended in fever, 341; observations on irregular intermittents, 376; ten varieties of the irregular intermittent, 377; caused by stagnant exhalations, ibid.; efficacy of bark, ibid.; accounts of pestilential fevers in crouded ships, 381, 383; persons employed in mercurial mines seldom affected with fevers, 403; use of mercury in fevers known to the ancients, 404; claim of Dr. Johnstone to the discovery of the power of mercurial acid vapours to correct contagion, 424; general classification of antiseptics, 565, 572; human effluvia the causes of fever, viii. 50; confirmation of the efficacy of acetous ablution in typhus, 51; benefit of opium in contagious fevers, 52; that remedy not a new one, 53; benefit of old Madeira in, 89; heat the cause of typhus, 158; improper mode of treating it, ibid.; on the most efficacious mode of applying cold in fever, 160, 357; review of a treatise on the prevention of contagion, 185; prevalence of typhus in Lincolnshire, 222; review of Dr. Fordyce's fourth Dissertation on Fever, 274; on the change of type in intermittents, ibid.; on the sensation of hardness in the pulse, 275; advantages of blood-letting, 276; description of the puerperal fever, 433; variety of its symptoms, 434; appearances on dissection, 435; difficulty of accounting for it, 436; observations on lacteal metastases, ibid. 437; different from inflammatory diseases, 438; a morbid accumulation of the milky matter in the blood, 439; produced by colds, 441; stimulants to be cautiously used, 442; on the pestilential and malignant fevers of the Levant, 449; practical information on the malignant scarlet fever, 471; solution of ammonia efficacious in that complaint, ibid.; the tincture of valerian and salt of tartar serviceable in puerperal fever, 510; injections into the uterus recommended, ibid.; on the use of camphorated liniment in puerperal fever, 511; on the treatment of the breasts, 512; cases of puerperal fever, 514; on the means of purifying the atmosphere from contagious matters, 529; plan adopted for the prevention and cure of contagious fever in the metropolis, 538; collection of papers to promote a fever institution at Newcastle and other populous places, 562; extraordinary success of the practice of cold affusion in the navy, ix. 9; uncertainty in regard to the cause of fevers, 28; power of mercury in counteracting fever, 31; necessity of phlebotomy as a preparation, 32; on epidemic scarlet, 157; on the prevention and cure of, 182; cases of typhus, 206; effect of digitalis in scarlatina, 249; tincture of opium efficacious in synochus and typhus, 269; why continual fever sooner cured than intermittents, 270; observations on the milk fever, 281; cases of typhus cured by cold affusion, 282; account of a catarrhal fever at Paris, 287; the phlogisticated nitrous air the cause of pestilential fevers, 304; intermittents originate in asthenia, 305; method of treating the quotidian fever, 306; prize question on the frequency of catarrhal and other fevers, 372; prevalence of catarrhal fever in London in 1803, 375; treated with cathartics and emetics, 376; observations on the epidemic and catarrhal fever, 377; further remarks on the same, 385; description of its appearance, 386; moxas applied with success in loss of speech after fever, 389; report on the catarrhal fever in France, 419; varieties of that disorder, 420; cured by mercurial frictions, 425; the jail fever and plague the same disease, 502; observations on the pestilential fevers of the East Indies, 503; the practice of ablution a preservative from fever in Hindoostan, 504; means of diminishing contagion, ibid.; on the prevailing epidemics of 1803, 509; on the frequency of scarlet fever, 511; symptoms of the prevailing epidemic, 512, 516; Dr. Kinglake on the same, 517; Dr. Bardsley's account of the epidemic catarrhal fever at Manchester, 522; general remarks on the same, 527; glue a remedy in, 567; account of an eruptive fever in Egypt, 578; on the seven alleged causes of puerperal fever, x. 11; milk fever different from puerperal fever, 13; danger of bleeding, 14; utility of emetics, 15; on the use of opium, 16; diaphoretics only serviceable in the early stage, ibid.; on the use of blisters, 17; on the proper administration of bark, 18; observations on the fevers of hot climates, 36; the favourable and unfavourable symptoms, 37; on the discovery of mineral acid vapours to destroy contagion, 78; account of the remittent fever on the coast of Africa, 85; collections for a history of the epidemic fever in England, 97, 127, 129, 193, 231, 232, 233, 289, 303, 312, 355, 385, 517; xi. 488; on the use of gelatine in intermittents, x 323; history of the autumnal fever which occurred in 1802 at Wilmington, in North America, 365; case of variety of intermittent fever, with symptoms of hydrophobia, 427, 541; observations on petechiæ without fever, 444; cases of that complaint, 446; account of a fatal case of typhus, 456; facts and observations on the prevention and cure of scarlet fever, 457; observations on the nettle-rash fever, 481; description of that complaint, 482; effects of eating muscles, 483; benefits of the cold ablution observed in the Mediterranean, xi. 21; cases of scarlatina relieved by that practice, 27; on the contagion of the influenza, 81; on the means of preventing contagion, 97; effect of the nitrous vapours in arresting the progress of contagious fevers, 136; on the proper treatment of fevers in hot climates, 201; caution to be used in regard to bleeding, 202; observations on the lunar influence in fever, ibid.; free evacuations to be promoted, 203; opiates censured, ibid.; on the febrifuge principle of cinchonin, 215; utility of the oxygenated muriatic acid in scarlet fever, 289; new theory of puerperal, 383; benefit of the yellow bark in, 444; friction with oil found useful in the fevers of the West Indies, 480; xii. 283; utility of opium in intermittents, xii. 3; observations on the scarlet fever and sore-throat, 25; the alkalies ineffectual in puerperal, 28; benefit of mercurial inunction in typhus, 52; ravages of fever in England, 78; bark only necessary in particular stages, 153; report of the Committee of the House of Commons on the Fever Institution of London, 161; efficacy of yeast in typhus, 192; singular case of brain fever, 196; benefit of the centaury in, 221; and currants, 225, 226; observations on animal heat in fever, 244; in China, 341; cases of putrid fever, 346; oily friction tried with success, 382; on the febrifuge substance in bark, 430; benefit of animal jelly in intermittent fevers, 431; on affections of the head in typhus, 572; fevers of rare occurrence in the Highlands of Scotland, xii. 37; essay on the origin of the epidemical fever in Spain, 103; circular letter from the committee of the Fever Institution of London, 190; queries on contagious fever, 191; eight cases of the malignant fever at Gibraltar, 193; on the nature and origin of catarrhal fever, 209; all epidemics ascribed to sensible qualities in the air, 210; catarrhal fever among horses, 216; outlines of a plan to prevent malignant contagion, 272; proposals for Committees of Health, 273; on the causes which produce fevers, 275; cases in the Military Hospital at Canterbury, xiii. 318; observations on febrile affections, 376; on the use of bleeding in fever, 377; that practice necessary in inflammation, 472; on the emetic class of remedies in fever, 474; account of a catarrhal fever in China, 488; review of Dr. Wilson's fourth volume on Febrile Diseases, 554; virtue of bathing in fever, 564; efficacy of mercurial inunction in fever, xiv. 38; the benefit of cold bathing caused by the state of excitability, 85; rules for measuring heat, 86; the importance of bathing in the cure of fevers, 87; on fevers of the periodic class, 88; action of contagious fever on the skin, 89; caution in regard to bathing in some cases, 90; benefit of bathing in intermittents, 101; account of the Gibraltar fever, 137; directions for the use of bathing in fever, 179; benefit of gestation in fever, 180; Board of Health established for the prevention of, 191; farther account of the fever at Gibraltar, 201; on the symptoms and treatment of the same, 202; cold affusion strongly recommended, 203; method of destroying contagions, 205; cold affusion in catarrhal fever successfully practised in the Orkneys, 226; observations on epidemic fever and its causes, 338; report of the London Institution for the Prevention of Fever, 340; description and history of the scarlet fever, 368; use of caustics and blisters in that disorder, 371, 373; benefit of the spurge-laurel, or daphne laureola, in rheumatic fevers, 545; on the combination of dysentery with typhus, 560; on the discovery of mineral gases for destroying contagion, 573; on the fever at Up-Park camp, in Jamaica, xv. 41; observations on the nature and cure of fevers and diseases in the West and East Indies, 83; utility of emetic injections, 85; benefit of purgative medicines in typhus, 183; account of a remittent fever among the troops in this climate, 189; comparative fatality of the fever, 190; utility of frequent bleeding in it, 191; fatal case of typhus, 240; appearance on dissection, 241; benefit of wood-sorrel in fevers, 267; virtue of common agrimony in, 270; on the cure of intermittent fever by ammonia, 333; on the discovery of the mineral acids as a preventive of contagion, 373; account of a malignant fever at Quebec, 446; cases of scarlet fever and sore-throat, 485; observations on the scarlatina anginosa, 489; the free use of bark in fever, recommended, 494; description of the synochus, xvi. 186; obstructions of the spleen in, 195; observations on the typhus in Derbyshire, 236; benefit of cold affusion in it, 217; strawberries recommended in fevers, 264; the common avens, or geum urbanium, a good febrifuge, 267; report of the Board of Health at Manchester, 338; on the benefit of cold as a remedy in fevers, 370; on summer fevers, 382; use of emetics in fevers, 432; efficacy of opium in fever, 561; favourable cases of typhus, 572; description of a mild kind of that disorder, xvii. 77; report of the malignant fever at New York, 97; account of the Lake Fever of North America, 114; account of the fever of Minorca, 125; account of the scarlatina, 186; prize questions on epidemic fevers, 198; fatal effects of septic gas on a family, 225; on the cure of fevers arising from marsh miasmata, 447; on the cause of fever, 451; utility of common germander in intermittents, 467; on the cure of scarlatina, 470; account of the efficacy of yeast in typhus, 503; observations on febrile contagion, 505; efficacy of antimonial powder in, 551; cases in the Fever Hospital in Dublin, 553; on the difficulties attending the doctrine of fever, xviii 8; account of a remittent fever, and its resemblance to typhus, 9; remedied by bleeding, ibid.; on the epidemic disease of Gibraltar, ibid.; review of an Enquiry into the Seat and Nature of Fever, by Henry Clutterbuck, M.D. 74; the brain alleged to be the seat of fever, 75; on the nature of febrile action, 76; analogy between fever and inflammation, 77; history of an epidemic low fever, ibid.; the brain proved to be the principal seat of the disease, 78; on the diagnosis of fever, ibid.; on the advantage of blood-letting, 79; on topical inflammation, 80; on the manner in which fevers supersede each other, 86; historical accounts of the jail fever, 89, 90; the typhus proved to be contagious, 92; case similar to brain fever succeeding a burn, 152; another case of brain fever, 153; advantage of refrigeration in fever, 197; benefit of rum in that practice, 198; peculiarity attending convalescence in a case of synochus, 285; a case of fever and pain from rheumatism, relieved by electric sparks from the body, 362; observations on contagion and fever, 423; inutility of quarantine laws as a preventive, 425; review of an Essay on the Nature of Fever, with an attempt to ascertain the principles of its treatment, 462; observations on the proximate cause of fever, 463; the doctrine of Dr. Cullen on that subject stated, ibid.; objections to the Brunonian doctrine, 464, 466; fever denied to be contagious, 497; on the different causes of fever, 498; how far bad air produces epidemics, 499; volcanic eruptions the cause of febrile diseases, 500; cases of intermittents cured by calomel, xix. 15; case of intermittent fever cured by nasal hæmorrhage, 53; the milkwort beneficial in fevers, 73; case of acute bilious fever successfully treated by nitric acid, 106; case of the successful use of mercury in typhus, 110; utility of the cold bath in fever, 111; on quarantines, contagion, and fever, 113; on fever as connected with inflammation, 168; the brain supposed to be the principal seat of the disease, 169; remarkable case of Dr. Goldhagen, 171; observations on debility in fevers, 174; little dependence on cold affusion, 175; on the diagnosis between fever and phrenitis, 179; fevers have their origin in bad air, 199; on the causes of sporadic air, 201; two cases of fever induced by the bite of a venomous insect, cured by copious depletion, 220; origin of the practice of cold affusion, 271; on the advantage of diminished excitement in fever, 304; inefficacy of mercury in typhus, 316; on seasons peculiar to fevers, 317; on the occasional recurrence of typhus, 322; the bark unsuccessful in typhus, 323; on the proximate causes of fever, 334; a particular kind of typhus, with petechiæ, described, 366; use of mercury defended, 451; benefit of the metricaria parthenium, or feverfew, in those diseases, 457; efficacy of camomile in fevers, 459; mercury reprobated in typhus, 522; on contagion in febrile diseases, xx. 27; general review of publications on the subject of fevers, 35; beneficial effects of gestation in typhus, 36; remarkable history of the efficacy of cold affusion in fever, 51; exposition of the practice of affusing cold water on the body as a remedy for the cure of fever, by Robert Jackson, M.D. 81; on the claims of Dr. Wright to the discovery of that practice, ibid.; observations on those of Dr. Currie to the same, 82; history of the practice of cold bathing as a remedy for fever, ibid.; account of Dr. Jackson's own practice, 83; Dr. Gregory's notes on the effects of bathing in different kinds of fever, 85, *note*; observations on scarlatina anginosa, 121; on the administration of mercury in typhus, 145; fever described as an alteration in the organic actions of the system, 164; authorities for free bleeding in fever, 165; emetics and purgatives recommended, ibid.; cold affusion peculiarly serviceable in typhus, ibid.; minute examination of the various forms of infectious fever, 166; effects of cold drink and immersion in fever, 167; case of Alexander the Great, 169; general account of the good effect of that practice, 170; observations on the nature of inflammation and its connexion with fever, 174; the hot stage of fever a general state of inflammation, 177; account of a particular kind of febrile complaint at Edinburgh, 191; on the efflorescence produced in typhus, 279; on the petechial spots in typhus, 281; modus operandi of alkalies in fever, 346; on the epidemic of Gibraltar, 368; account of the Oxford Black Assize, 370; the erysipelas a febrile disease, 371; dissections of persons who died of fever, in order to ascertain the state of the brain, 412, 427; account of the sweating sickness, 473; frequency of fevers at Edinburgh, 478; farther cases to shew that the brain is not the seat of fever, 540, 542; improved method of applying cold in fever, 562; connexion between the state of the atmosphere and fevers, xxi. 16; on the principal exciting cause of typhus, 17; typhus in all forms contagious, ibid.; on the pre-disposing causes of typhus, 18; relation between scurvy and fever, ibid.; cases in the Swedish fleet, 20; on the fevers of hot climates, 71; influence of the moon in fever, 75; necessity of a change of air in fever, 77; on the benefit of sea air in fevers, 78; on the atmospherical influence in fever, 91; causes of intermittents in fenny countries, 92; new theory of the proximate causes of fevers, 102; proofs of the same from the history of diseases, 105; peculiar appearance of the eyes in fevers, 108; the utility of emetics and bleeding in fever, 119; on bleeding and purgatives in, 169; proofs of the contagious properties of those diseases, 241; diaphoretics and external irritations injurious in scarlatina, 253; benefit of emetics and purgatives in that complaint, 254; on the causes of, 296; benefit of mercury in, 326; appearances of typhus at Edinburgh, 333, 433, 517; virtue of the spider's-web in intermittents, 353; observations on intermittents, 441; efficacy of arsenic in the same, 442; appearances on dissection in fever, 449; case of scarlatina, 528; additional observations on the proximate causes of fevers, xxii. 36; use of the cold bath in fever, 43; the antimony injurious, 41; hydrophobia compared to a malignant fever, 59; virtues of the spider's-web, 95; on the nature of fever in general, 99; observations on the seat of fever, 100; the causes of fever too much simplified, 101; inflammation both a cause and consequence of fever, 102; on the connexion between the atmosphere and, 105; connected with stricture of the urethra, 129; prevalence of intermittents in London, 190; queries and hints on that subject, 191; doubts on the virtue of the spider's-web, 218; proofs of a new theory of fever from the practice of physic, 227, 296, 458; benefit of depletion in fever, 228, 298; utility of the cold bath, 230; cases of malignant fevers, 347; benefit of cobweb evinced, 369, 370, 457; free bleeding recommended in remittents and intermittents, 459; efficacy of the tourniquet in preventing the cold fit of an intermittent, 461; utility of emetics in warm climates, 463; on the virtue of mercury in fevers, 464; on the influence of the mind in fevers, ibid.; case of intermittent fever, accompanied with sympathy of organs which have no sensible connexion with each other, 497; observations on the contagious properties of fever, 513; memoir on a contagious disease which prevailed in the French hospitals in Spain, xxiii. 33; treated with volatile stimulants, 37; accompanied with diarrhœa, 38; efficacy of muriatic acid in scarlatina and typhus, 51, 53; facts establishing the efficacy of opiate friction in febrile complaints, 71; decrease of fever in the metropolis, 88; proofs of a new theory of fever, 116; on the utility of wine, ibid.; pure air the best preventive, 117; benefit of smoking tobacco, 118; proofs from the general causes of diseases, 121, 281; remarks on infantile fever, 128; on the symptoms in such cases, 130; cases of infantile fever, 132; on the fever which appeared in the English army in Spain, and on their return home, 155; inefficacy of the cold affusion, 159; case of Dr. Clarke, 165; official report on the fever among the English troops in the island of Walcheren, 183; historical accounts of epidemic fevers, 281; observations on scarlatina, 413; scientific and popular view of the fever of Walcheren, by J. B. Davis, M.D. 418; fatal cases of typhus, 435; history of an epidemic in France, 455; proofs of the new theory of fever from the remote causes of diseases, 469; ascribed to volcanic eruptions, 470; on the planetary influence in fevers, 476; emetics hurtful in remittents, 498; on the treatment of the sick who returned from Corunna, 517; efficacy of bleeding, 518; febrile diseases attributed to animalcule, xxiv. 23; fatal case of puerperal fever, 107; on the causes of inflammatory diseases, 230; benefit of mercury in such cases, 231; remarkable instances of scarlatina, 348; cases of the fever which prevailed in 1809 in Orange county, New York, 365; on the febrile effects of the effluvia of dead animal bodies, 422; on the endemic fever of Sicily, 424; the purgative method of treating fever gains ground in London, 430; mortality among the American soldiers at New Orleans, 435; on the extraordinary quantities of calomel administered in fever, 461; fatal cases of scarlatina, 465; description of an affection of the tibia, induced by fever, with observations on the treatment of the complaint, 501; ravages of the scarlatina maligna in various parts of England, 531; statistical report of the Walcheren fever as it appeared among the troops at Ipswich, xxv. 1; account of a contagious fever on board a ship, 13; benefit of calomel in that case, 21; opium found necessary, 22; ipecacuanha beneficial, ibid.; utility of magnesia, ibid.; efficacy of castor oil, ibid.; on the hectic, 78; cases of scarlatina anginosa cured by venesection, 101, 106; on an epidemic puerperal fever, 193; description of a peculiar typhus among the Mexican Indians, 242; on the variety of practice in the treatment of scarlatina, 297; caution in regard to venesection in that complaint, 298; advantage of the antiphlogistic plan, ibid.; prevalence of the disorder in Norfolk, 299; expediency of establishing foreign hospitals for soldiers, 348; on the antiphlogistic treatment of scarlatina, 407; scarlatina epidemic at Madeira, 430; on general and topical bleeding in febrile diseases, 546; on the causes of the prevalence of typhus, 548; on the extravagant practice of depletion, xxvi. 37; the insidious advances of typhus, 84; on a fever accompanied by extraordinary chilliness, 92; vindication of free blood-letting in fever, 108; contagiousness of typhus, 152; account of a spotted or petechial fever in New England, 217; appearances on dissection, 221; fatal case of fever, 250; frequency of febrile diseases in London, 256; fatal case of puerperal fever, 265; on the proper treatment of that disorder, 269; its insidious attacks, 271; uncertain whether it be infectious, 272; tincture of bark used as a friction in fever, 318; on the febrifuge properties of cinchona, 330; on the discovery of its medicinal qualities, 333; its utility in remittents and intermittents, 334; formula of an excellent febrifuge, 345; on the action of the vessels in a state of inflammation, 353; on the circulation of the blood in parts inflamed, 354; plastic actions in inflamed parts, 358; on the effects of cold affusion in fever, 398; virtue of that practice in febrile diseases, 431; the indiscriminate use of it censured, 433; on the dilatation of the blood vessels in parts inflamed, 435; cases of scarlatina anginosa, 442; benefit of the sponging treatment, 444; fever the most destructive of all diseases, xxvii. 34; objections to the term typhus, ibid.; good effects of bleeding, cold affusion, and sponging, in scarlatina, 35; on two distinct forms of fever, 36; observations on marsh fever, 37; three classes of fevers in Sicily, 66; case of summer fever in that island, 67; on depletion by the lancet, 89; description of two species of scarlatina, 103; method of treatment without bleeding, 106; cases of the complaint, 107; on the contagious fevers of Edinburgh, 166; remarks on puerperal fever, 183; necessity of early blood-letting in that disorder, ibid.; in what cases to be used, 184; appearances of the tongue, 186; on the pneumonia typhoides, ibid.; benefit of depletion in extensive visceral inflammation, 189; on the virtue of opium and its combinations, 192; farther observations on depletion with the lancet, and the use of opium, 284; on the symptomatic fever in dysentery, 292; the febrifuge virtues of different kinds of bark, 303; account of a memoir by M. Chaussier on puerperal fever, 377; on the putrid fever in hospitals and ships of war, 378; review of a treatise on the effects of sol-lunar influence in fevers, by Francis Balfour, M.D. 398; account of Dr. Mead's treatise on the same subject, 399; fevers of all kinds affected by the moon in Bengal, 400; an attention to the revolution of the moon necessary in the cure and prevention of fevers, ibid.; the influence of the moon in fevers general throughout the globe, 402; on the curative and anti-contagious properties of oxygenated muriatic acid, 408; on the advantages of purgatives in typhus, 458; benefit of calomel in that complaint, 462; various hypotheses on fever, 472; caution requisite in the treatment of pyrexiæ, 473; benefit of bleeding exemplified in a remarkable case, 474; that practice serviceable in robust constitutions, 476; the free use of ardent spirits a pre-disposing cause of typhus, ibid.; remarkable case of recovery, 477; depletion the anchor of hope in dangerous cases, 481; account of a fatal epidemic at Blackaton, in Devonshire, xxviii. 23; observations on the report of a case in which typhus was said to be cured by purging, 102; history of a fever which prevailed in the suburbs of Paisley, with an account of the utility of blood-letting, 131; method of treating patients on their recovery from fever, 132; prevalence of scarlatina and typhus at Nottingham, 138; remarks on the fevers of Sicily, 221; the use of purgatives in typhus vindicated, 278; farther justification of that practice, 289; opinion of Cullen on two genera of fever, 291; authorities for the practice of purging in typhus, 292; observations on the theory of fever, 294; on the diminished energy of the nervous system, 295; benefit of cold affusion instanced at Jamaica, 296; utility of calomel and James's powder in fever, 297; how bleeding relieves inflammation, 376; on the extension of the term typhus to general cases of fever, 430; remarks on the use of purgatives in fever, 446; their benefit proved in the West Indies, 417; efficacy of bleeding in the French prison near Bristol, 476; remarkable case of synochus, 511; case of supposed typhus, 519; on the evatuating practice in fever, xxix. 11; account of a spotted fever in America, 14; historical sketch of the use of mercury in fever, 213; classification of fevers, 250; on the spotted fever of America, 328; on the ardent fever of Guadaloupe, Gibraltar, and Plymouth, 412; benefit of liquor arsenicalis in remittents and intermittents, 414; fatal cases of supposed typhus, 527; on the destructive effects of marsh fever, xxx. 14; description of the symptoms of delirium tremens, 17; on the introduction of the depletory method of cure in the tropical fever, 76; on the brain fever produced by intoxication, 77; benefit of laudanum in that case, 78; facts and observations respecting intermittent fevers, and the exhalations which occasion them, 159; on marsh miasmata, 161; on the division of diseases into general and local, 180; on the class of pyrexiæ, 183; on the fever of Bengal, 229; benefit of bleeding and calomel in that case, ibid.; on bilious fever, 231; on the endemic of Batavia, ibid.; on the remittent fever of infants, 237; observations on the fever prevalent aboard the ships of war in the Mediterranean, 246; extensive practice of depletion, 247; fatal cases of gastritis and determination to the brain, ibid.; utility of venesection defended, 248; efficacy of antimony and calomel in typhus, 322; on the heat evolved during inflammation, 323; review of tracts on delirium tremens, by T. Sutton, M.D. 333, 408; case of brain fever following intoxication, 338; case of hæmorrhœa, or petechiæ without fever, which terminated fatally, 339; on the use of purgatives in purpura, 342; on the fever attending gunshot wounds, 460; cases of puerperal fever, 515; observations on brain fever, 516; case of delirium tremens, xxxi. 40; the fever in hooping-cough resembles typhus, 60; on the effects of ammonia in preventing contagion, 83; account of a contagious fever at Gibraltar in 1813, 258; a distinct disease from the bilious remittent fever, 259; cases of fever in children treated by leeches, 363; observations on the peripneumonia typhoides as it prevails in North America, 471; practical account of the bilious remittent as it appeared in the ships and hospitals of the Mediterranean fleet, 489; depletion not to be used in all fevers, 490; general appearance on dissection, 494; on the fevers of Carthagena and Gibraltar, 495; account of an epidemic fever at Grenada and Martinico, xxxii. 45; proofs of its being contagious, 46; practice of Hippocrates in, 114; objections to the word typhus, 143; remarkable disorder after recovery from fever, 166; fever maintained by Cullen and Fordyce to be a disease of the whole system, 183; considered by others as a symptom of organic inflammation, ibid.; varieties in the mode of treatment, ibid.; prevalence of putrid fevers at Paris, 256; on an inflammatory fever which prevailed in the 24th regiment in 1813, 267; emetics injurious, 268; that fever contagious, 269; importance of bleeding in the same, 270; on the inflammatory fevers of the army, 271; efficacy of bleeding in intermittents, 345; alarming symptoms of scarlatina, 352, 353; Thoughts on Puerperal Fever, and its Cure by Spirits of Turpentine, by John Brenan, M.D. 403; benefit of charcoal in intermittents, 428; on the frequent occurrence of typhus in London, 441; severity of scarlatina, ibid.; observations on puerperal fever with diarrhœa, 461; on the fevers of women in childbed, 467; observations on the treatment of scarlatina, 481; Facts and Observations relative to the Fever commonly called Puerperal, by John Armstrong, M.D. 507; account of a remarkable epidemic in New York and New England, xxxiii. 92; the disposing cause of the disease in the atmosphere, 96; identified with the petechial fever of 1810, 97; on the depletory treatment of febrile diseases, 173; on the terebinthinate treatment of puerperal fever, 179; reply to a letter on the treatment of fever by blood-letting, addressed by the four permanent physicians of the Fever Hospital at Cork, 332; account of an epidemic fever in Cambridgeshire, 338; remarks on the efficacy of cold affusion in typhus and scarlet fever, 358; account of a putrid fever among the horned cattle in France, 401; account of an epidemic fever at Gibraltar from 1804 to 1813, 414, 428; case of puerperal peritonitis, 447; observations on chronic peritonitis, xxxiv. 11; the nervous functions affected in fracture like typhus, 66; reflections on fever, intended to point out the principles upon which a systematic and useful method of treatment might be established, 158; fevers alleged to depend upon the action of the solar light, 161; on the state of the brain in fever, 163; part of the Small-Pox Hospital applied to the use of the sick poor in fevers, 168, 283; case of fevers cured by cold affusion, 175; on the efficacy of spirits of turpentine in puerperal fever, 183; observations on the fever prevalent in the province of Guzerat, 243; on the fevers which prevail at Seringapatam, 244; on the necessity and utility of blood-letting in continued fever, 249; reports on the epidemic of Gibraltar, 301; transition from a simple epidemic fever to a contagious one, 307; a View of the Mercurial Practice in Febrile Diseases, by John Warren, M.D. 323; mercury useful in typhus, 331; the nature of febrile contagion imperfectly understood, 337; French report on the entero-mesenteric fever, 338; symptoms of the complaint, 339; appearances after death, 341; the disease consists of an inflammation of the mucous membrane of the intestines, 343; cases of inflammatory fever, 351; hospital gangrene produced by febrile exhalations, 403; reports on the pestilential disorders of Andalusia, 406, 498; puerperal fever fatal at Paris, 487; origin and progress of the fever at Cadiz, 499; on the epidemic at Gibraltar, 503; on the pestilential fever at Malaga, 506; farther account of the epidemic at Cadiz, 509; on the intermittent of Walcheren, 511.

FEVER, (Yellow,) renewal of its ravages in North America in 1797, i. 78; successfully treated with calomel, ibid.; free use of mercury recommended by American physicians, 79; account of an historical work on that subject, ibid.; account of its appearance at Philadelphia, 105; observations on the nature of it, ibid.; said to be produced by heat and deficiency of oxygenous air, 106; proceedings of the American College of Physicians on the subject, ibid.; committee at New York for inquiry into the cause of it, 199; memoirs of the yellow fever as it prevailed at Philadelphia and other parts of the United States, 206; imported from St. Domingo, 207; observations on its cause, prevention, and cure, 246; its appearance at Philadelphia, 248; on the virtues of lime, magnesia, and alkaline salts in that disorder, 274; use of bleeding as a preservative against, ii. 178; ill effects of that practice during the disorder, 179; benefit of acids in it, 181; account of the bilious malignant fever in New Hampshire, iii. 568; injury of the cold-bath in that complaint, 569; how generated in ships and jails, iv. 113; practical observations on it in the West Indies, 355; symptoms described, 357; efficacy of calomel in it, v. 133; review of an Essay on the malignant pestilential fever introduced into the West Indies from the coast of Guinea, 184; observations upon the origin of that disorder at Philadelphia, 291; prize question on it, 588; ascribed to putrid coffee, vii. 311; an injury received in making experiments on the black vomit, 312; communicated at Cadiz by an American crew, 313; on the variety of opinions concerning its origin and treatment, 314; on the use of mercury in it, 315; increased population in America notwithstanding the ravages of the fever, 320; brandy efficaciously employed in some stages of it, 571; scirrhous fever caused by it, viii. 245; erroneously denominated, ix. 26; difference of opinion on the cause and treatment, ibid.; on the vigorous use of mercury in it, 27; that practice superior to bleeding, 28; fatal to strangers in the West Indies, 29; on the deficiency of bile in the disease, 30; extensive utility of mercury, 31; advice to persons visiting climates where it is prevalent, 32; farther observations on the benefit of mercury as a prophylectic, 34, 37; its malignity in Jamaica, 37; introduced at Philadelphia from St. Domingo, 97; account of its progress there in 1802, 98; bad effects of mercury in, ibid.; various modes of inefficient treatment, 99; favourable account of the warm-bath, ibid.; utility of laudanum, 100; benefit of lime water and milk, ibid.; blisters serviceable in it, 102; regulations for the use of mercury, ibid.; correspondence between Dr. Miller and Dr. Patterson on the subject, 103; said to be not contagious, 104; that opinion controverted, 107; imported into Charlestown several times from the West Indies, 108; authorities proving it to be infectious, 109; not caused by local filth, 110; begins in the hot months, ibid.; analogy between this disorder and others of a pestilential character, 111; stagnant waters not the cause of it, 112; ascribed to dephlogisticated nitrous air, 304; account of a successful method of treating it by bleeding at the commencement, 581; account of the favourable and unfavourable symptoms, x. 37; on the black vomit, ibid.; appearances on dissection, 38; benefit to be obtained from emetics, ibid.; inefficacy of bleeding, ibid.; powerful use of mercury, ibid.; benefit of kali preparatum and lime juice, 39; utility of blisters, ibid.; enemas recommended, ibid.; advantage of quassia and the cold-bath, 40; description of the symptoms of yellow fever in Africa, 87; account of its appearance at Norfolk, in Virginia, 266; its connexion with intestinal disorders, 270; efficacy of calomel and cold affusion in it, 272; its appearance at Wilmington, in North America, 365; proved to be not contagious, 368; called the typhus of the tropics, 383; caused by noxious exhalations, 461; benefit of depletion followed by mercurial salivation proved at Charlestown, in Carolina, 471; friction with oil recommended in, xi. 480; its resemblance to the plague, xii. 79; a modification of typhus, 249; on the virtue of oily frictions in, xiii. 17; on the yellow fever at Catskill, North America, 304; xiv. 312; observations on the black vomit, xiii. 378; resuscitation in a case of supposed death from, xiv. 7; the yellow fever of America not dependant on the state of the atmosphere, 79; case of its cure by sweating in the steam of hot sugar, 185; questions proposed on that subject by the Medical College at Berlin, 381; sagacity of birds in avoiding places infected by it, 382; bark said to be a specific in that disorder, 531; spirit of turpentine recommended as a remedy, xv. 16; benefit of cold bathing in it, 17; efficacy of mercury combined with cold application, 19; denied to be contagious, 41; recantation of Dr. Rush on the contagious property of the yellow fever, 270; on its contagious nature, 560, 561; appearances of the spleen in, xvi. 195; cases of it, with dissections, in the West Indies, 268, 269; strictures on a Latin essay on yellow fever, by Dr. Grant, 410; benefit of cold affusion in this disorder proved in several instances, 490; history of the malignant fever at New York in 1805, xvii. 97; the doctrine of contagion in that case invalidated, 107; not imported thither from the West Indies, 112; its various appearances in different seasons and countries, 114; on the different opinions prevalent upon the subject, 121; analogy between the cities of Rome and New York in their diseases, 122; historical sketches of the antiquity of the yellow fever, 124; indigenous in the island of Minorca, 125; observations on it in the interior of America, 126; on the singular opinions of Dr. Chisholm, 128; prize questions by the London Medical Society on the subject, 198; on its causes, 451; three peculiar circumstances in its history, xviii. 41; on the variety of opinions concerning its origin, 42; anniversary oration delivered at Philadelphia, to disprove its contagious property, 111; its first appearance at New York, 121; letter of Sir Gilbert Blane to Baron Jacobi on the subject of yellow fever, its causes and prevention, 468; caused by bad air and other agents, xix. 113; the brain principally affected in it by congestion, 169; on cold affusion in the disorder, 175; opposite opinions on its origin, xx. 27; its first occurrence at Charlestown, South Carolina, ibid.; historical statement of its progress, ibid.; occasioned by a pernicious vapour floating in the atmosphere, 28; local peculiarities favourable to its dissemination, 29; on the laws that govern contagion, 30; prize essay by the College of Medicine at Berlin on that subject, 31; proofs of its being contagious, 32; is governed by the state of the atmosphere, 33; why Philadelphia was so long free from the disease, ibid.; causes of its occurrence at Cadiz and Gibraltar, 34; uncertainty of the contending theories on the subject, ibid.; use of the mineral acid gases in checking contagion, 35, *note*; cases of its occurrence by contagion, xxi. 77; farther evidence of its infectious nature, 241; account of the disease at Grenada, 242; its similarity to the pestilence at Athens, 243; on the pestilential fever of Bulam, 247; analysis of the different opinions on this subject, xxii. 105; efficacy of bleeding in preventing it, 228; observations on that treatment, 298, 459; zeal of the American practitioners to discountenance the idea that yellow fever is contagious, xxiv. 22; on the extravagant use of mercury in the treatment of yellow fever in the West Indies, 461; its supposed connexion with the pest, xxv. 243; on the exactness of some statements relative to the efficacy of cold affusion in this disease, 360; proofs of its not being contagious, xxvii. 36; originates in marsh miasmata, 37; report on the yellow fever at Perth Amboy, New York, in 1811, xxviii. 145; singular conduct of the faculty in that case, 147; imported from the West Indies, 148; decided proofs of its contagiousness, 149, 150; on the efficacy of cold affusion in that disorder, 296; benefit of calomel and antimony in the complaint, 298; beneficial use of purgatives in yellow fever, 447; on the vigorous use of mercury in the disease, xxix. 213; that plan exclusively adopted in America, 214; doubts of its efficacy, 215; efficacy of blood-letting in yellow fever, 412; cold affusion deprecated, 413; observations on the yellow fever of New York, 416; injury arising from the free use of mercury, ibid.; symptoms in what are called walking cases, 417; on the introduction of the depletory method of cure, xxx. 76; the carbonate of ammonia recommended, 232; its occurrence at Gibraltar, xxxi. 258; proofs of its infectious nature, and its communication by the Hankey from Grenada, xxxii. 45; other instances of its being contagious, 46; cursory observations on the treatment of yellow fever, xxxiii. 5; benefit of purgatives, 6; on the free use of venesection, ibid.; on the necessity and utility of blood letting in the ardent fever of the West Indies, xxxiv. 249; on mercury in that complaint, 325; account of the yellow fever as it appeared at Boston, 334.

FEVERFEW, *(matricaria parthenium,)* botanical description of, xix. 457; successfully given as a vermifuge, and for the cure of intermittents, ibid.; chiefly beneficial in female complaints, ibid.; an essential oil obtained from it, ibid.; description and virtues of the chamomile feverfew, 458.

FEVRE, Dr. le, a French Charlatan, account of him and his practices, xxiv. 351.

FEWSTER, Mr. his observations on the inoculated cow-pox, i. 9; on inoculating cows with grease, iv. 466; his evidence on the equine origin of the cow-pox, xvi. 128.

FIRTH, Dr. account of his dissertation on the malignant fever of North America, xiii. 378; on the use of nitric acid in hepatitis, xviii. 301.

FIBRES, (animal,) on the irritability of the muscular and nervous, i. 53; those of aged persons liable to break, 63; on the want of cohesion in the, 157; action of the muscular, ii. 163; on the excitability of, 309; effects of acetic acid on the, iii. 88; method of treating the debilitated, v. 471; effect of galvanism on the muscular, 587; observations on the membranous, vi. 452; destructive effects of carbonic gas on them, 496; characteristics of the muscular, viii. 370; on their irritability, ibid.; effects of opium on the muscular, ix. 51, 341; galvanic effects on the muscular, 147; effects of phosphorated ether in atony of the, 300; animal fibre sensible of heat, x. 25; on the particular structure of the fibres of the human body, 26; experiments on the fibre of the frog, 27; loses its electricity by repetition, 28; action of oxygen on the animal, 29; experiments on the irritable muscular and nervous, 55; the animal fibre composed of glutinous matter, 252; account of the fibrous structure of the arteries, xv. 464; on the fibrous matter of the blood, xvi. 303; observations on the fibrous structure of muscles, 519; effects of cold on the muscular, xvii. 92; muscular fibres become soon putrid by galvanism, 190; on those which form the obicular muscle of the iris, 406; concerning the action of medicine on the living, xix. 365; observations on the action of fire on the living, xxi. 386; the muscular fibre not the subject of phosphorescence, xxiii. 391; the radiated ones forming the muscular coats of the bladder, xxvii. 381.

FIBRES, (vegetable,) description of them, i. 378; the same analysed, v. 467; no true ones to be found in vegetables, vii. 557; linen made from those of the bark of flax, xiv. 63.

FIBRINE, the constituent part of the solids of the body how formed, viii. 297; effect of galvanism on, ix. 291.

FIBULA, case of fracture of the, xviii. 309; case of imperforate, 346.

FICHTE, Professor, on the nourishment of plants, v. 370.

FIEDLER, Mr. obtains acetous ether from acetate of lead, iii. 268; his method of preparing gallic acid, xii. 382.

FIELD, Mr. his observations on the influenza at Worcester, x. 223; reply to his justification of the Apothecaries' Company in regard to the London Pharmacopœia, xxx. 79.

———, George, description of his improved stove for heating rooms, xvii. 583.

———, Henry, his successful cases of cynanche trachealis, with the method of treatment, ii. 146; observations on his cases, 156; his case of intestinal ulceration, with dissection and remarks, xv. 386; his report of the diseases at Christ's Hospital, xxxi. 479; account of a fatal case of enteritis, 481; on the cynanche trachealis, 482; instances of small-pox after inoculation, 484.

FIELDHOUSE, Mr. his account of the influenza at Stafford, x. 399.

FIELDING, George, on the beneficial effects of digitalis in hooping-cough, v. 141; retains the merit of having first recommended antimony in ophthalmia, xxix. 452; reply to his claim, xxx. 46, 199; answer to his observations on the efficacy of antimon. tart. in acute ophthalmia, xxxi. 17; on his claim to priority in that practice, 189.

FIFE, Mr. his observations on the inflammation attending vaccine inoculation, xiii. 63.

FILAGO, or childing cudweed, botanical description of, xix. 539.

FILAMENT, how it is united to the flower, i. 36; description of the vegetable, ii. 243; on the tenuity of the muscular, xvi. 519; history of injury done to the nervous filament of the fore-arm, xvii. 188.

FILICES, description of that order of plants, xix. 541; observed in a fossil state, 542.

FILTRATION, description of machines invented for purifying oil and water, i. 86; specification of a patent for cleansing all kinds of fluids, 402; porous vessels of earth used in Spain for that purpose, iii. 174; method of purifying water at Paris, vii. 477; charcoal beneficial for purifying water, xii. 96; description of a new apparatus for that purpose, ibid; simple process for purifying water, xiv. 191; mechanical method of filtering water without sand or charcoal, xxii. 439.

FINCH, Rev. Mr., his successful practice of vaccine inoculation, iii. 415.

FINCHAM, Mr. his case of disordered alimentary canal owing to the swallowing of a skein of cotton, xxiii. 431.

FINGERS, arthritic concretions taken from them, i. 194; case of tetanus succeeding a wound in the, ii. 481; gouty concretions of them analysed, iii. 174; cases of venereal infection taken by the, 198, 201; case of paralysis of the hand and fingers, with a separation of the metacarpal bones, 253; observations on that case, 254; case of incipient mortification of the fingers by immersing the hand in cold water, v. 534; case of a child born with the fingers united and terminating in knobs, xii. 198; on the appearance of gout in the, xv. 178; suppuration in the finger joints, 180; how affected by the bite of snakes, xxiv. 400; cases of fingers restored and re-united after being totally separated, xxxii. 411, 413; on some diseases of the, xxxiii. 151; case of re-union of the first phalanx of the middle finger, xxxiv. 248; other instances of a similar nature, 515, 516.

FINLAND, substitute for bread adopted there in time of scarcity, xv. 267; the prunus used by the inhabitants for syphilitic complaints, xvi. 76.

FINSBURY DISPENSARY, reports of diseases under the care of the physicians at that establishment in the year 1800, iii. 391, 409, 506; iv. 14, 105, 202, 298, 393, 490; v. 97; the same for 1801, v. 194, 298, 392, 413; advice to the poor from that institution on the efficacy of vaccine inoculation, ix. 274; cases of scarlatina successfully treated by cold ablution, in the, xi. 27; account of cases there in 1805, xiii. 375, 476, 567; xiv. 14, 183, 275, 378, 476, 569; xv. 90, 193, 291; xxv. 178.

FINSBURY DISPENSARY, reports of surgical cases in the same charity, viii. 108, 204, 300, 398, 493; ix. 13, 188, 272, 373, 467, 568; x. 74, 178, 275, 376.

——————, election of a surgeon for that institution, xiv. 384; indiscriminate inoculation practised there, xxiv. 148; address to the subscribers and directors of that institution on this gross abuse of the charity, xxv. 459.

FIRE, phlogiston said to be combined with, i. 65; new theory of, founded on experiments and analysis, 109; decomposes water, 195; on the means of lessening its effects on the human body, iii. 191; utility of spirit of turpentine in accidents from, 262; benefit of cold in such cases, ibid.; that opinion controverted, 207; antiphlogistic doctrine concerning, 271; observations on human combustion, iv. 44; attempt to refute some opinions on the efficacy of ice in accidents from, 454; benefit of spirits of turpentine in a case of burn, v. 55; confirmation of the virtue of turpentine in accidents by, 236; ineffectual for purifying an infected atmosphere, viii. 535; its action upon leather, x. 25; improvement in the construction of fire-places, xi. 100; how to be employed in preventing contagious diseases, and purifying the air, 109; case of the efficacy of spirits of turpentine and laudanum in, xiii. 14; observations on the violence of subterraneous fire, 108; new composition for preserving inflammable bodies from, 288; patients immersed in liquid fire saved by the terebinthinate treatment, xvi. 8; that opinion disputed, 210; impure air renovated by burning bodies, xvii. 449; formula of a composition that will resist the action of fire and water, xix. 479; description of a fire alarm, 575; the great water-moss used by the Scandinavians to prevent their houses against, xx. 159; how produced by the compression of atmospheric air, 287; observations relative to the action of fire on the living fibre, xxi. 386; improvement in the construction of fire-places, xxiii. 152; history of three persons who were nearly suffocated by the gases arising from a coal fire, xxvi. 162; on spontaneous combustion in manufactories, xxviii. 514; on that produced by drinking spirituous liquors, 515; efficacy of cotton externally applied in a case of burn, xxix. 137, 140; account of compositions invented to extinguish fires, 493; description of Godfrey's invention for that purpose, 494; instances of combustion during the combination of several chemical substances, xxx. 523; extraordinary account of the spontaneous combustion of a woman from drinking spirits, xxxii. 164; another case, xxxiii. 425.

FIRKMAN, Dr. his singular case of urinous vomiting, xxix. 363.

FISCHER, J. C. his observations on the air bladder of fishes, ii. 360; his method of preparing the red oxyd of mercury, viii. 573.

————, Gotthölf, on the different forms of the os inter maxillare in animals, v. 566; review of his Fragments on Natural History, vii. 372.

FISHER, S. his case of compound fracture of the thigh, xi. 37.

————, James, a fictitious name, under which appeared an empirical tract on the virtues of stramonium, xxv. 66; detection of the imposture, xxvi. 151.

FISHES, process for purifying the oil of, i. 86; fish diet improper for gouty patients, 150; analysis of La Cepede's natural history of, 203; adapted to particular climates, 405; on the respiration of them, ii. 79; the effect of eating fish in diabetes, 86; the effect of eating them on the urine, 88; description of their organs of generation, 244; observations on their migration, 245; on the fecundation of them, 322; experimental observations on the air bladders of, 360; on the hearing faculty of whales, 480; on their nutriment as food, iii 69; that diet the cause of cutaneous diseases, 153; observations on those nations among whom fish constitutes the principal article of food, 252; on the cellular structure of their bones, iv. 274; on the nerves of electrical ones, 335; their acute sense of smelling vi. 341; on the musky odour of some, 345; on the minute divisions in their blood-vessels, vii. 373; ossification never complete in large ones, viii. 371; description of the organs of the cuttle-fish, 372; a fossil one found in a quarry near Paris, x. 480; on the poison of muscles, 483; herpetic eruptions common to those who live upon fish, xi. 240; intoxicated by the seeds of the verbascum, xii 131; fish diet hurtful to gouty persons, 158; have a mode of respiration peculiar to themselves, xiii 371; description of their organs, ibid.; effect upon water, ibid.; on the organic construction of, 430; on the power and particular structure of the muscles of, xv. 95; discovery of fossil ones, 391; the eudiometers of water, xvii. 197; description of the curbinata, in the head of which are two stones of extraordinary virtue in retention of urine, xx. 287; feed upon fuci, 345; experimental observations on the poison of fishes in the West Indies, 461, aversion of the Dutch to mackarel, 462; enumeration of those deemed poisonous, 463; how persons are affected by eating them, 464; copper supposed to be the basis of the poison, 465; how detected, 466; ascribed to the manchineel apple, ibid.; on the phosphorescence of, xxiii. 369, 387; experiments on their respiration, 451; suffer when placed in water freed of air, 452; on the swimming bladder in, 453; on the vertebræ of the shark, xxiv. 5; experiments on the gymnotus electricus, 38; instances of poison from eating, xxv. 398; supposed to arise from copper, 399; ascribed to the influence of the moon at St. Helena, ibid.; description of the albicore, ibid.; experimental observations on luminous ones, 409, 512; description of the Montee of Normandy, xxvii. 377; on various kinds of luminous, ibid.; the trichanus lepturus found on the coast of Scotland, xxix. 429; on the temperature of, 443; the squalus maximus, or great shark, described, xxx. 250; cetaceous ones thrown on the coast of France, 432; small eels found in French rivers, 433; researches into the structure of the mouths of, xxxiii. 398; observations on different kinds collected in the Mediterranean, 399; on the shells of, xxxiv. 165

FISSURE, no motive of itself for trepanning the skull, iii. 33; successful case of that of the cranium, attended with apparent exhaustion, x. 244.

FISTULA, benefit of garden snails in fistulous ulcers, ii 75; case of one in the stomach, in which that viscus could be seen through it, viii. 361; appearances on dissection, 363; remedy for fistulous sinuses, xv. 324; case of fistulous ulcers in the back terminating in apoplexy, xvii. 422.

FISTULA IN ANO, danger of attempting its cure, ii. 154; case of one accompanied with the lodgement of a plum-stone in the rectum, iii. 224; description of a new instrument for operating in it, with a plate, 493; on the little attention that has been paid to it by English medical writers, xxiv. 421; on the hæmorrhage attending the operation for, 422; the decoction of the prunus sylvestris a remedy in, xxvii. 75; observations on the treatment of that complaint, xxxiii. 237.

FISTULA LACHRYMALIS, method of treating it by Fallopius, ii. 280; benefit of alkalies in, iii. 168; case where an operation was necessary after topical bleeding and all other means were ineffectual, xi. 551; distinction observed between the pus which appears at the puncta lachrymalia and the true fistula, xvi. 563; objections to the common mode of operating for it, xvii. 283; account of a case, with reflections on the different modes of operating in the disease, xx. 147, 150; only two modes of giving relief, ibid.; tumour of the lachrymal sac not properly a fistula, 152; description of the disorder, xxvi. 230; on the enlargement of the cautery in, 233.

FISTULA IN PERITONEO, case of diseased bladder attended with, xvi. 141.

FITS, account of an empirical powder for the cure of, i. 179; the lycoperden a specific in, 180; efficacy of the misletoe in, ibid.; nitrate of silver beneficial in, 181; farther proofs of its efficacy, 222; benefit of opiate frictions in, 42; case of the virtue of argentum nitratum in, ii. 70; another of the same, 173; case of their occurrence after an injury of the head, iii. 31; bleeding apt to bring them on in children, 57; fatal case of, 325; efficacy of alkali in, iv. 183; opium externally applied with success in a case of, 570; violent hysteria cured by musk, v. 235; cured by electricity, 348; inefficacy of the nitrate of silver in, 436; use of alkalis in convulsive affections, 471, 557; how administered in slight cases, 475; on the use of nitrate of silver in, 503; phosphoric acid recommended in, vi. 92; on those of pregnant women, 280; their connexion with the nervous system, 377; case of combined catalepsy and epilepsy, 425; relieved by vermifuges, 426; another case occasioned by worms, 428; case of catalepsy, vii. 30; another of the same cured by bark, 105; singular case of laughing fits, 106; effects of galvanism in, viii. 321; the datura stramonium beneficial in, 427; case of epilepsy cured by applying caustic to the nerve, ix. 51; benefit of cold water in, x. 411; flowers of the Galium Urum used as a remedy for, xi. 533, 534; on the supposed benefit of the viscus quernus in, xii. 33; dose used by Colbech, ibid.; the leaves of the box said to be beneficial in, 37; the datura stramonium serviceable in, 131; hyoscyamus effectual in epileptic fits, 132; solanum nigrum administered in, 136; the viola tricolor said to possess great virtue in convulsive affections, 204; efficacy of cold water in convulsions, 508; aggravated by parsley, 558; case of catalepsy, with remarks, xiii. 180; efficacy of sugar of lead in epileptic cases, xiv. 10; case of epileptic fits occasioned by anger, 275; on the efficacy of sedum acre in, 430; galvanism a cure for, 527; case of the benefit of cold water in convulsions, xv. 52; benefit of oxygen gas in, 582; epilepsy cured by trepanning, xvi. 473; transposition of the senses occasioned by, 525; utility of penny-royal in, xvii. 501; case of convulsions, 548; the misletoe a popular remedy among the ancients for, xviii. 16; carbonate of potash and opium administered with success in, 94; the cardamime said to be efficacious in convulsive affections, 273; case of epilepsy cured by sugar of lead, 353; case of fits occasioned by worms, xix. 305; hysteric fits cured by the leaves of artimesia or southern-wood, 358; remarkable case of fits produced by the bite of a spider, xx. 224; affected by music, 229; hereditary in some families, xxi. 339; case of epilepsy, in which the recurrence of the paroxysm was prevented by cold affusion, xxiii. 20; powerful effects of stannum in different kinds of fits, 316; on convulsion as a consequence of disease, illustrated by cases, 369, 480; xxiv. 52; violent asthmatic ones occasioned by ipecacuanha, xxiv. 233; case of hysteria from belladonna, 383; remarkable instance of epileptic affections in a female, xxv. 179; efficacy of camphor externally applied in, xxvi. 316; remarkable history of somnambulism, 483; on the physiognomic characteristics of epileptics, xxvii. 40; history of a case of periodic catalepsy, xxviii. 169; observations on that case, 376; benefit of nitrate of silver in convulsions, xxxi. 148; fits produced by a spider in the ear, 172; produced by methodistical preaching, xxxi. 373; answer to the case, 464; vindication of the same, xxxii. 32; cases of periodical jactitation, xxxiii. 60; state of the cerebellum in epileptics, 182; efficacy of nitras argenti combined with the shower-bath in, 273; singular case of chorea, 431; charms used for the cure of, xxxiv. 85; singular case of fits in a boy, 263; remarks on the treatment of, 517.

FITZPATRICK, Dr. Thomas, remarkable case of abscess in the groin which ended fatally, xv. 221; on the occasional cessation and return of epilepsy, xxxii. 293.

FLACKSLAND, Dr. his extraordinary instance of a monstrous fœtus, vi. 94.

FLANNEL, on the advantages and disadvantages of wearing it next the skin, i. 39; hurtful to perspirable constitutions, 41; utility of flannel rollers in wounds and ulcers, ii. 91; its use recommended in hot climates, x. 84; flannel bandages serviceable in dysentery and diarrhœa, 281; on the flannel dresses of children, xi. 470; beneficial in gout, xiii. 562; and in rheumatism, xvi. 496.

FLAP, account of that operation in cases of amputated limbs, xiii. 98.

FLATULENCY, case of flatulent colic, with hernia, xiv. 271; case of it with the regurgitation of food, xxiv. 248; occasioned by the extinction of air from substances in the stomach, 249; necessity of attention to diet in, 250; utility of emetics, 251.

FLAUGERGUES, M. his account of luminous earth-worms, xxv. 410.

FLAVOUR, too often confounded with taste, xxviii. 457; definition of taste, ibid.; flavour may exist without taste, 459; the sensation defined, 460; the seat of flavour more extensive than that of taste, ibid.; the former an intermediate sensation, partaking of taste and smell, ibid.

FLAVUS, objection to the application of that word in the Pharmacopœia Londinensis, xi. 301; the term vindicated, 537.

FLAX, *(linus,)* botanical description of, xiv. 62; a native of Egypt, ibid.; virtues of linseed, ibid.; produce flatulency, 63; oil-cake given to oxen, ibid.; description of *Linus Antharticus*, or purging flax, ibid.; whether the steeping of it be injurious to health, xvi. 101; used by Hippocrates as a cautery, xix. 358; method of preventing its destruction by the caterpillar, xx. 384; prize offered in France for the best machine to spin, xxv. 545.

FLEA, curious remarks on the water, vii. 191; branches of the myrica, or Dutch myrtle, used to keep off fleas and moths, xii. 36.

FLEET, account of a contagious malignant ulcer in the English, xxiv. 13; plan for improving the medical department in the, xxv. 443.

FLEMING, Rev. John, his description of the Narchal, or Sea-Unicorn, xxi. 171; account of the old silver mines in Linlithgowshire, xxix. 430.

FLEMING, Dr. his valuable acquisition of Indian medicinal plants, xxvi. 30.

FLEMYONG, Dr. his observations on apoplexy, viii. 86.

FLESH, that of animals only preserved from putrefaction by soda, ii. 470; the invigorating quality of raw flesh as an article of food, iii. 67; all flesh of the same kind of animals not equally nutritive, ibid.; effects of beer in restoring putrefied, 90; account of a man who lived upon large quantities of raw, 209; experimental observations on the putrefaction of it in different gases, ix. 219; potash recommended to be used with it as food, x. 475; means of preserving animal substances from putrefaction, xi. 358; instances of bones changed into, xvi. 451; that of a human body converted into fat, xxxi. 45; experiments on the preservation of various kinds of animal flesh in gases, xxxii. 435.

FLEICHER, Dr. G. on the cause of jaundice, vi. 312.

———, Edward Baynes, his case of obstinate gleet cured by the use of sea-water, xxxiv. 247; his cases of the reproduction and re-union of part of the finger after accidents, 515.

FLIES, receipt for keeping them out of apartments and stables, xvi. 190; the anterrhinum or toad-flax a poison to, xviii. 171; the bug agaric effectual in expelling them, xx. 353; observations on a peculiar genus of acarus, xxi. 8; on the vesicating properties of the potatoe-fly, xxiii. 323; natural history of that insect, 324; discovery of a new blistering one in India, xxiv. 430; description of plants that have the property of catching and retaining, xxvii. 388; frozen ones recovered, 486.

FLOODING, on its occurrence in the seventh month, iii. 478; how to be checked, 479; case of it between the seventh and eighth months, occasioned by the attachment of the placenta to the mouth of the womb, xxi. 424.

FLOODS, mechanical contrivance for discharging superfluous waters from ponds in times of, xx. 383.

FLOOKBOROUGH, Lancashire, mineral spring discovered there, xxv. 84.

FLORA, see *Plants*.

FLORENCE, introduction of vaccination at, xvii. 63; state of the medical schools and hospitals at, xxxi. 82.

FLORES, Dr. Joseph, his specific for cancer found in New Spain, xxvi. 26.

FLORIDA, description of a beautiful shrub, called the fly-catcher, in, xxvii. 391; bilious intermittents cured there by the empatorium perfoliatum, xxviii. 476.

FLOUR, its efficacy when applied hot in erysipelatous and cutaneous eruptions, i 84; farther account of its virtues, 152; great proportion of phosphat of lime contained in, iii. 89.

FLOWERS, how the filaments are united to, i. 36; irritability of those of the sorrel thorn, ii. 80; method of preserving their natural colour in a dried state, iii. 91; mode of preparing martial, vi. 468; a valuable oil extracted from the sun-flower, xx. 385; description of those which have the property of catching and retaining insects, xxvii. 388; on the changes which the eggs and larvæ of insects effect upon those of the armice montena, 406; on the ascent of sap in, xxx. 251; obscurity in regard to the characters of the female, 253; on the organization of the male flower of mosses, 254.

FLOYER, Sir John, his observations on cold bathing, iv. 398; account of his treatise on the virtues of cold water, viii. 443; on the causes of dyspepsia, xxiv. 388.

FLUIDS, on their succession in the animal system, i. 489; on the fluidity of sulphur, 502; all known ones except air and oil contain electricity, ii. 9; on the affinities of pestilential fluids to other bodies, 180; application of the doctrine of septic fluids to explain diseases of the teeth, 380, 464; on the elastic fluidity of melted snow, iii. 90; on the perspirable fluids of the human body, iv. 9, 109; on the doctrine of the fluid nature of the animal spirits, 334; of inoculating with purulent fluids, 488; on the enlarged use of the word in medicine, 557; on the hypothesis to account for the elasticity of æriform ones, v. 550; on the exhaling fluid of the natural body, vi. 137; researches and discoveries on the nervous fluid, 175; on the elastic ones of different marshes, vii. 121; method of evacuating fluids under the scull, 216; identity of the electric and nervous fluids, 324; experiments to ascertain whether they are conductors of caloric, 400; on the inutility of evacuating morbid ones in some cases, viii. 13; heat expedites the putrefaction of animal fluids, 205; galvanic fluid easy to be decomposed, 317; on the electrical phenomena which do not depend on the theory of two fluids, 477; on ascertaining the duration of the electric fluid, 572; atmospheric air resists negative more than positive electricity, 573; quantity discharged in a case of ascites, ix. 212; those of the human body have different states of electricity, x. 27; identity of the electric and galvanic, 28; are bad conductors of heat, 252; indigestion caused by want of gastric fluid, 319; experiments with the Voltaic pile on the animal fluids, xi. 287; red currants correct the putrid and scorbutic state of the animal, xii. 225; essay on the analysis of the animal fluids, with the view of ascertaining their definite characters, 263; farther experiments on the analysis of animal fluids, xv. 280; effect of the galvanic upon vegetables, xvi. 479; on the doctrine of pestilential fluids as the cause of fevers, xviii. 41; analysis of some of the principal fluids in the human system, xix. 124; that the friction of fluids does not excite sensible heat, xx. 97; on the poisonous fluids of the viper and insects of Great Britain, xxi. 7; on those necessary to the process of digestion, xxii. 6; on the property of cold in condensing, xxiv. 70; on the different kinds of fluids considered as pus, xxv. 326; on collections of watery fluid in tunica vaginalis, 353; why difficult of digestion, xxvi. 275; on the lymphatic fluid ascribed to the serous membranes, xxvii. 357; chemical analysis of dropsical fluids, xxviii. 62; on the animal matter combined in them, ibid.; tabular view of the proportions of saline and animal matter in dropsical fluids and the serum of the blood, 63; the pericardium supposed to contain an unusual quantity of fluid in apoplexy, 213; chemical researches on some of the animal fluids, 386; on the composition of chyle, 387; analysis of lympta, 390; on the serum of the blood, 391; on the coagulum of blood, 393; on the colouring matter of the blood, 394; effect of acids on the same, 395; effect of alkalies, 397; general results of these inquiries into the animal fluids, 400; on the equilibrium of liquids and solids, 415; on the gravity and levity of liquids, 416; inquiry into the structure of fluids, and the cause of fluidity in general, 433; the atoms of fluids between the limits of attraction and repulsion, 434; the change of quantity and quality in fluidity, 436; the great distinction between a solid and fluid, 437; on the lymph in the ventricles of the brain, 472; on water considered as a fluid, xxix. 17; on the variations of fluidity in water, 18; the accumulation of fluids not always removed by the absorbents, 358; explanation of some electrical phenomena by an elastic fluid, 406; general view of the composition of animal fluids, xxx. 8; on the secreted fluids, 10; analysis of the bile, 12; mercury the only opake one, 90; a fluid supposed to be exhaled from the lamina of the cornea, 93; farther view of the composition of animal fluids, 233; analysis of urine, 234; on the electric fluid, 249; on the nature and analysis of animal fluids, xxxi. 500; new analysis of the animal fluids, xxxii. 222; the temperature of solids more easily affected by heat than fluids, xxxiii. 240; on the fluidity of the blood, 276; those separated from the blood by nervous and arterial action divided into three classes, 355; the nervous fluid a medium between the mind and body, 453.

FLUOR ALBUS, efficacy of the fabæ pichurim in, vi. 376; Dr. Mead's remedy for the, x. 48; case of malignant, xi. 144; discharges in children similar to, 453; benefit of the common camfray in, xii. 125; elm bark a remedy for, 230; benefit of the fermentilla in, xvi. 266; benefit of cantharides in, xix. 528; the dry leaves of the lamium album given with success in, xxiii. 501; case of the benefit of cantharides in, xxiv. 277.

FLUOR SPAR, on the composition of, and on its acid basis, xxx. 425.

FLUORIC ACID, experiments ascertaining its presence in the enamel of the teeth, xvi. 95; discovered in a new-found fossil, 191; detail of experiments made in France to decompose it, xxiii. 292; vapours of considerable density evolved in the process, ibid.; diminution of the gas, ibid.; affinity of the vapours for water, 293; effects of that combination, 294; action of fluoric acid upon air and vegetables, 295; a perfect acid, ibid.; detonation produced by its contact with the metal obtained from potash, 296; effects of the combustion, 299; its existence in natural springs, xxvii. 95; experiments on silicated fluoric gas, and fluoboric gas, xxviii. 157; historical sketch of the attempts to decompose it, xxx. 425; on the violent action of fluorine, 426; farther experiments on fluorine, xxxi. 337.

FLUX, occasioned in the East Indies by drinking teddy, i. 353; case of fluxus cæliacus, xii. 18; the plantago recommended as an effectual remedy in, 30; the Barberry (*bubius vulgaris.*) beneficial in bilious fluxes, xiv. 69; the cinquefoil good in the, xvi. 265; and the fermentilla, 266; the prunella vulgaris a popular remedy in bloody flux, xviii. 169; use of the geum urbanum in, 436; the bloody flux cured by elecampane, xix. 456; white maiden-hair restrains alvine fluxes, 544.—See *Dysentery.*

FODERE, F. E. review of his Treatise on Bronchocele and Cretinism, v. 175.

FŒTUS, an attempt to demonstrate geometrically the mechanism of its evolution, i. 193; account of a remarkable one enveloped in its membranes, 399; singular account of a deformed one, 224; remarks on that history, with others of a similar nature; 451; the rudiments of the fœtus formerly supposed to exist in the male semen, ii. 152; that hypothesis exploded, 153; observations on the growth of the fœtus, ibid.; remarkable case of extra-uterine, 262; on the formation of it in the womb, 324; the circulation of the blood in the human fœtus carefully examined in the sixteenth century, 371; account of one six months old with only one eye in the forehead, 479; another, in which the brain formed a hernia across the cranium, 480; another which had neither head nor heart, ibid.; a remarkable one at Paris, ibid.; account of an extra-uterine one, 481; the evolution of the fœtus affected by the action of the uterus where the arm and penis presented, iii. 5; one born without abdominal muscles, and the viscera exposed, 137; another with an extraordinary appearance on the head, 138; on the connexion of the infantile part of the placenta with the maternal, 160; erroneous opinions of the ancients on the liquor amnii, ibid.; the opinion of the Greeks and others on the allantois, 161; on the urine of the human, ibid.; description of an extraordinary one where the placenta was attached to the head, with an engraved representation, 397; one born without an anus, 496; appendix to a remarkable case of malformation in one, 497; observations on the effects of the mind in producing such deformities, 499; the larger proportion of monstrous fœtuses are females, 500; on the effects of variolous infection on the fœtus in utero, iv. 104; account of one that was defective in the brain and spine, with a plate, 189; wisdom of Providence in regard to the fœtus in utero, 191; sensation not necessary to it in that state, ibid.; one born without a head, 336; on a peculiar sort of tumour observed in the, 445; account of the graphical work of Soemmering on the various figures of the fœtus, 554; progress of increase in the embryo, 555; on the growth of the fœtus, and its gradations according to the stages of gestation, v. 44; the diameter of the fœtal head reduced by instruments in delivery, 45; that it is merely passive in its progress through the pelvi, ibid.; on premature delivery, and when to be performed, 46; how the Cæsarean operation should be performed, 52; on the causes of fœtal deformity, 170; curious instance of monstrosity, vi. 94; description of one in a case of extra-uterine conception, 362; account of one without a brain, with a plate, vii. 385; on preternatural presentations of the fœtus, viii. 213; method of delivery in such cases, 215; remarkable cases, 216; on the spontaneous evolution of the fœtus, 217; the difficulty of reducing the hand or feet proportioned to the action of the uterus, 220; account of one without arms or legs, 382; observations on the arm presentation, 394; account of a female one born without limbs, ix. 63; memoir on the nutrition of the fœtus, 255; on the origin of the caseous matter covering its skin, 257; attempt to prove that it draws its nutriment from the liquor amnii, 258; on the secretions of the placenta, ibid.; the vesicula umbelicalis serviceable to the nutrition of the embryo, 259; its destruction in cases of distorted pelvis sometimes unavoidable, 407; account of the particular conformation of one which led to some difficulty to determine its position in utero, 461; a female one with an uncommon tumour, x. 172; remains of one expelled by nature in a case of extra-uterine conception, xi. 295; case of remarkable conformation of the genital organs in a female one, xiii 179; observations on a case of malformation, 429; one born without a bladder, 575; one found in the body of a youth fourteen years old, xiv. 157; one contained in the urinary bladder fifteen years, 519; inquiries respecting the manner in which nourishment is conveyed to the fœtus, xv 229; on the causes of deformity in the fœtus, 249; not produced by the imagination, 250; on its formation in the womb, 271; one found in the abdomen of a woman eighty-three years of age, 276; remarks on the case, 277; on the causes why the fœtus resists putrefaction, 278; remarkable case of an ossified one in a woman sixty years old, 279; fatal case of extra-uterine fœtus, 385; description of a remarkable one in an early stage, xvi. 54; observations on extra-uterine cases, 385; case of small-pox occurring in the fœtus, xvii. 470; observations on superfœtation, with instances, 489; description of one without a brain, 535; cases of small-pox in the fœtus, xviii. 177; great degree of rigidity necessary for it in the uterus, 547; account of one in a case of superfœtation, xxii. 48; description of a new instrument for the extraction of it, with a plate, 103; case of one found in the abdomen of a boy, 166; two cases of small-pox communicated to the fœtus in utero, 242; observations on a case of superfœtation, 293; description of a monstrous one, in which all the viscera were double, xxiii. 84; remarkable case of the same description, 278; causes of superfœtation, 280; dissection of the fœtus found in the abdomen of a boy, xxiv. 7; singular case of monstrosity, 61; on the causes of superfœtation, 127; another case of the same, 232; one expelled per anum, 412; one born with hydrocephalus internus, xxv. 206; singular case of derangement in the structure of one, 279; a singular and complicated case of mal-conformation of the genital organs in one, 441; one contained in the body of a woman fifty-two years, xxvi. 167; case of one expelled by disease of the placenta, xxvii. 168; anatomical descriptions of the fœtus in utero, 346; on ascertaining the first motion of the child in the womb, 443, 446; observations on its sensation, xxviii. 41; queries on the earliest period of gestation when a child may be born alive, 432; a full-grown one extracted from the left ovarium, after a woman had been fifteen months pregnant, 516; account of one in a state of abdominal pregnancy, xxx. 314; one injured without the mother being affected, 510; remarkable case of superfœtation, xxxi. 150; on monstrosity in the human species, 338; account of one taken from the abdomen of a boy sixteen years of age, xxxii. 81; on the circulation of the blood in the fœtus, 168; signs of an immature child, 330; observations on monsters, 331; on the time when a fœtus begins to live, 333; description of one with a tumour as large as the head, 434; another whose umbilical cord formed a considerable sac, ibid.; one in a case of extra-uterine pregnancy, 461; proofs that the fœtus breathes the liquor amnii, 521; farther account of an ossified one retained in a woman fifty-two years, xxxiii. 65, 147; one born without a heart, 72; another retained till a second pregnancy, which ended in the death of the mother, 179; on the destruction of the fœtus in utero, 181; one born without a brain, 151; observations on such cases, 152; the imagination has no effect in them, 155; on the fœtal liver, 224; case in which a mass resembling a placenta without a fœtus was discharged from the womb, 333; another case of hydatids, and a placenta discharged without any fœtus being discovered, 346; observations on the envelopes of the fœtus, 396; the remains of one found in the abdomen of a child, xxxiv. 80; account of one born without a brain, 104; full statement of the case of a fœtus found in the abdomen of a young man at Sherborne, 317.

FOG, an uncommon one, attended with almost total darkness, in London, xxvii. 175; another in the winter of 1813, xxxi. 175.

FOGO, A. on the delivery of the placenta, xi. 63; his remarks on a case of phthisis, 147; reply to him on the delivery of the placenta, 328; another answer to him on the same, 456; his animadversions on a case of gastrodymia, xiii. 207; his remarks on certain theories of hydrocephalus, xxiii. 25; on the degree of importance attached to the functions of the uterus in regard to health, 518; on the management of twin cases, xxv. 480; additional observations on the same in reference to a particular case, xxvi. 115; answer to his remarks on twin cases, 282; farther observations on his opinions, xxvii. 109; vindication of his practice, 367; his animadversions on the history of a case of chronic hepatitis, xxviii. 95; general remarks by him on that complaint, and the mode of treatment, 99; reply to him on twin cases, 104; his remarks on a case of hepatitis refuted, 279.

FOMES, an inflammable quality in bodies, iv. 112; cases of fevers from fomites, vii. 339.

FOMITES, a cause of epidemic opthalmia, xviii. 13; how infection is conveyed by, 88.

FONTAINE, M. de la, his inquiries into the causes of the plica polonica, xxiii. 204; analysis of his treatise on that subject, with numerous cases, xxiv. 229; his opinions contested, xxvi. 388.

FONTANA, experiments made with his eodiometer, ii. 387; his experiments on atmospheric air, vi. 205; experiments with opium on the hearts of frogs, vii. 349; his opinion on the effects of opium on the nervous system, viii. 333; his work on poisons commented, xi. 336; on the benefit of tartar emetic, 337; on the necessary cruelty of his experiments, xxvii. 7; on the accuracy of his eudiometer, xxix. 408; additions to his cabinet of anatomical preparations, xxxi. 82; on the cause of convulsions by opium, xxxii. 276.

FONTANEL, case in which the dura mater was successfully punctured under the, viii. 505.

FONTANUS, Jacobus, his extraordinary history of a boy, the sutures of whose skull were divided, xxxi. 69.

FONTINALIS, or great water-moss, description of the, xx. 159; used to defend chimneys against fire, ibid.

FONTINELLE, M. account of his table of the different systems of botany, i. 204.

FOOD, effects of abstaining from it in the cure of diseases, i. 43; on a dietetic regimen, 106; advice in regard to it for hypochondrias, 149; what kinds are most proper for aged persons, 494; on that of nocturnal birds of prey, ii. 79; attention to it requisite in diabetes, 88; necessity of sufficiently masticating it, 142; effects produced by the same food on different persons, 325; effects of soda in the concoction of food, 383; necessity of a sound state of digestion, 494; on the aliments produced by the different classes of the animal kingdom, iii. 65; invigorating quality of raw flesh, 67; on the various kinds of animal food, 153; fish diet the cause of cutaneous complaints, ibid.; on the nutriment afforded by insects, 155; account of a man who lived upon raw flesh, 209; farther remarks on the different kinds of animal food, 249; the diet should be regulated by climate, 250; on the various sorts of food used by different nations, 251; effects of eating shell-fish, 252; on the action of food, and its influence upon animal life, 283; animal food proper for consumptive persons, 313; on that of insects, 376; case of pretended abstinence from, iv. 86, 187; dangerous effect of excessive fasting from, 148; substitutes for foreign articles of food, v. 371; an attention to it necessary for the cure of tape-worm, 398; account of a practical treatise on diet, vi. 178; detection of an imposture in a case of pretended abstinence from, vii. 190; disadvantages of animal food alone in certain stages of the venereal disease, viii. 12; some practitioners too particular in regard to directions on, 180; proper food for hepatic complaints, 223; purity of that of the Icelanders, ix. 22; bread used in Scotland and Wales, 24; causes of hunger and thirst, x. 20; want of appetite occasioned by defect in the gastric fluid 319; potash employed in cooking animal food, 472; the orchis a nutritious article, xi. 442; the cow-parsnip made into a delicacy by the Russians, xii. 364; pork predisposes to mortification, 484; simple aliment of the Highlanders in Scotland, xiii. 31; on that adopted by persons engaged in athletic exercises, 534; linseed used as food, xiv. 62; garlic used in soup, 65; ornithogalum luteum, or star of Bethlehem, used as a food in times of scarcity, ibid.; the rumex acetosa, or common sorrel, an article of food in Lapland, 72; corn spurry used in Finland for bread, xv. 267; case of voluntary abstinence on a religious account, 510; the nuts of the beech used as bread, xvi. 74; root of the arum maculatum a wholesome nutriment, xvii. 73; the arrowhead or Sagittaria, an article of food among the Chinese, 466; animal food salutary in the cure of pulmonary consumption, xix. 47; the seeds of the sea-pea saved multitudes from famine, 76; the roots of the heath-peaseling appease hunger, 77; case of diabetes cured by animal diet and cinchona, 181; luxury of the ancient Romans in regard to their diet, xx 345; the fucus used as such in a dried state, 350; the agaricus a luxuriant article among the ancients, 352; on the extent to which abstinence from animal food may be carried in diabetes, xxii. 110; advice in regard to the patient's diet in a case of schirrhous contracted rectum, 288; observations on the digestive quality of farinaceous seeds, 403; process of making bread, 405; on the original country of wheat, 407; unwholesomeness of bread made of diseased wheat, xxiv. 37; case of flatulency, with regurgitation of, 218; diet necessary to strengthen the stomach, 250; horse-flesh used in Norway, xxv. 283; vegetable diet tends to produce corpulence, 451; obesity reduced by abstinence from, 452; on the process of digestion in the human stomach, and the sensations of hunger and thirst, 471; the effects of forced abstinence, 472; reasons for not eating animal food, xxvi. 310; other reasons for eating it, xxvii. 47; necessity of abstemious diet in the cure of cancer, 69; account of that of the, 124; on the quantity supplied in London, 169; account of a work entitled Return to Nature; or a Defence of the Vegetable Regimen, 312; no particular regimen necessary in calculous complaints, xxviii 38; rules for diet by Suvorof, xxix. 49, on the digestive powers of various animals, xxx. 108; on the process of digestion in the stomach, 433; experiments on different kinds, 517; strong animal food recommended in consumption, xxxii. 227; observations on digestion, 257; the Chinese unacquainted with the use of bread, xxxiii. 248; experimental observations on digestion in man, 420.

FOOKS, Ann, a young woman who vomited urine, account of her case, xxx 44; extraordinary call upon Dr. Yeats on that subject, 176; his reply in explanation, 197; particulars of her case, attempting to prove her an imposture, 355; the case confirmed by facts, 465; attack upon the relation of her case, 481; Dr Yeats in reply and explanation, 488; her character vindicated, xxxi. 19; answer to Dr. Yeats on his relation of the case, 21; farther account of the case by Dr. Yeats, 25, 106; that account confirmed by other instances, 111, 114; observations on the case, 124, 196; review of the case, with arguments to prove it a deception, 205, 283; cases in support of her veracity, 359.

FOOT, case of chronic intermittent suspended by a scald of the, xix 53; experimental observations on the structure and uses of that of the horse, xxiii. 175; series of original experiments on the same, 338; utility of the foot-bath or pediluvium, xxvi. 400; tetanus produced by a prick of the toe, 403; farther account of experiments on that of the living horse, xxvii. 425; description of a new foot-stool, with an engraving, xxx. 174; anatomical observations, with an engraved representation of the integuments of the human, xxxi. 461; on the use of opium in dry gangrene, when it attacks the toes, xxxii. 430.

———, Jesse, his cases of the successful use of vesicæ loturæ for the cure of the diseased bladder, xi. 82; his claim to the discovery of a membranous fence in the urethra, xxv. 41; case of too small perforation of the glans penis, 42; explanation of Mr. Ramsden in answer to him, 118; announcement of his answer to Mr. Home on the diseases of the prostate gland, xxvii. 257; his communication relative to the success of the cow-pox in the island of Jamaica, xxviii. 78; his review of Mr. Everard Home's observations on the diseases of the prostate gland, xxix. 226; on the discovery of the middle lobe of the prostate gland, 238; his case of diseased prostate bladder and rectum cured by injections of warm water and doses of uva ursi, xxxi. 225.

FOOT, Dr. Malachi, his relation of facts on the noxiousness of rain-water and dew in America, xix. 494; his observations on the functions of the liver, and on the importance of that organ as a seat of morbid affections, xx. 339.

FORAMEN, case of fracture of the occipital bone extending to the great, xxviii. 63.

FORAMEN OVALE, account of the discovery of the valve in it, ii. 372; erroneous opinions on the same, ibid.; case of the non-closure of it in an infant, xxii. 78; observations on the dilated, xxxii. 516; xxxiii. 49.

FORAMINA, those of the first vertebra of the neck rejected by ingressions, ii. 162.

FORBES, Charles, on the anti-venereal power of acids, illustrated by cases, v. 73; his observations on an epidemic ophthalmia, xviii. 578.

———, Dr. Duncan, his system of medical instruction, viii. 576; on the origin and progress of empiricism, xiv. 437; continuation of the same, xv. 362; anecdotes of quackery, 365, 366, 370.

———, Rev. Patrick, his observations on the cause of death from what is called the wind of a ball, xxix. 63.

———, John, observations by him on tropical nyctalopia, or night-blindness, xxvii. 165

———, William, his observations on the distemper in dogs, vi. 314; farther observations on the same, 466; his cases of vaccine and variolous inoculation, xiii. 517; on the use of sulphurated hydrogen in stomachic complaints, xv. 274; cases of small-pox in the fœtus, xviii. 177; description of a steam-bath, with its effects in gastritis, xxiv. 162.

———, Mr extracts from his Oriental Memoirs on the Natural History of India, xxxiii. 338.

FORCEPS, description of new invented, for extracting teeth, i. 15; description of a new one for extracting balls, vii. 454; on that commonly used for gun-shot wounds, with account of improvements, ix. 2; account of the ball forceps, with plates, 3; on the utility of the forceps in hastening delivery, xi. 330; polypus removed from the nose by an improved one, xiv. 179; proper method of using it in midwifery practice, xxi. 384; suggestions for the improvement of that used in lithotomy, xxv. 148; on the superiority of the forceps in difficult labour over the vectis, xxvi. 111; on the manner of applying it, 112; on the adjustment of the instrument, 114; how liable to failure in lithotomy, 287; improvement in the midwifery, 410; elementary instruction in the use of it, xxvii. 346; description of new ones for lithotomy, xxxiii. 3

FORD, Edward, on the causes of hydrocephalus internus, ii. 261; his case of diseased bladder, iv. 390; explanation of the plate accompanying the same, 393.

———, Dr. John, review of his letters on medical subjects, xi. 561.

FORDHAM, E. P. his case of polypus vaginæ, with an engraved representation, xxvi. 38.

FORDYCE, Dr George, his practice of physic translated into German, i. 88; also his treatise on fever, 200; review of his third dissertation on fever, ii. 393; on inflammation of the brain, vi. 507; on the causes of apoplexy, 559, *note*; review of his fourth dissertation on fever, viii. 275; his observations on intermittents, 417; his trial of the efficacy of bark in gout, xii. 157; his method of treating scarlatina anginosa, xv 493; his experiments on the chyle, xvi. 200; on the renovation of impure air by fumigation, 449; confirmation of his opinion on the action of aqueous vapours, xxiii 310; one of the founders of the Lyceum Medicum Londinensi, xxvi. 384; maintained fever to be a disease of the whole system, xxxii. 183.

———, Sir William, recommended muriatic acid in ulcerated sore-throat and putrid fever, xi 291.

FORESTIER, M. account of his Dissertation de Morbis aut noxis puerorum in vitiatis, depravatisve parentibus, xxi. 331.

FORGES, remonstrance against them in the reign of Queen Elizabeth, xi. 445.

FORMAN, Mr. his contrivance for taking and preserving vaccine matter, xxxi. 432.

FORMATIONS, account of analyzing primitive ones, as observed in the Highlands of Scotland, xxxi. 76.

FORSKEL, M. his description of the balm of Gilead, i. 37.

FORSTEMANN, Dr. his cases of nervous affections of the face, xxxi. 184.

FORSTER, Thomas, on a hernial sac within the tunica viginalis, xi. 377; his meteorological diary at Clapton, xxiii. 437; xxiv. 176; observations on the emigration of swallows, xxv. 86; his attention to English botany, xxv. 371; his observations on an occasional increase and decrease of the hair, xxvi.

308; his case of tumours in the thorax of a fowl, xxviii. 381; his case of bubonocele requiring a second operation, xxxiii. 411.

FORSTER, Reinhold, his translation of Scheele's Treatise on Air and Fire, iii. 336.

FORSYTH, Mr. his discovery of a remedy to save trees from insects, i. 198; his method of preserving fruit-trees from insects, 304.

FOSBROOKE, Rev. Thomas, on the eruptions succeeding vaccine inoculation, iii. 247.

————, Mr. his report of cases of synocha in the Durham Hospital, xxiii. 159; on the efficacy of cold affusion in fever, ibid.

FOSSI NARICULARIS, account of extraordinary large calculi extracted from the, ix. 260.

FOSSILS, society established in America for the investigation of, i. 86; potass found in the decomposition of the lencit, 300; the mineral or fossil alkali formed in the salsula soda, 301; description of the melilithus, iii. 584; new earth found in the Saxon beryl, 587; analysis of the same, iv. 82; analysis of a fossil stone found in Sweden, 185; immense fossil bones found in America, 186; account of a volcanic fossil in Iceland, 377; new earth found in a Swedish, vi. 188; on new analysis of the melilithus, 189; water contained in fossils, 375; properties of the agust earth in the Saxon beryl, 473; analysis of one containing arsenic acid, 570; fossil palms found in the mines near Cologne, vii. 373; a fossil fish found in a quarry near Paris, x. 480; analysis of the agusbit, or Saxon beryl, xi. 285; announcement of a treatise on, 384; chemical analysis of a Swedish fossil, in which a new earth was discovered, xii. 69; discovery of the bones of one resembling a crocodile in Gloucestershire, xv. 391; discovery of a new one in Devonshire, with its analysis, xvi. 191; the leaves of the osmunda regalis frequently found in the nodules of iron stone at Coalbrook Dale, xix. 542; account of an Elementary Introduction to the knowledge of extraneous fossils, xxi. 351; on some peculiar ones in several species of marbles, 439; discovery of the cranium of an unknown and horrid animal, xxiv. 87; description of the remains of a fossilized crocodile, with an engraving, xxv. 97; on the fossil relics of animals in Britain, ibid.; account of a fossilized sloth in Spain, 99; of the claw of a foot of a monstrous animal in Bullock's Museum, ibid.; description of remarkable fossil bones found in America, xxvi. 17; inquiries into the organic remains of the ancient world, xxvii. 23; on those of unknown animals, 24; on those in the caverns of Germany, 25; on those of France, 27; account of those found in London, ibid.; those of the isle of Sheppy, 28; the Irish fossil elk, ibid.; new varieties of Alcyonia found in the Isle of Wight, xxviii. 237; on specimens of hippurites from Sicily, 238; organic remains in the island of Barbadoes, 239; remarkable tribes found in Lancashire, ibid.; deductions from facts observed of the organic remains of the former world, 304; observations on the process of petrifaction, 305; dimensions of a fossil crocodile found in Dorsetshire, 516; account of vegetable bodies in the dendritic variety of chalcedony, xxix. 430; enumeration of fossilized remains found at Brentford, 507; account of a fossil chama ear, 511; of a fossil turtle found in Dorsetshire, ibid.; on the blue colour in fossil teeth, 519; the petrified head of a cetaceous animal found at Antwerp, ibid.; the jaw-bone of a rhinoceros found near Abbeville, 520; discovery of fresh-water shells near Paris, ibid.; similar beds found in Spain and Germany, ibid.; account of Cuvier's large work on the fossil bones of quadrupeds, ibid.; remarkable ones found at Highgate, xxx. 343; description of a fossilized human skeleton brought from Guadaloupe, xxxi. 338, 432; account of two Alcyonia found at Lime-Regis, 340.

FOSTER, Thomas, his case of an extraordinary monstrosity, ii. 226.

————, Thompson, his case of lithotomy, with cautions on selecting the proper state of a patient, xxii. 155; his testimonial in behalf of vaccination, xxx. 86.

FOTHERGILL, Dr. Anthony, account of his Essay on the Preservation of Shipwrecked Seamen, iii. 285; his case of extra-uterine fœtus, xv. 385; his case of tic douloureux, xxv. 167; his bequest to the Medical Society of London, xxx. 522.

————, Charles, account of his Researches on the Zoology of Great Britain, xvii. 392; his Essay on the Philosophy and Study of Natural History announced, xxix. 85; account of that work, 501.

————, Mr. (of Selby, Yorkshire,) on the influenza in that neighbourhood, x. 393.

————, Dr. John, his observations on the sick head-ach, i. 286; on the dolor facei or tic douloureux, ii. 465; compared the animal body to a clock, iii. 47; considered the apoplexia hydrocephalica a hopeless disease, v. 125; his account of the dolor facei not original, vi. 158; his observations on the same, 159, 160; on the causes of apoplexy, 552; his method of treatment in that disorder defended, vii. 139; his theory and peculiar treatment of apoplexy controverted, 76; prize medals in honour of, 400; on scarification in hydropic diseases, 406; his case of apoplexy, with remarks, 442; his observations on apoplexy censured, 444; account of the ulcerated sore-throat observed and described by him, xiv. 369; causes of his popularity, 370; treated the scarlatina anginosa with emetics and bark, xv. 494; against emetics in some cases of bilious vomiting, xvi. 381; questions for the Fothergillian medal, xvii. 198; on the time when the hydrophobic symptoms appear, xix. 244; communication to him on the external use of cold water in fever, 272: account of the Fothergillia Alnifolia, an American plant, called after him, xxv. 94; cured fæmia with oil of turpentine, 168; anecdote of him, xxvi. 456; his analysis of Cheltenham waters, xxx. 300; his communication on the tic douloureux to the London Medical Society, xxxi. 180; his character, 383; observations on his use of hemlock in nervous affections of the face, ibid.

FOTHERGILL, Dr. Samuel, announcement of his treatise on the Tic Douloureux, xi. 192; review of his Concise Account of a painful Affection of the Nerves of the Face, xiii 367; account of his practice in the Westminster General Dispensary and the Western Dispensary, xv. 386, 483, 578; xvi. 90, 185, 280, 380, 467, 571; xvii. 74, 183, 289, 387, 482, 579; observations on his treatment of rabies canina, 328; his medical reports for 1807, xviii. 91, 182, 285, 371, 482, 569; xix. 90, 186, 283, 368, 475; his use of aqua ammoniæ in tic douloureux, xxxi. 386; on the treatment of scarlatina anginosa in the Asylum for Female Orphans, xxxii. 481.

FOUNDLING HOSPITALS, observations on the inexpediency of erecting, xiv. 271; account of vaccine and variolous inoculation at that in London, 506; farther account of vaccine inoculation there, xvii. 488; report on vaccination at that of Dublin, xxiv. 522; cessation of the small-pox in the, xxvi. 35; account of the practice of vaccination in that institution, 392.

FOURCROY, M. on the properties of nitrous acid, i. 31; account of his chemical opinions, 82; on the combustion of phosphorus, 85; on the application of pneumatic chemistry to the cure of diseases, 143; his experiments on the constituent parts of urine, 193; account of his observations on the discoveries of Mayow, 194; analysis of his observations on Mayow's experiments, 249; his process for artificial freezing, 196; on the composition of unguentum hydrargyri, 357; discovers the existence of gluten, albumen, and jelly, in vegetable bodies, ii. 76; on the utility of oxygenated pomatum in leprosy, iii. 167; his analysis of a gouty concretion formed in the fingers of a man, 174; his account of a new method of combining oxygen with suet, to make oxygenated pomatum, 580; his discovery of sulphurated hydrogen in the kermes mineral and sulphur of antimony, ibid.; on the depletion of the lymphatics, vi. 139; on the decomposition of sulphats, 242; his examination of the colouring part of the blood, 469; his analysis of the ear-wax, 475; on the effect of oxalic acid on the bones, 476; on the urinary stones and gravel in men, 527; vii. 60; his opinion on corrosive sublimate refuted, vii. 518; on the acid in the urine of horses, viii. 476; his experiments on mercurial salts, ix. 9; on urinary stones and gravel, 242; on substances found in urine of healthy persons, 350; on muriat of potash in urine, 312; on the nature of urinary calculi, with plates, 489; his opinion on the combination of carbon and phosphorus controverted, xi. 179; on cork as a constituent of vegetables, 288; his discovery of a new metallic substance, 479; on the formation of carbonate of lead, 571; his observations on urinary calculi, xvi. 159; on the action of the alkalies upon uric acid, 310; translation of his Chemical Philosophy into English, by W. Desmond, xviii. 573; his errors noticed, 575; his analysis of calculi extracted from the bladder of a dog, xxi. 369; on the reciprocal action of sulphur and charcoal, 527; on obtaining a concrete sugar or manna from the rhododendron ponticum, or dwarf roseberry, xxii. 261; his analysis of the smut of wheat, xxiv 37; on the power of the concentrated nitric acid in precipitating urea from its solution in water, 327; an error of his in regard to

Mr. Wedgewood's thermometer, xxvii. 382; on the urine of the ostrich, xxviii. 470; biographical account of him, xxx. 499; his observation on the presence of magnesia in human bones, xxxiii. 159.

FOURNIER, M. on the medicinal properties of oxygen, ii. 38; description of his apparatus for determining the quantity of spirit contained in any liquid, xxii. 351.

FOWLER, Dr. the first who used arsenic in the cure of intermittents, i. 187; on the efficacy of digitalis in consumption and dropsy, 291; farther observations on the same, ii. 70; his caution in reporting cases, 114; on the vague accounts of the virtue of digitalis, iii. 133; his account of the influenza as observed at Salisbury, x. 386; effects of his mineral solution in lepra and other diseases, xv. 297; on the different effects of galvanism and electricity on animal substances, xvii. 190; facts proving the efficacy of his mineral solution in chronic rheumatism, xviii. 250; and in tic douloureux, 252; on the alleged failures of vaccination at Ringwood, xix. 329; his solution beneficial in chronic rheumatism, 369; benefit of his solution in cutaneous complaints, xxx. 296, 422.

———, Thomas, two cases related by him of the poisonous effects of the thorn-apple, xxv. 500.

———, William, on the fatal effects of decayed teeth, iii. 55; his observations on the perpendicular extraction of teeth, viii. 119; on the situation of the molaces, 120.

FOWLE, Dr. William, on the fevers of the West Indies, iv. 354.

FOWLS, account of some poisoned by phosphorus, i. 85; analysis of their excrement, 505; remarkable worms observed in, ii. 204; pustulous eruptions peculiar to them in the East Indies, xi. 431; the leaves of the nettle good for, xii 37; on the practice of the Romans in enlarging the livers of, xx. 345; tumours observed in the thorax of, xxviii. 381.

FOX, observations on the odour emitted by the, vi. 439.

———, Joseph, review of his natural history of the human teeth, x. 77; animadversions on his opinion respecting the ocularity of the teeth, 288, 360; new volume on the diseases of the teeth announced, xv. 96; analysis of that work, 371; remarks on the same, xviii. 210; on stopping decayed teeth, 553.

———, Mr. of Canada, his zeal for the introduction of vaccination in that province, xv. 311.

FOX, Dr. Joseph, account of his Medical Dictionary, ix. 285.

FOXGLOVE, see *Digitalis.*

FOXYGEN, a term improperly given to azote by the French chemists, ii. 185.

FRACASTORIUS, epidemics ascribed by him to a pestilential constitution of the atmosphere, xiii. 209; his observations on the English sweating-sickness, ibid.

FRACTURE, on the use of the trephine in a fracture of the cranium, illustrated by a case, i. 328; formula of an excellent plaster for fractures, ii. 91; utility of flannel rollers in, ibid.; the darnel externally applied by Celsus in fractures of the ribs, 106; observations of Jacobus Berengar de Carpi on fractures of the cranium, 279; on that of the collum osis femoris, 365; account of early improvements in the trepan, 366; three cases of fractured skull, iii. 23; cases of injuries to the head, with observations on the use of the trephine, 30; case of recovery from fracture of the cranium, 444; case of compound fracture of the leg, with a considerable protrusion of the tibia, illustrated by an engraved representation, 549; successfully treated with adhesive plasters, 550; description of a bandage for fractures of the patella, iv. 286; case of that of the cranium, 346; successful treatment of fracture of the skull in a sailor who fell from the mast-head, 513; case of fracture of the cranium attended with unusual circumstances, v. 255; observations on simple fractures where the union fails, vi. 201; general remarks on fractures and their treatment, illustrating cases of failure of union, 397; reply to that paper, 477; case of violent fracture of the cranium, 505; case of fracture of the occiput by a fall, vii. 53; new method of healing effusions under the skull after fractures of head, 216; account of the method of the treating compound fractures, by Messrs. Crowthers, in Yorkshire, 307; on the formation of new bone after compound, 418; on the treatment of that of the maxillary bone, ix. 169; how the brain is injured by fracture of the bone, 272; case of fractured cranium, 273; use of the trephine, ibid.; on the fracture of the thigh and the treatment, 278; description of an improved trephine, with a plate, x. 239; case of that of the cranium, 244; case of compound fracture of the leg, 316; benefit of musk and ammonia in it, 318; abscess of the chest occasioned by fracture of the ribs, 414; case of fractured skull, xi. 36; case of compound fracture of the thigh, 37; case of fracture of the occipital bone, with loss of part of the cerebellum, xii. 15; description of an improved trephine, 201; case of fractured skull successfully treated, xiii. 295; case of fractured cranium at sea, 445; observations on fractures of the cranium, with cases, 526; case of fractured thigh cured by nitric acid, xiv. 29; remarks on the treatment of fractured skull, 161; case of compound fracture of the humerus from a gun-shot wound, 267; on the cure of unnatural articulations in fractures of the extremities, 476; on the treatment of fractured thigh, xv. 8; description of a machine for fractured thighs, 339; case of fungous excrescence after fracture of the scalp, xvi. 149; extraordinary case of fracture of the cranium successfully treated, xviii. 129; case of compound fracture of the ankle joint, 162; cases of fractured sternum, with observations, 200; case of fractured scapula, 309; another case of fracture of the sternum, 415; observations on the French and Italian treatment of fracture, xix. 80; on demiflexion in the reduction of fractures, ibid.; repose necessary in fracture, 81; on the proper application of bandages in fracture, ibid.; prize question on fractures of the bones of the trunk, xx. 286; case of fractured cranium, with a tumour succeeding it, xxv. 165; case of fractured vertebræ, with a new mode of treatment, 201; new method of securing the fractured os femoris, ibid.; observations on fractures of the thigh, and suggestions for an improved treatment of such accidents, xxvi. 120; description of a bandage for that purpose, with an engraved representation, 122; case of fractured spine, with a plate, 371; case of fractured os pulvis, 375; case of extensive fracture of the skull, with wound of the meningeæ artery, xxvii. 435; case of ununited fracture of the thigh cured by sawing off the ends of the bone, xxviii. 59; case of fracture of the occipital bone extending to the great foramen, 63; observations on those of the surface of the earth, 75; case of caries in the second cervical vertebræ, and consequent fracture of its dentiform process, 131; case of fractured cranium, where the dura and pia mater were lacerated, and part of the cerebrum protruded, 154; case of trismus occasioned by a compound fracture of the leg, 155; local bleeding and purgatives necessary in compound fractures, xxx. 464; mode of preventing the shortening of the limb in broken femur, 516; case of fracture, attended with unusual symptoms, relieved by a longitudinal incision through the fascia of the limb, xxxi. 214; case of compound fracture of the os humeri, 456; cured in Barbary by ostrich grease, 478; extensive fracture of the cranium, with laceration of the dura mater and cerebrum, 485; case of compression of the brain from two fractures, xxxii. 8; new mode of treating fractures of the extremities, with a plate, 177; singular case of recovery after the loss of a portion of the brain, 184; observations on the good effects of vesicatories in promoting union in cases of fracture, which may not have united naturally, xxxii. 470; directions for the right position of limbs in fractures, 471; on fractures of the thigh, 472; on fracture of the fore-arm, 473; on bad cases of compound fractures, 474; on compound fracture of the joint, ibid.; case of fractured cranium, with loss of some of the brain, 525; on fractures of the extremities, with the description of an elastic iron cradle for the leg, xxxiii. 164; explanation of the plates illustrative of the same, 171; description of an annular saw for fractured bones, 189; case of compound fracture of both legs successfully treated, 332; case of fracture of the neck of the thigh-bone, 469; improvement in the treatment of fractured bones, 508.

FRADIN, M. his apparatus for preventing the noxious effects of breathing an impure atmosphere, xxxi. 80.

FRACHM, M. a Danish surgeon, his method of treating inveterate ulcers, i. 183.

FRÆNUM, observations on cutting that of the tongue, xiv. 97; farther remarks on the same, 256; calculi found on the, xxxii. 169.

FRAMBÆSIA, or the Yaws, how mistaken for the venereal disease on the discovery of America, xiii. 90; description of the disease, xv. 382; historical observations on the same, 383; whether the same with the leprosy, 385; minutes of a case cured by a peculiar treatment, xix. 122; particulars of the remedy employed, xx. 401.

FRAME, construction of an elastic one for the conveyance of sick wounded persons, xx. 142.

FRANCE, account of the hospitals at Paris, i. 80, 81; state of surgery in that country, 81; account of the proceedings for regulating a standard for weights and measures, 195; intense cold there, 196; number of mineral springs in France, 305; new medical publications in, 416; state of medical and surgical science in, ii. 98; disputes there in the 16th century on the rights of surgeons, 157; new medical publications in, 200, 400; iii. 287, 395; iv. 475; vaccine inoculation introduced there, iv. 88; publication of the memoir of the Natural History Society of Paris, 272; plan of an hospital for hydrophobia in, 489; encouragement of vaccination in, 470; on the introduction of vaccine inoculation at Paris, 251, 470; reports on vaccination there, v. 99, 356, 554; natural history of salamanders in, 177; establishment at Paris of artificial mineral waters, vi. 185; address of the Board of Health there on the benefit of vaccination, vii. 201; on the turbidity of the waters in, 477; report to the National Institute on artificial mineral waters, viii. 86; prize question of the Medical Society at Bourdeaux, 280; account of disorders in the French army when employed in Egypt, 452; prize question of the Institute of Health at Nismes, 478; program of prizes proposed by the Society of Montpellier, ix. 91; prize questions of the Society at Paris for promoting natural history, 95; remarkable cold at Paris in 1803, 287; report of the Academy of Sciences at Dijon, 371; account of a catarrhal fever prevalent at Paris, 419; report on the means of preventing contagion in, xi. 97; report on electricity and galvanism in, 523; French report on alleged failures in vaccination, xii. 348; great encouragement of vaccination in that kingdom, xiii. 419; plan of a society for exterminating the small-pox, 423; extraordinary disease among miners in, xv. 93; report of the Central Committee of Paris on vaccination, 314; report of the National Institute on manufactures prejudicial to health, xvi. 100; number of persons vaccinated there in 1806, xvii. 63; singular disease in, 472; state of medical science in, xviii. 34; method of promoting knowledge by prize questions in that country, 35; list of cities there which contain a population of thirty thousand and upwards, 96; excessive heat at Paris in 1807, 288; prize question of the National Institute on the subject of phosphorescence, 289; proposal of the faculty there to remunerate Dr. Jenner, 341; number of lazar-houses in that country formerly, xix. 378; improvements of anatomical knowledge in, xx. 3; botanical investigations of the National Institute, 16; report on a contagious disease in some parts of, xxiii. 33; proceedings of the Medical Society of Paris, 259; organic diseases of the heart prevalent there, 277, *note*; regulations for pressing conscripts for the French army, 429; annual report on the state of medicine and surgery in, 451; account of an epidemic disease there, 455; prevalence of hydrophobia in, 525; report on the doctrines of Gall and Spurzheim, xxiv. 4; comparison between France and England in regard to surgery, 17; report of the Society of Pharmacy at Paris, 224; quackery in, 430; important report on the success of vaccination in, xxv. 181; report of the National Institute, 362; improvement of medical knowledge in, 454; prize offered there for a machine to spin flax, 545; enumeration of other prize questions on the part of the government, ibid.; enumeration of new French publications in 1811, xxvi. 347; the population of that country improved by vaccination, 393; proceedings of the Institute of, 420; visionary ideas of the French naturalists, xxvii. 22; transactions of the physical department of the same, 374; account of the principal medical establishments at and near Paris, xxviii. 461; character of the French nation, 476; proceedings of the Imperial Institute in 1812, xxix. 511; on the encouragement afforded the government there to vaccine inoculation, xxx. 117; establishment of warm-baths and douches at Paris, 223; transactions of the Imperial Institute continued, 251, 429, 517; proportionate number of failures in vaccination in, xxxi. 142; prize questions of the Pharmaceutical Society of Paris, 517; prize papers on the nature and treatment of croup, 518; report of the state of science there in 1813, xxxii. 222; report of diseases in Paris, 255; new specifics announced for gout in, 523; unabated state of civilization in that country, xxxiii. 71; severe disease among horned cattle there, 161; exposé of the state of medicine in 1814, 298; account of the proceedings of the Institute in the departments of physiology, anatomy, and surgery, 396; inquiry into the disease among horned cattle in, 401; on the cruelty of the French physiologists, ibid.; report of the Institute on the manuscript of a work on poisons, 471; particulars of a scientific tour in, xxxiv. 219; on the frequency of gangrene in the French hospitals, 405; sketches of the Medical Schools at Paris, 478; proceedings of the Royal Institute of France, 524.

FRANCE, (Isle of,) accounc of a plant of the orchis family brought from thence, i. 197 ; introduction of vaccination there, xv. 523; account of the population of, xxx. 506; first appearance of the small-pox there in 1756, 507 ; its destructive ravages in 1772, ibid.; extermination of that disease by the precaution óf the inhabitants, ibid.; farther account of vaccination at that place, 508.

FRANCILLON, M. his discovery of a new metal, viii. 573.

FRANCIS, Dr. J. W. his observations on the effects of mercury in its native state, and its abuse in certain diseases, xxix. 207.

FRANCO, Pierre, a French surgeon in the sixteenth century, the inventor of the high operations for the stone, ii. 155; his method of applying the actual cautery in vericocele, 367; particul.rs of his success in lithotomy, x. 5 ; the bladder not distended by him, 6, *note.*

FRANCONI, M. his success in taming and training a stag, xxiii. 85.

FRANK, Dr. D. on the medical virtues of the citrat of iron, i. 401.

———, Dr. G. R. his comparative view of the principal theories of chemistry, i. 63, 167, 170, 269, 368.

———, Dr. J. P. on the best method of rearing children, i. 107.

———, Dr. Joseph, on two different kinds of diabetes, i. 411; review of his instructions for the knowledge of a physician, v. 475; his cases of diabetes aquosus cured by mercurial friction, vii. 67; account of his visit to Dr. Beddoes, xxv. 345.

———, Dr. L. on the bracing virtues of the warm-bath, ii. 466.

FRANKINCENSE, used by Hippocrates for ulcerations, asthmas, and other complaints, xii. 471.

FRANKLIN, Dr. Benjamin, his character of the University of Edinburgh, vii. 43; on the hydrogen gas evolved in marshy situations, 120; on his hypothesis of a single electric fluid, viii. 477; his resumption of animal food, xxvi. 310.

FRANKLYN, F. E. on the internal use of nitras argenti combined with the shower-bath in chorea sancti viti, xxxiii. 273.

FRANKS, John, his explanation of the Brunonian doctrine, i. 130; announcement of his treatise on the contagion of typhus, 201; review of that work, ii. 195; his defence of the Brunonian system, 449; iii. 49; on the advantages of variolous inoculation, iv. 518; reply to him on that subject, v. 31; farther observations by him on the superiority of small-pox inoculation, 81, 146.

FRASER, Dr. Henry, answer to him on the subject of secondary small-pox, xv. 169, 171; his case of diabetes mellitus, 276; account of his observations on vaccine inoculation, 288; account of his essay on Epilepsy, and the use of the viscus quercinus in the cure of that disease, xviii. 17.

FRAXIMUS, or common ash-tree, botanical description of the, xi. 444; its uses, 445.

FRAXINELLA, on the sensible perspiration of that plant in summer evenings, xxv. 520.

FRAZER, Dr. his account of the influenza at Wisbeach, x. 204, 206.

———, Mr. his queries on the epidemic at Gibraltar, xxxiv. 301.

FREAKE, A. his account of the efficacy of medicines prepared from the humulus lupulus, xiii. 432; case of scorbutic humour relieved by the same, 433; benefit of the same in gout, 434; composition of the tincture, 435; on the virtues of the same in gout, xvii. 386; addenda to his paper on the humulus lupulus, xviii. 403; additional cases in the same, xxv. 169; directions for the use of that remedy in gouty cases, 171; his trial of the virtue of humulus or hop in gout, xxix. 294.

FRECKLES, removed from the skin by the leaves of the drosera rotundifolia, xiv. 64; benefit of the convallaria polygonetum in cleansing, 67.

FREDERIC the Great, (of Prussia,) on his care of his health, ii. 494.

FREEMAN, R. his observations on an epidemic scarlet fever and sore-throat at Cheltenham, ix. 157.

———, Richard, his report of the Suffolk Benevolent Medical Society, xvi. 94.

FREER, Dr. Adam, his inaugural dissertation de Syphilitide Venerea, xxiv. 24; he maintains the animalcular origin of diseases, ibid.

———, Mr. his case of the suppression of urine, vi. 526; his successful cases of aneurism, xxvii. 30.

FREEZING, method of effecting it by artificial process, i. 196; the mercury in the thermometer sinks 30° below the freezing point by the application of muriate of lime and snow, ii. 77; case of the brain frozen, 146; experiments at Hudson's Bay on frozen quicksilver, xviii. 289; new method of effecting it artificially, xxiv. 397; that process described, xxvii. 430; quicksilver frozen by artificial process, 520; description of an instrument for freezing water, xxix. 173; new method of freezing alcohol, 431; on the gangrene produced by, xxxiii. 299; on the phenomena of, 300; on the slowness of the process, xxxiv 41; boiling water sooner freezes than any other, 42.

FREMY, M his method of preparing acetate of potash, so as to obtain it white and in a saturated state, xxv. 519.

FRETIS, Dr. Antonio de, his account of vaccination at Madeira, xxiii. 99; on the hooping-cough as it appeared in that island, 100; observations on his report, 179.

FRICKE, Dr. his method of applying electricity for the discovery of the tape-worm, i. 182, 277; description of his scintillometer, ibid.

FRICTION, benefit of medicated, in various obstinate diseases, i. 71; medicinal effects of opium externally applied by, 440; ii. 6; utility of antimonial tartar used externally, i. 71; benefit of opiate friction in typhus, 133; facts and observations on the external application of opium by friction, so as to be absorbed by the lymphatics, 440; additional facts and observations on the same, ii. 6; efficacy of corrosive sublimate applied by friction in venereal cases, 45; confirmation of the beneficial effects of tartarized antimony applied externally, 74; case of the good effects of opiate friction in typhus, 103; case of the same in gangrenous ulcer, 107; beneficial effects of that of opium proved in sphacelus, iii. 102; the leprosy cured by the external application of oxygenated pomatum, 167; the gastric juice applied by friction in several diseases, 168; opium and mercury externally used in venereal cases, ibid.; on the effects of acetic ether applied externally in rheumatic complaints, iv. 369; extraordinary powers ascribed to frictions in India, v. 465; use of oil frictions in arthritic complaints, 490; frictions of oil efficacious in dropsy and disorders of the genitals, vi. 72; effects of opium externally applied, 91; uses of the metallic brush, ibid.; acetic ether good to be applied outwardly in rheumatism, 273; phosphorus dissolved in oil beneficial in gout, 378; benefit of opiate friction in fever, 387; effects of the same in tetanic affections, 433; diabetes cured by mercurial friction, vii. 67; efficacy of opiate friction in different disorders, 124; efficacy of mercurial friction in inflammation of the maxillary sinus, ix. 425; frictions with oil recommended in yellow fever, xi. 480; friction with oil recommended to strengthen weak joints, xii. 470; case of constipation of the bowels cured by mercurial friction, xv. 325; efficacy of oily frictions in hot climates, xvi. 80; experiments proving that the friction of fluids does not excite sensible heat, xx. 97; benefit of mercurial friction in tetanus, xxii. 402; facts establishing the efficacy of opiate friction in spasmodic and febrile diseases, xxiii. 71; benefit of mercurial friction in tetanus, xxiv. 42; new methods of curing diseases by friction with various substances, xxvi. 315; experiments with camphorated friction and cantharides in urinary cases, 316; on the application of colocynthus in mania, 317; digitalis externally applied with effect in dropsy, 318; gold externally applied in venereal cases, 319; and in glandular swellings, 320; beneficial effect of friction in lameness after scarlatina, xxvii. 422; mercurial friction recommended in dysentery, xxviii. 35; cases of the efficacy of that mode of treatment, 67; extraordinary instance of its effects in palsy, 225; efficacy of mercurial friction in tetanus, xxix. 102; arsenic externally applied for the cure of herpetic affections, xxx. 26; case of anasarca in an old man cured by frictions of digitalis, xxxi. 432.

FRIEND'S RETREAT, account of a lunatic asylum near York, bearing that name, xxxii. 127.

FRIEND, Dr. extract from his History of Physic on the subject of bronchotomy, x. 6; his epistle to Dr. Mead on the confluent small-pox, xvi. 63.

FRIESE, Dr. on the application of mathematics to chemistry, ii. 298; his account of the successful practice of vaccination at Breslaw, ix. 128; on the internal use of mercurial ointment in venereal complaints, 487; on the use of digitalis in consumption, xi. 286; his account of the state of vaccination in Germany, with observations on the opposition to it, xiv. 233; appointed counsellor of the medical department of Silesia, 234; on the equine urus xvi. 245; on the impediments to the progress of vaccination, xvii 57; his plan for its encouragement, 58; his reflections on the opposers of the practice, 59

FRIGHT, hæmorrhage of the uterus occasioned by, ix. 388; disorder of the eyes caused by, xiv. 15; paralysis and derangement of intellect the effects of, 16.

FRIKE, Dr. on the use of lichen islandicus in phthisis, ix. 20.

FRITILLARIA REGIA, singular method of multiplying the growth of that plant, i. 297.

FROGLEY, R. A. his fatal case of pemphigus, xxxi. 265; observations on the same, 268.

FROGS, observations and experiments on the respiration of, ii. 79; on their organs of generation, 245; gelatine formed of their muscular fibres, 298; effects of nitre on the crural nerve of one, v. 587; account of the discovery of animal electricity in them, vii. 71; prepared ones used as electrometers, 160; effects of opium on, 348, 353; experiments on the hearts of, 352; viii. 344; live after being exposed to hydrogenous sulphurous gas, viii. 522; effects of alcohol injected into them, ix. 50; powerful effects produced in them by galvanism, 146, 487; on the electric susceptibility of the muscles of, x. 27; nettles poisonous to, xii. 32; experiments on them, xiii. 84; the frozen remains of them often found after a hard winter, xx. 272; frozen ones in Hudson's Bay recovered, xxvii. 486; experiments with the solution of opium on, xxxii. 276.

FROST, great one at Paris in 1798, i. 196; method of preserving apples from, 297; an intense one at Edinburgh in 1808, xix. 360; a gelatinous substance found after severe frosts, xx. 272; case of frost-bitten scrotum, xxii. 220; method of producing artificial ice, xxvii. 530; account of frogs frozen and recovering in Hudson's Bay, 486; on the artificial production of ice, 520; losses sustained by the French army in the Russian campaign of 1812, xxx. 20; benefit of melting frozen parts with snow and brandy, ibid.; curious description of the great one in 1813, xxxi. 268; comparison of it with former ones, 351; dreadful effects of it in Russia on the French army, xxxiii. 300.

FRUIT, how to preserve it from frost, i. 297; method of securing trees from insects, 304; eaten in hot weather the cause of diarrhœa, xii. 106; on the causes of the decay of apple-trees, xvii. 196; improvement in raising vines, ibid.; diarrhœa and cholera ascribed to the eating of fruits, xviii. 372; efficacious method of propagating fruit-trees of all kinds by cuttings without heat, xx. 569; Chinese method of raising fruit-trees, xxi. 86; remarkable distinction of winter fruits in fenny countries, 93; account of a new and expeditious method of budding in fruit-trees, xxv. 253; analogy between them and men, xxvi. 8; method of preserving it for several years, xxvii. 75; on the ascent of sap in, xxx. 251; on the organs of fructification, 253.

FRYDENBERG, Dr. his memoir on the utility of the seetang, an Iceland marine plant, xviii. 191.

FRYER, Mr. his case of extravasation of bile into the cavity of the abdomen, xxxii. 152.

FUCI, botanical description of its varieties, xx. 349; the food of various kinds of fish, ibid.; of the bladder fucus, ibid.; its antiscorbutic qualities, ibid.; how prepared, 350; beneficial in swellings, ibid.; account of the palmated fucus, ibid.; used as a febrifuge in the Isle of Skye, ibid.; eaten as a food, ibid.; description of the *fucus esculentus*, 350; recommended in the disorder called pica, 351; singularity of the characters of this family of plants, xxx. 255; the name of thelessio-phytes given to them in France, 256; experiments to ascertain the presence of iodine in different ones, xxxiii. 47.

FUEL, furze a substitute for coal in Cornwall, xix. 75; the fern used for heating ovens, 43.

FUGE, Samuel, on the efficacy of digitalis in consumption and dropsy, v. 209; on the benefit of vaccination, vii. 7.

FULLER, Francis, on the advantages of emetics in consumption, ii. 71; on the benefit of riding, 72.

———, Dr. Thomas, on the advantages of colt's-foot in scrofula, xix. 359, 360.

FULLWOOD'S RENTS, failures of vaccination in, xii. 384; report of the medical committee on the cases, 573; observations on the same, xiii. 53; misrepresentation of the cases, 170.

FULMAR, a sea-fowl in Iceland described, xxvii. 124.

FULMINATIONS, means of producing detonating ones by phosphorus combined with different bodies, i. 195; how oxygenated muriate of potash effects it by percussion, 196; explosion produced by nitric acid and silk, ii. 76; experiments with fulminating gold, 185; account of the accidental fulmination of sulphur and phosphorus, 186; preparation of the fulminating mercury, viii. 190; experimental observations on the decomposition of the fulminating powders of mercury and silver by charcoal, xxv. 457; that discovery applied to poison, 458.

FUMARIA FERMOSA, a plant brought from the north-west coast of America, described, xxiv. 530; description of the *fumaria nobiles*, xxv. 94.

FUMIGATION, beneficial effects of it in ships, i. 93; remarkable history of its supposed magical effects, iii. 246; inefficacious in destroying contagion, ibid.; farther observations on its inutility, 322; benefit of gaseous fumigation in pestilential fevers defended, 429; the nitrous fumigation successful in arresting gaol fever, 484; causes of its power, 485; effects of nitrous vapour on the atmosphere denied, 526; cessation of the practice in the fleet, v. 75; benefits of the nitrous fumigation in ulcers, at sea, 218; discovery of the anti-contagious power of nitric acid, vii. 424; antiquity of fumigations as a remedy against pestilential disease, viii. 186; historical account of the discovery of the efficacy of muriatic fumigation, 187; experiments on its benefit, 188; method of purifying the atmosphere by nitrous, 529; how to apply fumigations in sick rooms, 537; claim of Dr. James Johnstone to the discovery of the efficacy of mineral acid vapour in contagion, x. 78; on the use of aromatic ones against contagion, xi. 104; on the effects of nitrous vapours in preventing the spreading of infectious diseases, 136; success of experiments with, 137; constantly used with good effect in a voyage to Bengal, 510; injurious in the epidemical fever of Spain, xiii. 113; description of a permanent and portable apparatus for purifying the air, xiv. 190; correction of an error respecting the originality of nitrous fumigation in contagion, 573; the claim of Dr. James Johnstone to the discovery of the virtue of mineral acids ascertained, xv. 373; decree in Spain in favour of the anti-contagious fumigation, xvi. 190; impure air renovated by burning bodies, xvii. 449; used with success at Paris in the excessive heat of the atmosphere, xviii. 289; that of tobacco used among the Indians for pretended magical purposes, xxv. 125; on the use of the same in pestilence, 136; the fuming liquor of Boyle smokes in oxygen gas, but not in azotic or hydrogen, xxx. 256; benefit of the ammoniacal vapours in typhus, xxxi. 83.

FUMITORY, botanical description of the, xix. 71; efficacious in opening obstructions of the viscera, 72; classed by Cullen among the tonics, ibid.; beneficial in obstinate diseases of the skin, ibid.; useful in hypochondriacal and scorbutic cases, ibid.

FUNCHAL, in the island of Madeira, description of, v. 310; farther account of the bay of, ix. 311, 315; an extraordinary disease prevalent there, xxv. 428; fears of the inhabitants in regard to the infection of phthisis, 429.

FUNCTIONS, two classes of animal, vii. 469; those of the animal economy assist each other, viii. 13; account of organic ones in different animals, 367; on those of the placenta in pregnancy, ix. 259; on those of the lungs proving them to be fitted for the introduction of oxygen, xi. 46; analysis of those of the skin, xiv. 84; account of the new system of those of the brain indicated in the external form of the skull, 330; influence of the mind upon the animal, xvi. 92; inquiry into those of the spleen, liver, pancreas, and thyroid gland, 193; the opinions advanced by Dr. Rush in the preceding paper controverted, 289; on those of the liver, xvii. 508; those of the intellect best ascertained by studying physiology, xix. 386; that term substituted for the word organs, xx. 44; those of man the most interesting objects of science, 99; observations on healthy and diseased, 102; inquiry into those of the spleen, 211; on those of the liver, 339; on those of the stomach, xxii. 5; nature of the hepatic function, 421; importance attached to those of the uterus in regard to health, xxiii. 518; physiological speculation on those of the heart and arteries, xxiv. 9; on those of vegetables, xxx. 252; attempt to explain the organic actions in regard to the nervous functions, xxxiii. 23; farther observations on the same, 82; on the relation subsisting between the time of the day and various functions of the body, 125, 276, 351; analytical account of Gall and Spurzheim's system, 485; on the functions of the brain, xxxiv. 43.

FUNGI, observations on a poisonous species, with a case of its deleterious effects, iii. 41; case of poison from, xii. 385; description of the plant, 512; method of preventing their pernicious effects, xiv. 573; case of poison by, xv. 247; on the agaricus campestris, 248; botanical description of the several kinds of, xx. 352; some of them an article of luxury among the Romans, ibid.; used as an external styptic, 354; made into garments, ibid.; truffles a good esculent one, 355; narcotic quality of the lycopendon, ibid.; the mucor repels wet,

356; case of a family poisoned by, 457; description of the agaricus glutinosus, 460; another case of poison by the same, 566; benefit of vitriolated zinc and purgatives in such cases, ibid.; other instances of poison from them, 567; on the want of attention to the medicinal properties of, xxi. 13; fatal effects resulting from eating champignons in France, xxii. 503; account of a treatise on, xxiii. 68; observations on the deleterious, xxviii. 182; observations on various kinds of rotten-wood to illustrate fungi, xxix. 510; analysis of different ones, 521, 523; cases of persons poisoned by them in Scotland, xxxii. 364; description of the muscarius, 369; experiments with, 370.

FUNGIN, a name given to the fleshy part of mushrooms, xxix. 520; how obtained, ibid.; does not dissolve in alkaline solutions, 521; azotic gas disengaged in diluting it with nitric acid, ibid.; on its putrefaction, ibid.

FUNGUS, case of one in the eye, v. 241; on the cancerous fungus in the human breast, vii. 86; on the final cause of it in the human system, 88; its retrograde progress, 165; carcinomatous may exist without fungus, 167; effects of carbonate of lime on cancerous tumours, ix. 382; on cancerous, xii. 76; account of two cases of fungated sore, xiii. 366; case of fungous tumour in the abdomen of a woman, xiv. 79; case of fungous ulcer on the arm, followed by amputation, 514; pepper stonecrop good in destroying fungous flesh, xv. 266; case of it after fracture, xvi. 149; how used in cauterizing, xix. 358; on a peculiar affection of the testes, attended with the growth of fungus, xx. 171; fungous excrescences on the skin supposed to be hereditary, xxi. 330; on fungous tumours connected with the alimentary canal, xxvii. 507; on fungous excrescences of the bladder, xxxii. 156; on the treatment of the fungous cerebri, xxxiii. 403; only to be removed by operation, 405; method of performing it, ibid.; on the power of argentum nitratum in fungous sores, 465.

FUNGUS HÆMATODES, cases of it, x. 466; differences of opinion on the nature of it, xi. 210; various cases, with the mode of amputation, 211; on the hæmorrhage in the, 213; fatal when neglected, 215; explanatory observations on a case of fungus hæmatodes xiii. 7; differing from spongoid inflammation, 8; whether fed by the rupture of an artery, xiv. 516, *note*; two cases successfully treated by the external application of arsenic, xviii. 389; observations on the remedy, 390; analogy between that complaint and cancer, 534; may be cured without arsenic, 535; objections to the view taken of it by some practitioners as being too simple, xix. 215; cases in Dr. Monro similar to those of Mr. Hey, 216; farther observations on the same, 430; notice, with remarks on various works upon that subject recently published, xxiv. 11; description of its progressive appearances, 12; cases of hæmatocele bearing a new affinity to, 288, 293; originates in venous aneurism, ibid.; objections to the theory of Mr. Hey on, 302; case of successful operation on a boy, xxv. 303; history of a fatal case in a child, 448; on the motion of the blood in such cases, xxvi. 437; history of a remarkable case where the disease extended nearly over the whole frame, xxx. 234; case of that of the kidneys, xxxii. 19; appearances on dissection, 24.

FUNIS UMBILICALIS, case of its presentation in the evolution of the fœtus, iii. 5; on the rupture of the, 333; on the cause of the rupture, 464; cure of impervious, xiv. 483.

FURNITURE, directions for that of a sick room, x. 175.

FURZE, (*Ulex Europæus,*) botanical description of the, xix. 75; used in Cornwall for fuel, ibid.; profit arising from the cultivation of it in Ireland, ibid.

FUROR UTERINUS, observations on the causes of that disease, xxxi. 213.

FUSCHIUS, his observations on the virtue of digitalis purpurea, iii. 306.

FUTLYGHUR, in India, destruction produced by the fall of a ball of fire at, xxv. 457.

FYFE, Dr. on the variety of the quantity of carbonic-acid gas formed in the lungs, xxxiii. 247.

### G.

GADDIN, Mr. his discovery of a new earth in Sweden, iv. 185.

GADOBINIT, a new earth found in a Swedish fossil, analysed, vi. 188; farther analysis of it, ix. 474.

GAERTNER, Dr. his observations on the constituent parts of urine, and the luminous properties of touch-wood, ii. 298; farther experiments on the illumination of rotten wood, v. 583; account of his elegant work De Fructibus et Seminibus Plantarum, xxviii. 320; particulars of him, and the accuracy of his observations on the cotyledons of grasses, xxix. 510.

GAILLAUD, J. his method of preserving vaccine virus, x. 41.

GAIRDNER, Dr. on the efficacy of cold water in small-pox, xix. 273.

GALAXIA OVATA, a distinct species from the plant galaxia purpurea, xxix. 262.

GALBANUM, recommended by Hippocrates as an expectorant, xii. 471; the natural history of the, xxii. 33.

GALE, Rev. Mr. his successful cases of vaccine inoculation, v. 111.

GALEN, detection of errors in his works, ii. 61, 63; account of his zealous followers in the 16th century, 64, 65; made use of darnel in the cure of wounds, 94; maintained a fissure between the dental cells, 161; his error respecting the vertebræ of the neck, 162; his wrong notion of the muscles, 163; correction of his opinion on the intercostal muscles, 165; on the connexion between the breasts and uterus, 167; his opinion on the ventricles of the heart, 281; his innovations in medicine, 333; on the course of the blood in the fœtus, 371; defended against Vesalius, 460; ignorant of the axillary vein, 462; defended by Vesalius, ibid.; on a glandular substance in the stomach, iii. 73; his description of the cœcum, 75; his error on the pleura, 77; on the duct under the tongue, 78; on the origin of the nerves, 259; his salutary maxim, 329; denied the desiccation of the optic nerves, 356; defended against Vesalius, 460; the decline of his system, iv. 153; acquainted with the virtues of the remedy called Portland powder, in gout, v. 418; his observation on morbific enlargement of the tongue, vi. 524; on apoplexy, viii. 80; on delivering by the feet, 214; on dietetic remedies, 243; his high character, 280; his observation on leprosy, ix. 560; on tying the arteries in amputation, xiv. 41; on the flatulent effects of linseed, 63; his method of stanching the blood in amputation, 106; his observations on the empirics of his time, 440; on the affinity between cancer and elephantiasis, xv. 77; his description of phlegmon and elephantiasis, xx. 372; his distorted view of nature, xxi. 1; on dividing the spinal marrow, xxii. 163; his name applied to many receipts, 270; on the fall of his system, xxiv. 2; on antagonist muscles, xxv. 57; recommended bleeding and purgatives in gout, xxvi. 479; his system subverted, xxvii. 339; account of his work De Usa Partium, xxviii. 322; on pemphigus, xxxii. 281; age and character of, 300; his treatment of ulcers, 301; his use of the bandage, ibid.; on the treatment of aneurism, 302; his observations on peripneumony, 358; case of hydrophobia related by him, xxxiii. 320.

GALILEO, his researches led the way to the discoveries of Torncelli and Newton, vi. 410.

GALIUM VERRUCOSUM, or the Valantia Aperine of Linnæus, description of the, xxv. 92.

GALL, (the animal), observations on the alkaline basis of it, ii. 383; stones in the, viii. 243; its various uses, ibid.; that of oxen kills worms, xii. 470; observations on calculi in the gall-bladder, xvi. 3; specimens of biliary concretions found in it, 4; remarks on its uses, 201; answers the same purpose as the spleen to the blood, ibid.; on the bitterness of it, 202; the bile undergoes a change in it, 297; varieties observed in the calculi of the, xxix. 519; biliary concretions in the, xxxi. 158; extraordinary calculus in, xxxii. 169; case of extravasation of bile into the abdomen, from rupture of it, 152; that of a child contains bile, xxxiii. 154.

GALL BLADDER, case of rupture in the, xxv. 185.

GALL, Dr. announcement of his new work on the structure of the brain, iv. 88; account of his new doctrine of the brain, and the mental faculties, xiv. 327; farther details of his system, with an explanatory plate, xv. 201; his visit to the House of Correction and Hospital at Torgan, 203; description of the organs of the head, 213; particulars of his discovery, xvi. 178; declines in reputation, xvii. 295; divisions produced on the continent by his theory, xviii. 38; observations on his system, xx. 17; abstract of a French report on the same, xxi. 149; his opinions ascribed to wild enthusiasm, xxiv. 3; he and Spurzheim dissatisfied with the report of the French commissioners, 4; review of his anatomy and physiology of the nervous system in general, and of the brain in particular, xxxiii. 228; his early application to the study of the external form of the head, 229; particulars of his doctrine, 230; review of his Physiognomical System, as explained by Dr. Spurzheim, with an engraved representation, 485; on the manner in which the conclusions were drawn by him from his observations on the form of the cranium, 494.

his errors noticed, 495; his remarks on persons of a mechanical turn, 499; anecdotes of his practice, 501; his theory defended and explained, xxxiv. 309.

GALLIC ACID, experimental observations on it as an astringent principle, v. 362; method of preparing it, xi. 275; another process, xii. 382; quantity of it observed in tea, xxiv. 190.

GALLICIA, (in Spain,) method of curing hydrophobia in, iv. 87.

GALLINI, M. his compendium of physiology and pathology, ix. 437.

GALLITZIA, Prince, his experiments on the germination of seeds, iv. 84.

GALLOIS, M. le, on the seat of the movement of inspiration, xxx. 215; review of his work, entitled Experiences sur le Principe de la Vie, xxxi. 417; xxxii. 56; his death and character, xxxii. 80; his observations on the unequal capacities of the ventricles of the heart, 167; on the circulation of the blood in the fœtus, 168; his opinion on respiration confirmed by experiments, xxxiii. 156; biographical memoirs of him, 307.

GALLOWAY, in Scotland, on the middle granite district of, xxxii. 75; on the stratified rocks of, 76.

GALLS, their utility in dyeing, i. 166; experiments on the infusion of, v. 36; experiments to ascertain by them whether there is gelatine in cinchona, xi. 266; description of them, with their medicinal uses, xv. 69; good in hæmorrhoidal affections and intermittents, 70; benefit in dyeing, ibid.; on the extractive principles of, xxix. 480.

GALLUP, Dr. his successful case of hydrophobia, xxii. 54.

GALVANISM, on the practical utility of it, with a view to ascertain its effects on muscular and nervous irritability, i. 53; account of the experiments made by Humboldt, ibid.; a certain criterion for distinguishing death, 54; uncommon attention excited by the discovery, 55; importance of the discovery to medicine, 145; detail of Galvani's experiments as repeated in London, iv. 115; process of extricating the gases by it, 119, 126; experiments shewing the effects of nitre on the nervous fibre, v. 587; progress made in the improvement of it, vi. 209; experiments made at the Royal Institution, ibid.; description of the large apparatus there, 210; decomposition of water into oxygen and hydrogen effected by it, ibid.; decomposition of the metallic solutions, 213; its powerful influence, 214; experiments of Mr. Davy, at Bristol: works on the subject, 217; its effects on the functions of the nervous system, 416; applied to the blood, 513; historical account of that discovery, with notice of several publications on it, vii. 70, 159; its medicinal uses, with an engraving, 242; beneficial in asphyxia, 245; paralysis, ibid.; in nervous diseases, 246; weakness of sight, 247; in aphonia and hoarseness, 248; explanation of the apparatus, 249; in deafness, 257; experiments to confirm the phlogistic theory, 281; continuation of the history of the discovery, 539; its medical application, ibid.; general excitement produced by it, 530; its effects in genital irritation, 531; effects on the organ of sight, ibid.; on that of hearing, 571; proposed galvanometer, 533; cases of its efficacy in several diseases, with engraved representations, ibid.; description of the galvanometer, with a plate, 539; observations on its effects in the cure of certain diseases, with two plates, viii. 250, 315; in a case of aphonia, 251; on Volta's pile and its piles, 252; paralysis of the urinary bladder, 256; in white swelling, 257; in rheumatism and sciatica, ibid.; methods of applying it in diseases, 258; explanation of the plate, 259; in hoarseness, 286; on the action of the galvanic battery, 315; its quick penetration into the nervous system, 316; on the action of the simple galvanic chain, 317; on the diseases in which it may be best employed, 320; in paralysis of the extremities, 322; in amaurosis, 323; explanation of the second plate, 324; researches on the electric powers of the Voltaic pile, 478; experimental proofs of its power in the cure of deafness, 526; in tic douloureux, 527; in aphonia, 528; proposition for an extension of its uses, ibid.; applied successfully in a singular case of rheumatism, 552; observations and experiments on its medical virtues, ix. 131; method of applying it in diseases of the auditory organs, 133; method of applying it in paralysis of the sphincter vesicæ urinariæ, 135; in chronic hoarseness, ibid.; cases of paralysis of the extremities relieved by it, 136; cases of its efficacy in amaurosis, 138; cases of deafness cured by it, 142; experiments on galvanic contractions, 146; on galvanic effects in the muscular fibre, 147; on its effects when ligatures are made on different parts of the nerves, 148, 149; experiments on a malefactor, who was hanged in London, 195; announcement of the history of that case, 196; farther experiments on galvanic contractions, 235; on the conducting powers of charcoal and coke, 238; galvanic contractions excited under water, 239; influence of galvanism on the involuntary muscles, 239; on the sensation of light excited by metallic substances, 240; on the conducting and non-conducting powers of different substances, 241; results in regard to vitality from galvanic experiments, 290; method of applying it in deafness, ibid.; account of an improved apparatus for its effectual application, 291; comparison between galvanism and electricity, ibid.; how to apply it in suspended animation, ibid.; fibre of the blood, how affected by it, ibid.; experiments performed in London on a malefactor by Signor Aldini, 382; new apparatus for experiments, 390; state of it in Italy, 436; observations on the effects produced by it on the body of a malefactor, 445; new facts discovered, 487; on the causes of the irritability and excitability, x. 25; on the identity of electricity and galvanism, 26; an historic statement of the discovery, 53; on the term galvanic stimulus, 55; medical uses of galvanism further developed, 58; three successful cases of it in paralysis, 60; in the auditory organs, 61; oxydes formed in the metallic disks of the galvanic pile do not impede its action, 63; observations on its effects in irritability and excitability, 252; on the application of it for medical purposes, 330; comparison between it and electricity, 331; experiments at the hospital of Bamberg, 332; experiments by Mr. Carpue on the body of a malefactor, 383; another case, 576; experiments on a beheaded criminal, 94; cases of its alleged efficacy in hydrophobia, 191; historical sketch of the discovery, 524; its power in hydrophobia called in question, xii. 387, 513; xiii. 158; review of Elements of Galvanism in Theory and Practice, by C. H. Wilkinson, xiii. 82, 371; anecdote of the discovery, 84; gold leaf reduced to powder by it, 372; description of a galvanometer, ibid.; account of practical experiments in it, 373; its efficacy in paralysis, 374; in stiffness of the joints, 375; in asphyxia, ibid.; its success in epilepsy, xiv. 527; two cases of gutta serena relieved by it, xv. 151, 152; ineffectual in decomposing the muriatic acid, 294; applied to distinguish real from apparent death, 295; attempts to decompose water by, xiv. 271; augmentation of the galvanic action ascertained various ways, particularly through smoke, xv. 487; on the properties of different substances employed as galvanic conductors, ibid.; on the application of it in the cure of certain diseases, xvi. 472; its effects upon vegetable infusions, 479; on the application of it in the cure of diseases, xvii. 190; experimental observations on distilled water, 295; some account of a new theory on galvanic electricity founded on experiments, xviii. 245; its efficacy in paralytic complaints, 279; account of the experiments for decomposing the alkalies, xix. 93; discovery of important chemical agencies by, 190; repetition of the experiments on the alkalies, 286; discovery of the decomposition of the alkalies more amply detailed, xx. 95; description of the grand galvanic battery used at the Royal Institution for the decomposition of the alkalies, with a plate, 254; experiments on the decomposition of the earths, 383; account of a practical treatise on, 477; how to pass the galvanic fluid through any part of the body, 475; on the smallest number of galvanic combinations to decompose fixed alkalies, xxi. 350; experiments on muscular contraction, 460; account of Professor Davy's new galvanic apparatus, 527; farther observations on the importance of his discoveries, xxii. 11; the animal secretions affected by the Voltaic apparatus, 174; on the medicinal effects of the galvanic influence, 313; analogy of galvanism and electricity, 314; experiments, 316; simplicity of the galvanic application, 319; its effect on defective organs of speech, 321; on paralysis, 322; in tetanus, ibid.; a valuable diagnostic in rheumatism, ibid.; in mental diseases, 323; on the decomposition of the fluoric and muriatic acid, xxiii. 292; confirmation of Professor Davy's inferences respecting the nature of the alkalies and earths, xxiv. 346; on some of the combinations of the oxymuriatic gas and oxygen, with their chemical relations to inflammable bodies, xxv. 83; proposed lectures in, 280; comparison of the relative powers and energies of the galvanic piles, 362; electricity and galvanism repulsive powers, xxvi. 324; the latter a modification of the former, ibid.; a stimulus to the living system, 325; its effect in exciting chemical affinities, xxvii. 403; importance of the discoveries of Professor Davy, xxviii. 327; experiments on the spontaneous production of ammonia by, xxx. 260; description of the large galvanic battery of Mr. Children, 426; thick platinum wire ignited by it, ibid.; on the volatility of the metal, cerium, 427; experiments on the combustion of the diamond and other carbonaceous substances, xxxiii. 121; the principles of galvanism and electricity said to be different, 337; analogy between galvanism and nervous energy, 351; on the effects of a large galvanic battery, xxxiv. 165; iron converted into steel, and diamond powder diffused by it, 166; fusion of icidium effected by it, ibid.

GAMAGE, Dr. William, on the effects of carbonate of iron in ulcerated uterus, xxix. 14, 332; cases of cramp in the stomach relieved by venesection, xxxiii. 28.

GAMBOGE, not found in the Koulfam Pauli, xvi. 79; natural history of the, xxii 360; beneficial in hydrothorax, xxiii. 335.

GANGANELLI, Pope, remarkable instance of his equanimity, ii. 284.

GANGES, causes of the unhealthiness of the settlements on the banks of the, xix. 114.

GANGLIA, on those of the nerves, iii. 260; on their treatment by escharotics, xxiii. 432.

GANGRENE, account of a new method of treating it with musk and volatile salt, i. 501; that discovery not made by Mr. White, ii. 13; treated in the 16th century by the actual cautery, 278; on the claim of Mr. White to the musk treatment, 342; gastric juice beneficial in, iv. 167; in the kidneys, the consequence of calculi, v. 285; in sore-throat, cured by capsicum, 425; good effects of opium externally applied in, vi. 481; method of preventing it in compound fractures, vii. 307; on that of the arteries, ix. 88; cases of, x. 275; inefficacy of bark in, xi. 297; checked by nitre, xii. 488; farther remark on that remedy, xiii. 324; cases of white gangrene terminating fatally, 387; on the efficacy of nitre in, xv. 504; xvi. 114; case of stricture of the urethra followed by, xvii. 189; the germander a good fomentation in, 468; benefit of erysimum alliania in, xviii. 365; use of blisters in, 443; on that of the bladder, 519; case of crural hernia terminating in, xix. 501; Glauber's salt formerly used in, xx. 55; description of erysipelatous, 376; amputation not necessary in that of gun-shot wounds, xxiii. 456; produced by disproportionate action of cold and heat, xxiv. 21; follows torpor of the limbs in gun-shot wounds, xxx. 379; amputation when necessary in such cases, 462; that operation indispensable in particular instances, 464; case of stricture followed by gangrene of the arm, xxxi. 398; frequently attacks ulcerated buboes, 412; the muriate of ammonia successfully used in, 518; efficacy of opium in the dry gangrene of the toes, xxxiii. 430; a peculiar species of gangrene which attacks patients in hospitals, xxxiii. 399; that of hospitals affected by the state of the atmosphere, xxxiv. 394; on the nature of the disease and its symptoms, 395; process of the ulceration, 397; a febrile disease, 398; how affected by the atmosphere, 399; causes of the malady, 400; contagious, 401; particulars of cases, 402, 404, 405; different degrees of its violence, 467; on the state and composition of the atmosphere immediately producing it, 468; mean proportion of the parts of the atmosphere in hospitals, 469; sulphurated hydrogen in the same, 471; how to correct it, 473; conclusions from the experiments, 476; the effect of local contagion, 524.

GANOO, a disorder in India similar to the cretinism of the Alps, xxvii. 382.

GAOL FEVER, its rare occurrence, iv. 356; description of, 357.

GAPPER, E. P. on the external use of opium in hiccup, vi. 484; his case of the efficacy of digitalis in acute rheumatism, vii. 155; his case of hydrocephalus internus which terminated successfully, xv. 380.

GARAT, hereditary peculiarity in the family of, xxi. 329.

———, M. his description of the pyramids of Egypt, xxxiii. 392.

GARDEN, botanical one at Ghent, plants there, xii. 192; account of the Elgin botanic garden near New York, xx. 18; on that of Charlestown, South Carolina, xxi. 398.

———, Dr. on the virtue of spigelia marilandica as a vermifuge, viii. 428.

GARDENS, catalogue of plants, indigenous and exotic, in the Elgin garden at New York, xxvi. 162; account of the botanical gardens at Rome, 163; particulars of that at Chelsea, 294; anecdotes relative to some old ones near London, 470.

GARDINER, Edmund, an old practitioner in England, on the use of tobacco, xxv. 224, 230.

GARDNER, Lord, his excellent regulations for the benefit of seamen, i. 95.

GARDNER, Mr. his evidence on the claim of Dr. Jenner before the House of Commons, viii. 19; his practice of vaccination, xiii. 251; on the effects produced by galvanism upon vegetable infusions, xvi. 479.

GARENGEOT, M. his observations on the discovery of the circulation of the blood, ii. 232; on the operation for crural hernia, xi. 58; his account of the restoration of a lost nose, xxxii. 411.

GARGLES, benefit of the muriatic acid in fever, vi. 87; the oak-bark a serviceable one, xv. 69; the sloe efficacious in tumefactions of the tonsils, xvi. 169; excellent use of the root of cinquefoil, 265.

GARIDEL, M. his case of the fatal effects of colchicum in ague, xxxii. 406.

GARLICK, recommended by Hippocrates in inflammation of the lungs, xii. 470; botanical description of the several species of, xiv. 64; a good remedy for the gravel, 65; description of a peculiar species of, xxvi. 513.

GARNET, the island of Ammitok, on the coast of Labrador consists of, xxviii. 73.

GARNETT, Dr. Thomas, his analysis of the Harrogate waters, ii. 35; experiments on the mineral waters of Moffat, 354; his galvanic experiments at the Royal Institution, iv. 115; his cases of the efficacy of cold water in fever, v. 12; his case of apoplexia hydrocephalica, 121; remarks on that case, 124; observations on his case of apoplexia hydrocephalica, 344; his case of poison by arsenic externally applied, 542; his farther analysis of the waters of Harrogate, vi. 353; analysis of his lectures on Zoonomia, vii. 94; prevented from delivering the oration before the Medical Society, 297; his death and character, viii. 95; announcement of the publication of his lectures, 383; account of the progress of the publication, xii. 379; on hydrocephalus internus, xiv. 45; account of his Zoonomia, 96; his observations on the Brunonian system, xvi. 182; on excitability, 184.

GARRETT, John, his notes of a case of extensive wound, and singular recovery, xxxiv. 65.

GARRISONS, plan of an hospital for them, with an engraving, i. 460.

GARTHSHORE, Dr. Maxwell, his practice in arm-presentation, vii. 482; his evidence before the House of Commons in favour of the Fever Institution, xii. 163; on erysipelas in children, xx. 375; biographical account of, xxviii. 42; origin of the family, 43; educated under Cullen, ibid.; his inaugural thesis on opium, ibid.; his patronage of Dr. Pulteney, 44; settles in London, and becomes a member of the College, ibid.; and of the Royal and Antiquarian Societies, ibid.; his medical communications, 45; simplicity of his practice, 46; his love of science, 47; his Christian character, 48; the fortune which he acquired over-rated, 49.

GAS, the imperfect state of our physiological knowledge of the operation of different ones, i. 61; pretended combination of phosphorus in the azotic gas, 86; the application of the gases in diseases, 144; experiments on the oxygenous, 168, 170; on the action of different ones, 171; their use in purifying the air of sick chambers, 245; effect of phosphorus in pure nitrous gas, 300; method of rendering phosphorus luminous in that of azote, ii. 389; effects produced by dephlogisticated nitrous gas, 488; peculiarity of oxydated azotic gas, iii. 91; introduction to the knowledge of gaseous bodies, 384; on the germination of seeds in several kinds of, iv. 84; process of extricating the gases by galvanism, 119, 126; on the composition and solubility of the different kinds of, 265; on the respiration of them, 274; experiments to ascertain the effects of respiring some of the most deleterious, 360, 451; on the nature of coal when impregnated by different kinds of, 469; how to ascertain the quantity of gas in vegetables, v. 468; rotten wood rendered luminous by contact with gas, 583; hepatic gas used in mercurial gout, vi. 92; method of impregnating water with gas to create mineral waters, 185; oxygen and hydrogen separated from water by galvanism, 211; on the influence of light on phosphorus in them, 374; use of nitrous gas as an eudiometer, 376; carbonic gas the basis of mineral waters, 414; deleterious effects of one evolved by rhus radicans, 272; two in the atmosphere of marshes described, 493; azotic gas neutralized in that atmosphere, 496; the carbonic destroys the nervous power, ibid.; farther observations on those of marshy atmospheres, vii. 114; that called the gaseous oxyd of septar denied, 318; on the action of phosphorus in different ones, 374; in oxygen, 375; in the irrespirable, 376; effects of different ones on the germination of grain, 470; observations on their medical uses, 569; effects of the carbonous on the animal economy, viii. 189; effects of the same on the colour of the blood, ibid.; description of an instrument for the inspiration of gas, 382; on the effects of them in producing animal heat, 504; fatal experiment on hydrogenous sulphurated gas, 522; experiments on the putrefaction of flesh in different gases, ix. 219; the effluvia of febrile animal bodies produce peculiar gases, x. 462; analysis of the arseniated hydrogen gas, xi. 422; method of obtaining it, ibid.; physical properties of it, 423; chemical properties, 424; its habitudes to metallic solutions, 429, 430; whether the perspirable matter of drunkards has a peculiar gas, xii. 87; poisonous one of the Grotto del Cane, near Naples, xiii. 108; on gases that mix with the atmosphere, ibid.; deleterious ones issue from the sea, 109; the mineral gas employed to counteract contagion, xiv. 573; salivation produced by oxygenous gas, xv. 274; on the discovery of the anti-contagious power of mineral gas, 288; deleterious ones in oil-cisterns, 391; quantity of it discharged from the stomach in yellow fever, xiv. 213; effects of carbonic acid gas in the gravel, xvi. 227; effects of oxygen in restoring suspended animation, 529; experiments on the gaseous oxide of azote at Toulouse, xvii. 51; sudden death occasioned by the inspiration of nitrous, 188; noxious and fatal effects of septic gas in a family near the place of its evolution, 225; abstraction of oxygen from the atmosphere of marshes the cause of agues, 447; soporific effects of one emitted by saffron, xviii. 288; efficacy of carbonic gas in mortification, 536; presence of oxygen gas essential to animal life, xix. 372; result of experiments with the mineral acid ones to ascertain their prophylactic properties, xx. 35, *note*; hepatic diseases occasioned by deleterious gases, 345; experiments on atmospheric air and oxygen gas, xxii. 48; different ones which enter into the formation of the atmosphere, 177; on the galvanic production of different gases, 316; experiments in France for the decomposition of the acids, xxiii. 292; effects of carbonic-acid gas on the colour of seeds, 350; spontaneous phosphorescence produced in different gases, 389; on the effects of different ones injected into the blood-vessels, 455; on the combustion of charcoal in different kinds of, xxiv. 347; on some of the combinations of the oxymuriatic gas and oxygen, xxv. 83; gray oxide of tin decomposed by muriatic acid gas, 84; new name of chlorine proposed instead of oxymuriatic gas, 180; on a gaseous combination of oxymuriatic gas and oxygen, 361; fatal effects of the irrespirable gases of a coal fire, xxvi. 162; effect of different gases injected into the blood-vessels, 252; experiments on the combination of oxymuriatic and oxygen gas, 298; prize question on the specific heat of, 345; effects of an injection of different gases into the venous system, 406; on the production of carbonic-acid gas in respiration, xxvii. 9; detail of the experiments on the combination of oxymuriatic gas and oxygen, 15; effect of the solar rays on gaseous oxide of carbon and oxymuriatic gas, 340; combinations of certain metals with chlorine and oxygen, ibid.; gases produced in experiments with arsenic, 519; experiments on silicated fluoric, xxviii. 157; hydrophosphoric gas produced by the union of phosphorus and hydrogen, ibid.; on a gaseous compound of carbonic oxide and chlorine, 195; abstract of papers in the Philosophical Transactions on the gases, 328; on the process of gassification, 335, 414; on the gravity or levity of gases, 417; water considered as a gas, xxix. 20; history of the discovery of hydrogen gas, 400; and that of carbonic-acid gas, ibid.; experiments on the same, 401; on nitrous gas as a test of oxygen in common air, 403; on that evolved by liquid gum and alcohol, 480; description of a glass apparatus for condensing gases, 507; determination of the capacity of oxygen gas, carbonic-acid gas, and hydrogen gas, for heat, 514; farther experiments on the gases for the same object, 515; defiant gas contains no oxygen, 516; hydrogen and carbon combine in two proportions, ibid.; decomposition of gas by electricity, ibid.; on ammoniacal gas, ibid.; on nitrous gas, 517; porous bodies absorb gases in different proportions, ibid.; the nitric acid resolved into gas, 524; on the gas produced by sulphur, xxx. 79; invention for preventing the explosion of gas in coal-mines, 82; experiments on hydrophosphoric gas, 83; unknown inflammable one in azote, 85; on the hydrogen gas evolved by hydrosulphurat, 256; on the determination of the specific heat of the different gases, 399; table of the specific heat of the same, 402; chlorine and phosgene gases unaffected by galvanic experiments, 426; instance of combustion during the heating of barytes with muriatic acid gas, 523; new gas discovered in saltpetre, xxxi. 255; beneficial effects of oxygen gas in restoring suspended animation, xxxii. 36; effects of breathing artificial gases upon the trachea, 49; method of preventing inhaling impure gas, 80; experimental observations on the respirable gases, 132; experiments on the preservation of animal flesh in the gases, 435; on the hydrionic-acid gas and its compounds, xxxiii. 41; on the action of some compound gases in iodine, 46; combustion of the diamond in oxygen gas, 121; announcement of a treatise on the nature and advantages of gas-light for illuminating streets and houses, 252; death of Gehlen by inhaling arseniated hydrogen gas, xxxiv. 458.

GASILOTRE, an apparatus for producing artificial mineral waters, described, i. 365.

GASSNER, Dr. on the virtues of opium in the bite of a snake, xiv. 524.

GASTRIC JUICE, the chyle a solution of vegetable and animal substances in the, ii. 80; observations on its use in disorders of the stomach, 467; its influence denied, 468; that of an owl externally applied in abdominal swellings, iii. 168; its beneficial application to sores, iv. 167; chyle formed in it by the action of saliva, xvi. 200; on the dissolution of the stomach by the, xxi. 347; enumeration of farinaceous substances which become soluble in, xxii. 403; on the digestive powers of it, ibid.; the secretion of it diminished by a division of the eighth pair of nerves, xxvii. 10; on the opinion of Spallanzani respecting the action of this fluid on food, xxx. 517; proved to resemble saliva, ibid.; putrefies in a similar manner, ibid.; its acidity not essential, ibid.; efficacy of magnesia in correcting it, ibid.; observations on its effects in digestion, xxxii. 257; putrefies like saliva, ibid.; on its uses, xxxiii. 355; proved to be only saliva, 421; therefore not a solvent of itself, ibid.; causes of its acidity, ibid.; on the change of aliments in it, ibid.; the separate purposes of saliva and gastric juice, ibid.; on the nature of digestion, 422; how worms exist in it, xxxiv. 61.

GASTRITIS, on the benefit of glysters in, iii. 229; history of a fatal case of, xvi. 536; efficacy of aromatics in, xvii. 159; defence of the former case, xviii. 60, 61; on the analogy between it and hydrophobia, xxi. 276; effects of steam-bathing in, xxiv. 162; farther remarks on the affinity between hydrophobia and gastritis, xxix. 12; additional observations on the same, 42; the priority of Mr. Barrett to that discovery asserted, 454; explanatory account of Dr. Kinglake on that subject, xxx. 96; Dr. Adams advances a claim to that discovery, 201; ample refutation of his pretensions, xxxi. 1; enumeration of authors who have treated the two diseases as analogous, 6; explanation on the same, 281; dissimilarity of hydrophobia and, xxxiii. 15.

GASTROTOMY, case of intus susception cured by that operation, vii. 38; observations on that operation, xi. 118.

GASTRODYNIA, history of a fatal case of, iv. 227; appearances on dissection, 229; remarkable case of it in a lady, xii. 289; farther observations on that case, xiii. 132; objections to the term, 207; additional information on the last-mentioned case, xv. 216; another remarkable case of, xviii. 163; a natural consequence of gout, 168; the use of the word controverted, xxii. 264; oxyd of bismuth recommended in, xxxiii. 458; the efficacy of that remedy fully proved, xxxiv. 175.

GATINAIS, remarkable effects of the odour of saffron observed in that country, xx. 15.

GATTAKER, Mr. on the internal use of solanum, xii. 136.

GATTI, M. his case of secondary small-pox, xiv. 176.

GATTONI, M. his experimental observations on eudiometers, i. 397.

GAUD, prize question to the Medico-Chirurgical Society of, xxxiii. 71.

GAULTIER, M. his narrative of the poisoning of one hundred and eighty persons by the berries of belladonna, xxxii. 390.

GAVARD, Hyacinthe, account of his treatise on myology, i. 306.

GAUBIUS, the effects of the mineral gas as a preservative against contagion known to, xiv. 573.

GAYLENREUTH, in Germany, fossil remains in the cave of, xxvii. 26.

GEBAL, Dr. on the virtue of aconite in rheumatic cardialgy, iv. 174.

GEDDES, Dr. on the influenza, x. 291.

GEESE, on the white serum in the blood of, xxv. 155.

GEHLEN, Professor, account of his medical and physical dictionary, i. 90; on the present state of the knowledge of opium, xii. 38, 117; his observations on the essential oil of turpentine, xxiv. 322; confirmation of his opinion, 324; his melancholy death, xxxiv. 438.

GELATINE, gelatinous substance formed from the muscular fibres of frogs, ii. 298; observations on, ix. 499; on its use in intermittent fever, x. 323; experiments to prove that cinchona does not contain it, xi. 266; soluble in water, 269; its benefit in intermittents proved, with the form of its use, xii. 570; peculiar properties of, xiv. 266; formed of the solid parts of animal bodies, xvi. 304; quantity supplied by the blood, 453; resemblance between it and gum, 266; substituted for Peruvian bark in intermittents, xviii. 34; that of the lichen a new species of gum, xix. 46; experimental observations on the gelatine of the blood, 74; farther account of the same, xxiv. 10; may unite with acids, 327; not discoverable in dropsical fluids, xxviii. 62.

GELHARD, J. N. his observations on the time of flowering of vernal plants, ii. 491.

GENERATION, on the theory of, ii. 152; observations on the theory of, 243, 321; analogy between that of animals and that of plants, 243; observations on Dr. Darwin's opinion on, 277; remarkable structure of the organs of, 305; examination of the organs subservient to, 321; experimental results, 323; on the organs of it in the Julus Camplanatus, 481; on the nutriment afforded by those animals which have their organs perfect, iii. 68; remarks on a peculiar theory of, 139; account of the anatomical discovery of the organs of, 156; on the female organs, 157; whether they secrete semen, 159; on the opinion that male and female children are conceived differently, 160; on the supposed existence of the allantois, 161; on the evolution of the embryo, ibid.; description of a new theory on, v. 405; accounted for by the absorption of the semen in the vagina, ibid.; oil beneficial in diseases of the organs of, vi. 72; observations on various theories of, 254; odours exhaled from the organs of, 441; remarkable conformation of the organs of, xiii. 179; anatomical observations on the formation of the human ovum, xv. 271; cases of diseases of the organs of, xx. 429; benefit of cantharides in those complaints, 432; cases of the principal diseases of the female organs of, xxi. 29, 52; additional observations on diseases of the generative organs, 303, 371; prevalence of those diseases, 519; farther remarks on those diseases, xxii. 17, 126; embarrassing circumstances in female disorders of the, 46; extirpation of a diseased testicle in an advanced stage, xxiii. 59; case of the successful use of cantharides in debility of the organs of, 311; commencement of a work on disorders of the organs of, 440; remarkable eruptive disease on the penis, with a plate, 441; on a derangement in the female organs, 519; on the discovery of a membranous fence at the aperture of the urethra, xxv. 41; case of too small a perforation of the glans penis, 42; explanation on the same subject, 118; practical observations on the sole-rocele and other morbid derangements of the testicle, 175, 265; case of extraordinary malformation of the organs of, 441; on the generative organs of the leech, xxvi. 413; on those of the opossum, 422; observations in support of equivocal generation, 454; objections to the hypothesis of equivocal generation, xxvii. 50; review of a treatise on diseases of the generative system, 139; pathology of the female organs of, 141; on diseases of debility, 145; account of different hypotheses on the subject of, xxviii. 324; case of diseased testicle, xxx. 164; observations on peculiar diseases incident to the sexes, xxxi. 210; on the causes of priapism, illustrated by a diagram, 211; phenomena of erection, 212; on the sexual connexion, 213; on the vascularity of the female organs, 214; remarkable change of sex among a whole people, xxxii. 166; on Leuwenhoeck's theory, 209; signs of an immature child, 330; on monstrosity, 331; on the first rudiments of the human body, 332; on hermaphrodites, ibid.; when a fœtus may be supposed to live, 333; on the seat of the soul, 334; on suppositious births, ibid.; on superfœtation, 335; the process of generation similar to creation, xxxiii. 155; on that of the lamprey, xxxiv. 165; remarkable description of an hermaphrodite, 525.

GENISTA, or dyer's green wood, botanical description of, xix. 75; its uses in medicine and dyeing, ibid.

GENITALS, oil beneficial in morbific irritation of them, vi. 72; remarkable conformation of the organs of them in a female child, xiii. 179; singular case of malformation in the, xix. 180; cases of diseased, xx. 429; extirpation of a diseased testicle in an advanced stage, xxiii. 59; on herpes of the prepuce, 441; observations on morbid derangements of the, xxv. 175, 265, 350; case of morbid enlargement of the clitoris, 236; description of the sarcocele of Egypt, with cases, 289, 294; on the acute hydrocele, 351; on the spurious hydrocele, 353; chronic hydrocele of the tunica vaginalis testis, 354; case of singular and complicated malconformation of the genital organs, 441; on herpes of the prepuce, xxvi. 195; review of a treatise on the diseases of the, xxvii. 139; case of diseased testicle, accompanied with that of the lungs and brain, xxx. 164; inflammations of the penis erysipelatous, xxxi. 42; on the causes of priapism in the human species, illustrated by an engraving, 211; on the female organs, 213; change of sex among a tribe of Tartars, xxxii. 166; an interesting work announced on disorders of the, xxxiii. 516; singular account of an hermaphrodite, xxxiv. 525.

GENT, General, his introduction of the root of the datura as a remedy in asthma, xxvi. 22, 48.

GENEVA, encouragement of vaccination at, v. 386; the clergy evince their zeal in the same cause, vi. 281; small-pox subdued there, xii. 400.

GENTIAN, description of a poisonous root discovered among, xxix. 107; the cultivation of that plant recommended, ibid.

GENTIANA, *(chiragata,)* a valuable indigenous plant in the East Indies, xxvi. 30; description of the *gentiana seplanfida*, and the *gentiana macrophylle*, 514.

GEOFFREY, M. his observations on the origin of ambergris, xi. 168; recommends the use of hemella as anodyne and vulnerary, xx. 272; on the properties of jalap, xxi. 393; his observations on the different species of bats, xxx. 429; on the heads of oviparous animals, 432; on the sternum of birds, 518; his observations on the effects of colchicum, xxxii. 205.

GEOGRAPHY, outlines of a system of medical, i. 254; observations towards a work of that description, xx. 3; on local peculiarities, natural history, and state of medical practice, 4; additional facts on the same subject, xxii. 106; disappearance of the island of Bossen, in Africa, xxiii. 263; farther particulars of the same, 526; observations on the advantages of a knowledge of, xxvi. 9; important information derived and afforded by the travels of Humboldt in South America, 10, 15; new island discovered in the Icy Ocean, xxvii. 77; a new volcanic island in the Western Ocean, 80; description of Iceland and the Westmenn Islands, 118, 124; narrative of an eruption of a volcano in the sea, xxviii. 200; abstract of works on, 326; observations on the diseases of the Laplanders, xxxi. 51; on the mean temperature of Stockholm and Petersburgh, 54; small quantity of rain in Sweden, ibid.

GEOLOGY, account of an American society for promoting that science, i. 86; new variety of iron-ore found in America, 198; inquiry into the strata of England, 254; description of remarkable caverns, ii. 492; on the gold and silver mines of Transylvania, iii. 284; account of fossils found in Sendomir, ibid.; on the formations of the rocks of the Alps, 375; account of a work on the structure of the Alps, xxiii. 525; curious geological facts in the mineralogy of Iceland, xxv. 362; on the geognostic relations of the rocks on the Isle of Arran, 453; extraordinary heights of the mountains in South America, xxvi. 11; on the rocks in the environs of Edinburgh, 168; on the geognostic relations of the Iceland crystal, ibid.; on the fossil remains discovered in various countries, xxvii. 24; account of the first volume of the Transactions of the Geological Society in England, 28; volcanic structure of the Westmann-Eyar Islands, 122; general meeting of the Geological Society in 1812, 340; analysis of papers read before that Society, xxviii. 73; on the geology of Labrador, ibid.; description of the fractures and fissures in the surface of the earth, 75; on metallic veins, 76; on the Wernerian system, ibid.; state of metallurgy, 77; on the stratified rocks containing salt and coral, 157; dreadful convulsions of the earth in South America, 161; phenomenon in the island of St. Vincent, 162; extraordinary darkness at Barbadoes, ibid.; an eruption of a volcano in the sea, off the island of St. Michael, 200; proceedings of the Geological Society, 235; on vegetable fossils, 236; on the mineralogy of St. David's Head, South Wales, ibid.; account of the Isle of Teneriffe, ibid.; account of new varieties of alcyonia in the Isle of Wight, 237; on fossil remains from Sicily, 238; on the geological structure of Barbadoes, 239; tubes found in the sands in Lancashire, ibid.; on the vitrified Fort of Dun Mac Suoichain, near Oban, Argyleshire, in Scotland, 240, 241; vein of copper-ore found in Nottinghamshire, 242; particulars of the phenomenon at Barbadoes, 251; deductions from observations on fossils, 304; on the process of petrifaction, 305; abstract of, 326; mean density of the earth, xxix. 407; account of the eruption in the island of St. Vincent, 426, 427; mineralogical description of the Ochil hills in Scotland, 429; observations on the structure of the rocks in Italy, 431; account of fossils found in Dorsetshire and Wiltshire, 511; head of a whale discovered at Antwerp, 519; jaw-bone of a rhinoceros at Abberville, 520; observations on beds of sea-shells, ibid.; account of

M. Cuvier's work on fossil bones, ibid.; analysis of a course of lectures in, xxx. 349; on the middle granite district of Galway, xxxii. 75; on overlying primitive formations, 76; on the rocks in Galway, ibid.; on an extensive bed of magnesia lime-stone near Dublin, 77.

GEOLOGICAL SOCIETY, instituted in Cornwall, xxxiii. 337.

GEOHEGAN, his application of cold in the treatment of hernia recommended, v. 116.

GEONHEGAN, Edward, his strictures on the customary practice in strangulated hernia, iv. 317; his observations on the nature and treatment of some exasperated symptoms of the venereal disease, vi. 180; appendix to that article, xii. 279; on the irritability of mercury in the cure of bubo, xiii. 435; review of his Commentary on the treatment of ruptures, particularly in a state of strangulation, xxiv. 512; his case of strangulated hernia, xxvii. 162; review of his Commentaries on the treatment of the Venereal Disease, particularly in its exasperated state, xxxi. 408, 495.

GEORGE THE SECOND, particulars observed in the dissection of, xxxiv. 145.

GEORGE'S HOSPITAL, ST. on the election of a surgeon for, iii. 486; lectures at, xiv. 286; xviii. 387; report of the committee appointed to examine the laws relative to the surgeon's pupils at, xxviii. 311; lectures at, xxx. 350.

GERANIUM, an odour resembling that of castoreum exhaled by various kinds of, vi. 346.

GERARD, John, his efficacious use of tobacco in the cure of wounds in the sixteenth century, xxv. 221; his case of dropsy cured by the same remedy, 234.

———, J. N. his comparative tables of the anatomy of domestic animals used in agriculture, ii. 94.

———, Dr. his evidence on a trial for murder, xxi. 339; his case of amaurosis successfully treated with Cayenne pepper, xxii. 353.

GERMANDER, *(Anjuga Chamæpithys)*, botanical description and medicinal virtues of the, xvii. 466; benefit of the common germander in gout, 467.

GERMANY, encouragement of English literature in that country, i. 88; medical works published there, 89; on the medical schools in different parts of, 177; view of the literature of, 402; new medical publications in, ii. 95, 199, 304, 499; state of its literature, 396, 400; raw flesh ate by the ancient inhabitants of, iii. 67; new medical works in, 96, 192, 280, 492, 575, 588; iv. 188, 365, 475; success of vaccination in, v. 352; institution for infantile diseases at Vienna, 586; progress of vaccination in, vi. 280; vii. 171; viii. 193; successful application of galvanism in, x. 330; on the number of empirics in, xii. 423; progress of vaccination in, xiv. 26, 235; decrease of the small-pox in, 382; the elephantiasis rare in that country, and why, xv. 79; practice of the fowlers in, xvi. 169; state of medical science in, xxiii. 38; method of making sour-crout in, 367; on the present state of medical knowledge in, xix. 271; successful method of treating tetanus in the military hospitals of, xx. 8; fetid vapour in, xxiii. 283; plague and leprosy in, 284; a mortal disease among cattle in, 285; prize questions offered by the Austrian government, 352; number of books sold at Leipsic fair, xxv. 283; animal remains in the caverns of, xxvii. 24; some account of the universities in, 385; catalogue of medical books published in, at the Leipsic Michaelmas fair in 1813, xxxi. 312.

GERMINATION, experiments on that of garden cresses in different gases, iv. 84; effects of different gases on that of several kinds of grain, vi. 470; experiments on the descent of the roots and the elongation of the germs of plants, xv. 294; on the changes induced on atmospheric air by the germination of seeds, xix. 371; farther observations on the same, xx. 6; oxygen the principal stimulus in, xxii. 109; experiments on the manner in which plants shoot forth their radicles, xxv. 452; account of microscopical observations and experiments on the process of, xxvi. 170; importance of the stamina and pistilla in ripening the seeds of plants, xxvii. 310; a living power said to be the germinating principle in plants, xxviii. 332; observations on some peculiarities in seeds, xxix. 509; experimental observations on the ascent of the sap in vegetables, xxx. 251; researches into the structure of the organs of fructification, 253, 254.

GERVIS, Mr. his singular instance of fatality in a family, xxviii. 23.

GESENIUS, Dr. on the use of arnica montana in gutta serena, i. 27; on hot flour as a remedy in erysipelas, 154; recommends misletoe in epilepsy, 180; on the internal use of phosphorus, 283.

GESNER, Conrad, his acquaintance with tobacco, xxiv. 449.

GESTATION, practical experiments on it in animals, i. 46; artificially produced, 47; necessity of observing the several periods of it, v. 47; case of ovarian, ix. 56; advice in regard to the exercise of, xiv. 180; observations on extra-uterine, xxiv. 406; salutary effects of the exercise of, 491; case of hernia umbilicalis operated on with success during utero, xxviii. 13; three queries, 1. On the earliest period of gestation for a child's being born alive.—2. What is the latest period of gestation for a living child?—3. The usual period of gestation for cows, 432; curious remark on that of the viper, xxx. 518; a child retained in the womb fifty-two years, xxxiii. 65, 147.

GEUM URBANUM, or common avens, botanical description of, xvi. 267; a good febrifuge, ibid.; description of the water avens, 557; beneficial in agues and diarrhœas, ibid.; the roots mixed with milk efficacious in fluxes, xviii. 436; used in brewing good beer, xxvi. 345.

GEYSER, one of the hot-springs of Iceland, described, xxv. 362; analysis of its waters, xxxiv. 42.

GHENT, account of plants cultivated in the botanical garden at, xii. 192.

GHINUS, Lucas, the first professor of botany in Europe, xxvi. 163.

GIBBES, Dr. G. S. review of his treatise on the Bath waters, iv. 457; account of his second treatise on the same subject, x. 183; his discovery of an extraordinary portion of iron in those waters, xvi. 383, 484; his case of the fatal effects of stramonium in asthma, xxvi. 49; his analysis of the Melksham chalybeate, xxx. 299, 383.

GIBBON, James, his call upon Dr. Yeats for an account of a case of Ischuria, xxx. 176; reply to him, with a defence of the case, 197; his state of the case, 355; refutation of his statement, 488; Anne Foulkes vindicated from his charges, xxxi. 19; his answer to Dr. Yeats, 21; confutation of his opinions on ischuria, 111; his conduct exposed, 121; his farther observations on the case of Anne Foulkes, 125; review of the same, 205, 283; animadversions on his conduct in the dispute, 359.

———, Edward, his remark on scepticism, xiii. 150; his observation on the ancient baths, xvi. 282; on the studies pursued in the English Universities, xxxii. 325; his extraordinary powers of conversation, xxxiv. 207.

GIBBONS, Dr. Thomas, review of his medical cases, iii. 94.

GIBNEY, Dr. John, his account of the influenza at Navan, in Ireland, x. 527; review of his practical observations on cold and warm bathing in scrofulous and gouty complaints, xxx. 224.

GIBRALTAR, report on the malignant fever at, xiii. 193; cases of the disease, 195; farther account of that disorder, xiv. 137; symptoms of the fever there in 1804, 200; the treatment used in that disorder, 202; observations on the epidemic, xviii. 8; the fever proved to be contagious, xxi. 249; fossil bones found in the rock of, xxvi. 18; observations on the ardent fever at, xxix. 414; historical account of fever there in 1810, 1811, 1812, and 1813, xxxi. 489; observations on the malignant fever in that garrison, xxxii. 46; account of the epidemic which occurred there in 1804, 1810, and 1813, xxxiii. 414; answers to queries relative to the epidemic in that garrison, by R. Amial, xxxiv. 301, 309; proofs of the epidemic being the yellow-fever, 336; on the hot winds at, 407; reports on the diseases at, 498, 505.

GIBSON, Dr. review of his Treatise on Bilious Diseases and Indigestion, ii. 194.

———, Benjamin, on the cause of the puriform ophthalmia in new-born children, xvii. 421; on the formation of artificial pupils, xxv. 535; on the use of the couching-needle in operating on infants, xxvii. 161; his death and character, 438; his treatment of the soft cataract, xxxiii. 418.

GIDDINESS, said to be serviceable in the cure of gutta serena, i. 25; produced by the nuts of the common beech, xvi. 74.

GIDELEY, Mr. his account of the influenza as it appeared at and about Penzance, in Cornwall, x. 294.

GIESE, Mr. on the proportion of benzoic acid in the urine of horses, viii. 476.

GIFFARD, Mr. his account of two kinds of the cow-pox, xv. 169.

GILBERT, M. his history of European plants, i. 210.

———, J. on the efficacy of digitalis, ix. 249.

———, Professor, his comparative view of modern medical theories, i. 360, 486; on puerperal fever, iii. 80, 159; on the use of gelatine in fevers, x. 323; account of his chemical lectures, xxvii. 387.

GILBY, Dr. his cases of diabetes mellitus, iv. 205; on the benefit of nitric acid in the same, 211.

GILCHRIST, Dr. on the slow or nervous fever, ii. 412; his opinion on the virtue of mercury in fever, xxix. 213.

GILEAD, description of the balm of, i. 33; historical notices of that plant, ibid.; used as a cosmetic in Egypt, 34; an important article of Eastern commerce, 35; marvellous stories of its virtues, ibid.; different species of the plant, 36; opposite accounts given of it by Alpinus and Bruce, 37; description of the plate, 39.

GILHAM, Mr. on his treatment of the wound of Sir Ralph Abercrombie, xii. 173, 243; his explanatory defence, 288.

GILIBUT, M. his observations on pemphigus, xxxii. 281, 283, 285.

GILL, Dr. his communication of vaccine matter at Madeira, ix. 310.

GILLESPIE, Dr. Leonard, on the venom of serpents in the West Indies, iv. 293; on the use of the aristolochia in such cases, 295; his report of the state of the hospital at Norman Cross, xii. 345; cases of surgery communicated by him, 514; his account of the plant Krameria Trianria, xiv. 127; his medical report of the state of the fleet under Lord Nelson, 413; account of regulations adopted for the health of seamen, 414; on the use of spider's-web in intermittents, xxi. 353; on an indigenous remedy for cancer, with a copy of the prescription, xxxii. 109.

GILLMAN, James, account of his prize dissertation on the bite of a rabid animal, with an engraving, xxvii. 236; his examination illustrated and confirmed by recent instances, xxviii. 16, 17.

GILLUM, Dr. on the efficacy of the inoculated small-pox as it affects population, xvi. 80.

GILPIN, Dr. on the contagious nature of yellow-fever, xxxii. 45; his account of the epidemic at Gibraltar, xxxiii. 414.

GILSON, A. his observations on the fever prevalent at Guzerat, with remarks on the action of mercury in the diseases of India, xxxiv. 243.

GIMBERNET, Antonio, his method of operating in crural hernia, xi. 123; the alleged novelty of his method disproved, 124; improvement on his plan, 125; his description of the crural arch, 280; case of strangulated hernia successfully treated by his method, xxiii. 379.

GIMBERNET, Charles, on the effects of nitrous gas in preventing contagion, xi. 136.

GIN, that of Holland, how made, xviii. 371.

GINGER, its virtues in gouty complaints, xii. 66; singular effects of it in rheumatic affections, xix. 527; an acid obtained from it, xxii. 349; account of the various species of it, xxix. 436.

GINGUENE, M. his memoir of M. Cabanis, xxxi. 400.

GINKLOFE, a peculiar disease among the Icelanders, described as similar to trismus, xxvii. 123.

GIRAFFE, or the Camelopardalis, known to the ancient Romans, xxvi. 17; a specimen of it in Bullock's Museum, ibid.

GIRARD, J. his Tableaux Comparatifs de l'Anatomie, reviewed, ii. 94.

———, Dr. account of his essay on the tetanus rabiensis, or inquiry into the accidents produced by rabid animals, xxiii. 41; observations on his opinions, 149.

GIRAUD, M. the pupil of Dessault, his character as a surgeon, i. 80.

———, J. T. his method of preserving vaccine virus, ix. 397; his singular case of monstrosity, with a plate, x. 171; excellence of his vaccinators, xx. 23.

GIRAULT, Mr. on the benefit of warm muriatic baths in gout, v. 687.

GIRDLESTONE, Dr. Thomas, his case of diabetes, with an historic sketch of that disease, ii. 87; on the occurrence of phymosis in diabetes, v. 65; his case of fracture of the occiput, vii. 53; on the prevalence of typhus, 54, 96; his reply to Dr. Langslow on a case of apoplexy, 149; letter addressed to him on that subject, 274; on the benefit of digitalis in hydrothorax, 401; on the use of mercury in dropsy, 402; singular history of cutaneous affection related by him, 508; on the danger of giving hot food to infants, xiii. 298; address to the inhabitants of Yarmouth on vaccination, xv. 92; on the effects of Fowler's solution in lepra and other diseases, 297; on tapping at the navel, xvii. 351; his cases of that practice, 352; disavowal of an article ascribed to him, xix. 576; reply to a wanton attack made upon him, xx. 135; his apology to, 266; his case of ischuria, xxxi. 111; his observations on the Mal d'Aleppo,

282; farther particulars on the same, 372.

GIRLS, on the improper education of, v. 298; more subject to chorea than boys, xiii. 408; case of a blue one, with appearances on dissection, xiv. 471; secret of the invisible one explained, xvii. 199.

GIRTANNER, Dr. ascribed the origin of syphilis to the bite of an insect, i. 232; on the compound nature of phosphorus, 300; on the composition of the acids, ii. 78; on the communion of oxygen to the solids by the blood, 202; difference between his theory and that of Dr. Beddoes, 308; on carbonic acid and atmospheric air, iv. 83; his death and character, 89; on difficult dentition, 174; his analysis of azote, 185; his claim to the discovery of the method of curing venereal diseases by oxygen, 469; analysis of the crystals of Switzerland, ibid.; on the animal origin of amber, vii. 92; asserted oxygen to be the principle of irritability, x. 25; on the history of syphilis, xiii. 89.

GIZZARDS, experimental observations on those of the cassowary and ostrich, xxix. 173.

GLAND, scirrhous parotid, removal of the, xxii. 221; treatment of the diseases of the prostate, xxix. 226.

GLANDS, prostate, on induration of the, iv. 506; those of the lungs, black matter contained in the, xxix. 506.

GLANDERS, in horses, experiments to excite, xxv. 281.

GLANDULAR SWELLINGS, successfully treated by the muriate of lime, xix. 364.

GLASGOW, regulations respecting degrees of medicine at the University of, xiv. 284.

GLASS OF LEAD, fraudulently substituted for glass of antimony, xxiii. 439.

GLAZIERS, cautions to those who have received the poison of lead, xxi. 526.

GLEET, on the internal use of cantharides in, xv. 415; xvi. 78; on its nature, and on the efficacy of cantharides in its treatment, xvii. 279; case of obstinate, 425; reply to, xviii. 155; the worst cases cured by cantharides, xxi. 519; obstinate case of, cured by seawater, xxxiv. 247.

GLOSSARY, Dr. Turton's medical, xii. 565.

GLOTTIS, two cases of the, completely closed up, xxii. 140.

GLYSTER, an obstruction of the œsophagus removed by a tobacco, xvii. 20.

GMELIN, Professor, his history of chemistry, v. 173.

GOAT-POCK INOCULATION, two trials of, by J. J. Heydeck, of Madrid, x. 339.

GODDARD, Mr. his remarks on fractures of the cranium, xiii. 526.

GODERNEAU, nostrum invented by, described, xxx. 222.

GODFREY, Robert, his work entitled "Various Injuries and Abuses in Chymical and Galenical Physick," xii. 427.

GOE, Mr. his successful case of divided trachea, viii. 162.

GOETS, M. of Paris, on the inutility and dangers of vaccine inoculation, ix. 484.

GOETTLING, his experiments on the combustion of phosphorus in azotic gas refuted, i. 85; his remarks on the origin of carbon, ii. 388; analysis of his manual of the theory and practice of chemistry, iii. 188.

GOITRES of the valley of Luchon, similar to the Cretins of the Valais, interesting account of the, xxxiii. 209; remarks on them, by Dr. Adams, 433.

GOLD, an oxyde and a muriate of, highly efficacious in syphilis, &c. xxvii. 32; memoir on these preparations, 156; their efficacy in syphilis confirmed, 158; confirmed by trials in London in cases of syphilis, xxix. 13.

GOLDHAGEN, Dr. his fatal attack of fever, xix. 171.

GOLDING, Mr. his case of parturition, in which the still-born infant was found to be affected by hydrocephalus internus, xxv. 205.

GOLDSCHMID, M. his account of the factitious mineral waters prepared by him, i. 363.

GOLDSON, Mr. his pamphlet on Small-pox subsequent to Vaccination, examined by Dr. Walker, xi. 505; xii. 44, 168; Mr. Goldson in reply, xii. 49; analysis of his pamphlet, 79; Mr. Ring in answer to Mr. Goldson, 181; his pamphlet examined by Dr. Macdonald, 298; by Mr. Thackaray, of Cambridge, 331; letter from the French Minister of the Interior on the subject of, 348; Mr. Hill on, in reference to Dr. Macdonald, 445; Mr. Ring on Dr. Macdonald's answer to Mr. Goldson, 535; Dr. Walker on Mr. Goldson's second pamphlet, xiii. 183; remarks on this pamphlet by the editors of the Medical and Physical Journal, 268; strictures of Mr. Ring on, 457.

GOMES, B. A. his essay on Cinchonin reviewed, xxvii. 295.

GONORRHŒA, on the cure of, v. 379; effects of lightning and electricity on, vi. 277; observations on, with the remarkable case of a female infant, xi. 452; artificially produced, for the relief of various diseases, supposed to depend on its cessation, xii. 519; Mr. Woodham's observations on the preceding cases, xiii. 150; on the existence of ulcers in the urethra, 79, 321, 548; diagnosis between this and leucorrhoa, xv. 616; the juice of houseleek (*sempervivum*) useful in, xvi. 76; probable connexion of, with purulent ophthalmy, xxiii. 78; on the great efficacy of opium and calomel in severe cases, xxv. 445; experiment on, causing a smooth superficial ulcer, xxxiv. 245.

GOOCH, Dr. his cases in which the smoking of stramonium afforded immediate relief in asthma and difficult breathing, xxvi. 55.

GOOD, Mr. John Mason, his extemporaneous oration before the Medical Society of London, xix. 381; on medical technology, xxv. 56.

GOODALL, Mr. his successful treatment of a severe case of hernia, xiv. 452; his two cases, showing the efficacy of oil of turpentine in the treatment of burns and scalds, xvi. 313; his case of vaccination, in which a vaccine pustule was formed on the thigh by the action of the absorbents, xx, 333.

GOODLAD, Mr. on purulent ophthalmy, with his particular mode of treatment, xxiii. 154; his observations on the treatment of scrofula, xxxiv. 65.

GOODWIN, Mr. on the efficacy of bark in parturition, iii. 442; his account of a case of tænia accidentally cured by camphorated spirit of wine, xxv. 23; his account of a female, naturally fair, who became perfectly black, 24.

———, Mr. of Earlsoham, his case and treatment of angina pectoris, vi. 320; on the influenza of 1803, ix. 509; his case of gravel, xv. 35; his observations on scarlatina anginosa maligna, xxiv. 465.

———, Mr. of Worksworth, on the influenza of 1803, x. 195.

GOOSE-GRASS, yellow, (*galium*,) its flowers prescribed by the French in hysteria and epilepsy, xi. 533.

GORDON, Dr. John, his experiments on the extrication of caloric during the coagulation of the blood, xxxii. 223.

GORDON, Dr. on the qualities and sensations of sounds, xxvi. 168.

GOREY, Dr. history of his case of hydrophobia, xviii. 475.

GORGET, invented by Mr. Abernethy, his remarks on the, ix. 393, 567; an improvement suggested, xxii. 207; Mr. Smith's observations on the cutting gorget, 224; Mr. Simmons's apology for the cutting gorget, in answer to Mr. Lawrence's observations on lithotomy, xxiii. 238, 339.—*See* LITHOTOMY,

GORHAM, Dr. on the vesicating properties of the potatoe-fly, xxiii. 323; his case of an organic disease of the heart, xxxii. 303; his observations on chronic diarrhœa from an ulcerated state of the intestines, 374.

GOTTINGEN, questions proposed by the Academy of, relative to yellow fever, x. 383.

GOUGH, Mr. his experiments and observations on domesticated dormice, xix. 383.

GOURLAY, Dr. William, on the successful progress of vaccination in Madeira, xxiii. 98; on the natural history, climate, and diseases, of Madeira, xxv. 425.

GOUT, medicated frictions recommended in, i. 72; connexion between that disorder and hypochondriasis in men, and hysteria in women, 147; benefit of the oil of cajeput in, 286; new observations on the nature and treatment of gout, ii. 376; on the remote causes of it, 377; bubo produced in the groin by it, ibid.; its effects on the viscera, ibid.; on the alimentary canal, ibid.; on the glands, ibid.; on the skin, 378; symptoms of its approach, ibid.; efficacy of bleeding in, ibid.; remedies where a feeble morbid action exists in the vessels, 379; preventives, ibid.; analysis of a gouty concretion, iii. 174; on the nature and cure of gout, and its specific causes, v. 289; on the composition of the gout Portland powder, and its dangerous effects, 417; that remedy known to the ancients, 419; Dr. Cullen vindicated on that subject, 421; use of oils in frictions, 490; benefit of the alkalies in, 562; benefit of hepatic gas in mercurial gout, vi. 92; vapours of hepatic gas efficacious in, ibid.; softness of the bones complicated with, 191; benefit of acetic ether in, 273; external use of naphtha vitrioli efficacious in, 378; on the injurious treatment of, 455; relieved by applications of aquæ ammonia acetate, 456; utility of cold water in, 457; cases of the success of that method, 458, 459; benefit of the air-pump vapour-bath, with cases, viii. 57, 58; on the nature and cure of, 349; advice of Dr. Pitcairn to gouty patients, 353; on the practice of Brown, ibid.; bad effects of the Portland powder, ibid.; advice of Dr. Darwin, 355; Dr. Cadogan's rules, ibid.; salutary effects of topical application of cold water evinced in several cases, ix. 116, 125; additional proof of the virtue of that mode of treatment, 205; account of a new vegetable remedy for, 286; beneficial effects of reduced temperature in, x. 255; cases in conformation of that practice, 256; farther evidence on the subject, 357; that treatment defended, 377; correction of an error in regard to the death of Dr. Gregory, 531; reply to Dr. Kinglake on his refrigerating system, xi. 150; that practice neither new nor safe, 151; authors enumerated who have treated the subject, 152; practice of Sydenham vindicated against the cold water plan, 154; case of the Abbé Mann, who was cured by the use of hemlock and aconite, 218; additional testimony in favour of cold applications, 346; said to be a general disease, 350; necessity of regimen in it, 351; induced by eating cucumbers and grapes, ibid.; efficacy of vermica in it, 371; reduced temperature farther asserted as the most effectual remedy, 521; sea-water recommended, 523; on the inflammation of gout, xii. 62; utility of diminishing temperature in it, 63; a general and not local disease, 64; use of ipecacuanha in it, 65; the air-pump vapour-bath recommended, ibid.; effects of ginger in it, 66; origin of the air-pump bath, 67; alleged benefit of marsh trefoil in it, 128; the opprobrium of medical science and the dread of physicians, 141; cold water in it used by the ancients, ibid.; benefit of Peruvian bark, 142; cases of the efficacy of that remedy, 143; reflections on the powers of that medicine and the nature of gout, 149; the practice of the ancients was to restore the tone of the viscera, 150; Sydenham recommended bark, ibid.; as also did Gotfred Held, 151; description of the symptoms, 152; reasons in favour of that remedy, 154; purgatives prescribed by Boerhaave, 155; virtue of Epsom salts in it, 156; extract of bark used by Storck, ibid.; opinion of Dr. George Fordyce in favour of the same, 157; benefit of guaiacum in allaying the paroxysm, ibid.; necessity of attending to diet, 158; benefit of cold bathing, 159; use of the topical application of leeches, ibid.; opium condemned, 160; account of a new remedy, 180; animadversions on a new theory of, 216; fatal effects of the cold water plan of treatment, 237; not a local disease, 238; reply to Dr. Kinglake on the refrigerant plan, 375; correspondence occasioned by the fatal case at Uxbridge, 377; opinion of Dr. Garratt on, 379; caution recommended in the use of cold water, 500; said to be a disease of the membrane covering the joints, ibid.; observations on the disorder when it attacks the stomach, 501; on the fatal case of Mr. Baker, who fell a victim to the cold treatment, 502; another fatal case, 503; review of a Dissertation on the Gout, by Dr. Kinglake, 561; the disease said to be merely local, 562; on the propagation of it to other parts, ibid.; account of the symptoms, 562; its duration, 563; cold water the natural remedy, ibid.; the gouty ailment to be obviated, 564; successful application of cold water in a case of gout, xiii. 141; remarks on the case of Mr. Baker, 144; farther proof of the danger of the refrigerant plan, 229; remarks on the case, 231; the cooling treatment not to be extended to the seat of the vital organs, 232; two cases that terminated fatally by the use of cold water, 274; additional evidence of the virtue of bark, 349; on the rashness of using cold water indiscriminately, 355; remarks on a case confirmatory of the refrigerant plan, 357; necessity of subduing the local disease, 358; on destroying the intercourse between the affected parts and the viscera, 359; method of practising the cooling treatment by immersion or affusion, 360; a disease of excessive temperature and local origin, ibid.; difference between gout and morbus crucians, 369; efficacy of the humulus lupulus in gout, 434; the refrigerant plan defended, with observations on the alleged fatal cases, 437; that plan not always to be trusted, 438; remarks on cases, 439; reply to Mr. Edlin's two instances of failure, by Dr. Kinglake, 460; the application of cold water in gout a very old remedy, 464; Dr. Kinglake in reply to Dr. Scott, 512; his re-assertion of the benefit of cold water in gout, 513; the practice of Sydenham justified, 561; on a case where the warm treatment proved fatal, xiv. 72; another instance of the benefit of the cold application, 120; benefit of aristolochia in, 171; announcement of cases in favour of the refrigerant plan of treatment, 192; reply of Dr. Kinglake to Mr. Mantel, on his theory, 215; attempt to prove that the disease is ligamentinous and tendonous, 216; salutary cautions respecting the gout, in which the doctrines of Dr. Kinglake are refuted, by John Hunt, 260; reply of Mr. Mantal to Dr. Kinglake, on his theory, 455; proofs that it originates in the inflammation of the membranes which cover the joints, 497; torpor the cause of inflammation, 498; on the metastasis of gout, 500; indicates a broken-up constitution, 502; on the cases brought forward in behalf of the cooling treatment, 503; efficacy of distilled water in, xv. 77; existence of morbific matter in, 78; observations on the nature and cure of gout, by James Parkinson, 175; an hereditary disease, ibid.; different forms of the disorder, ibid; the regular and irregular gout described, 176; saline acrimony in the blood a proximate cause of gout, ibid.; remote causes, 177; on the frequent occurrence of gout and urinary calculi in the same persons, ibid; difference between gout and rheumatism, ibid.; on particular affections of the joints, 178; on the tumours produced by it, 180; benefit of leeches, ibid.; indications of cure and mode of treatment, 181; observations on retrocedent gout, and danger of the cold water regimen, ibid.; evil effects of the Portland powder, 194; benefit of the arbutus in, 262; and of the Saponaria, 263; new work, by Dr. Hamilton, on the gout, 391; benefit of strawberries in, xvi. 264; number of gouty patients in the hospital of Dublin, 510; efficacy of Malvern waters in, xvii. 83; new work on that subject announced, 200; farther observations on the use of humulus lupulus in gouty cases, 386; the common germander an ingredient in the Portland powder, 467; case of the bad effects of the cooling treatment of gout, xviii. 53; answer to that case, 153; the cooling regimen advocated by the authority of Celsus, 165; pathological view of retrocedent gout, 167; gastroydinia a consequence of it, 168; an hereditary disease, 183; most sure in cold climates, ibid.; the refrigerating system vindicated, in reply to a case of its alleged bad effects, 240; virtue of horse-radish in, 273; farther instances of the benefit of humulus lupus in, 403; case of gout in an African negro, 578; the burdock said to be serviceable in, xix. 259; seeds and leaves of the tansy beneficial in, 354; on the effects of cinchona bark in, 427; inefficacy of soda in arthritic complaints, 429; singular effects of ginger in rheumatic gout, 527; on the benefits of the refrigerating mode of treatment, 535; case of its failure, 536; remarks on that case, xx. 65; the cold treatment defended, 66; on topical bleeding in, 67; success of the refrigerant plan, 69; often of hepatic origin, 342; cases of it in negroes, 473; case of gout and hydrothorax, xxi. 114; an hereditary disease, 285; observations on gouty concretions, xxii. 155; efficacy of gum guaiacum in arthritic affections, 361; said to be relieved by the wearing of a loadstone, xxiii. 174; associated with diseased liver, 514; efficacy of the lucca Americana, or poke-root, in, 527; account of a remarkable quack, who acquired a fortune by pretending to cure the gout, xxiv. 351; examination of the Eau Medicinale d'Husson, 352; the basis of it ascertained to be tobacco, 353; another quack-remedy devised by Monsieur Pradier, called Topique Antiarthritique, 430; infusion of saffron, lime-water, and gum guttæ, 431; on the alleged dangerous effects of the Eau Medicinale d'Husson, 474; how it came to be employed by medical men, 475; its utility in some cases maintained, ibid.; on the violence and uncertainty of the medicine, 476; case of the success of the Eau Medicinale, xxv. 114; additional cases relating to the use of the humulus lupulus in gouty and rheumatic affections, 169; on the supposed virtues of the Eau Medicinale, 184, 185; case of the fatal effects produced by that medicine, 207; sarina employed successfully in chronic gout, 444; origin of the Eau Medicinale as a remedy in gout, xxvi. 23; on the accumulation of caloric in that disorder, 96; on the dangerous effects of the refrigerating plan of treatment, 97; case of apparent paralysis of the lower extremities removed by a fit of the gout and hepatitis, 153; the white hellebore supposed to be the basis of Eau Medicinale, 185; enquiry into the treatment of gout, 189; on the use of purgatives, 190; the doctrine of Sydenham on the subject, ibid.; use of scammony in paroxysms of gout, 191; the electuarium caryo costinum recommended by Dr. Willis, ibid.; emetics serviceable as prophylactic, 192; the elaterium alleged to be the principle of the Eau Medicinale, 193; the effects of that plant in gout observed by Lister, 194; cases in which it was tried with success, 194; power of diarrhœa in carrying off a fit of gout, 196; this complaint symptomatic of the affection of the bowels, 198; gout produced by the treatment of erysipelas with camphor, ibid.; gout considered according to the humoral pathology, 199; efficacy of blisters in it, 200; volatile tincture of guaiacum recommended in, ibid.; on the theory of Cullen, 201; caution to be used in regard to boasted specifics, 202; experiments proving that hellebore is the principle of the Eau Medicinale, 224; formula of using that plant, 227; case of the effects of diarrhœa in carrying off a fit of the gout, 344; case where cold affusion in gout had nearly proved fatal, 369; on the practice of Sydenham, 380; the method of cure prescribed by Dr. Tavares, similar to that of the Eau Medicinale, 381; on the benefit of cathartics in the disorder, 382; review of a Treatise on Gout, by John Ring, 478; bleeding recommended by the ancients, 479; also by Mayerne and Willis, 480; indecision of Sydenham in his mode of treatment, ibid.; observations on the Eau Medicinale, ibid.; the gout to be cured like other inflammatory diseases, by the antiphlogistic plan, 481; bleeding necessary in plethoric habits, 481; great caution to be used in the employment of emetics, ibid.; cathartics generally useful, ibid.; strict attention to be paid to regimen, ibid.; utility of rest, ibid.; tonics advised, 482; cases of the violent effects of the Eau Medicinale, 499, 500; its complete failure and alarming consequences, 501; the rhododendrum chrysanthium a radical cure in some cases of gout, 508; popularity of the Eau Medicinale as a remedy in arthritic cases, xxvii. 32; an obsolete medicine revived, 38; on the physiognomy of gouty patients, 39; the Eau Medicinale loses its repute in France, xxix. 22; case of the efficacy of humulus lupulus in a severe paroxysm, 294; additional facts and observations on the same, 296; on the efficacy of sea-bathing in arthritic cases, xxx. 224; efficacy of strong purgatives in, 412; a new theory of the disorder, xxxi. 15; inflammatory lesion of the terminating vessels, 16; discrimination of chronic rheumatism from, 163; particulars of the discovery of the colchicum as a remedy in gout, equivalent to Eau Medicinale, xxxii. 77; method of making the tincture, 78; infusion of the root equally efficacious, 79; wine of white hellebore recommended in, ibid.; identity of the colchicum and Eau Medicinale proved, 164; observations of Dr. Sutton against the use of that remedy, 198; preparation of elecerium and opium recommended in gout, 199; utility of purgatives, 200; answer to the preceding, by Mr. Want, 201; on the hermodactyl of the ancients as a cure, 203; farther remarks on the identity of colchicum and Eau Medicinale, 207; paroxysm of the gout removed by colchicum, 312; emetics useful in gout, 314; Dr. Sutton on the effects of the Eau Medicinale, eleterium, and purgatives, in gout, 380; letter in reply to the preceding, 388; cases illustrating the modus operandi of colchicum in the cure of gout, 392; colchicum serviceable in rheumatic gout, 442; additional cases on the modus operandi of colchicum, 491; cases illustrative of the operation of the Eau Medicinale, 493; formula of Pradier's gout specific, 523; remarks on the nature of gout, and the action of remedies in it, xxxiii. 2; that there is no specific in nature for the cure, 3; debilitating effects of the Eau Medicinale, 4; case proving the advantages of colchicum in gout, 264; ptyalism a precursor of the gout, 456; defence of the cooling treatment, xxxiv. 6; objections to cold water in that disorder, 184; account of a fatal case in Somersetshire, 185; answer to the account of that case, and observations on it, 361; benefit of topical cold in, 366.

GRAFTING FRUIT-TREES, Chinese mode of, xxi. 86.

GRAHAM, Mr. his treatment of a case of enterodinia, xix. 524.

———, Dr. his case of obstructed aorta, xxxiii. 506.

GRANGER, Mr. his account of the impostor, Ann Moore, the fasting woman of Tutbury, xxii. 337; on the misquotations contained in Mr. Webster's observations on his account of the investigation of the case of Ann Moore, xxx. 189.

GRANT, Dr. on the influenza of 1803, x. 305.

GRANVILLE, Dr. his successful case of *herpes exedens vermiculatus*, xxiv. 162; on the efficacy of the rubia tinctorum as a remedy, xxvi. 105.

———, Dr. A. B. his case of caries of the bones of the skull arising from syphilis, xxix. 177.

GRAPENGIESSER, Dr. his observations and experiments on employing galvanism in the cure of certain diseases, viii. 250, 315; his experiments on galvanism in the cure of various diseases, ix. 131.

GRAPES, French method of preserving, xxii. 440.

GRASSES, description of the cotyledon and other parts of the seeds of, xxix. 510.

GRASSMAYER, Dr. his employment of the extract of belladona previously to his operating for the cataract, iii. 367.

GRAVEL, observations on it, and on urinary calculi, vi. 527; vii. 60; an infusion of *allium ursinum* in brandy, recommended in, xv. 65; a jam made of blackberries useful in, xvi. 263; observations on the, with a case, xxiv. 267. —*See* Calculi, Calculous Affections, and Lithontriptics.

GRAVES, Dr. his Conspectus of the London, Edinburgh, and Dublin Pharmacopœias, xxiv. 340.

GREASE of the horse's hoof, the vaccine disease ascribed to the, xvi. 122.

——— in the Arabian horses, observations on the, xvi. 320.

GREEKS, modern, their curious practice of midwifery, vi. 151.

GREEN, Mr. on a fatal case of hydrocele, xviii. 531; his case and successful treatment of a lacerated wound which penetrated the thorax, xxvii. 464.

GREENHOW, Mr. his case of caries of the os frontis, xii. 393.

GREGORY, Dr. his anecdote in proof of the resemblance between children and parents, xxi. 234; strictures on his theory relative to the proximate causes of fevers, 450; analysis of his Conspectus Medicinæ, xxiv. 279.

GREN, one of the authors of the improved phlogistic system of chemistry, i. 67; his doctrine of light and heat, ibid.; considered the combustion of phosphorus in the open air as the best eudiometer, ii. 389; notice of a translation of his principles of modern chemistry systematically arranged, iii. 378.

GRENADA, evidences of the infectious nature of the malignant epidemy which prevailed in, during the years 1794, 5, and 6, xxi. 241; remarkable coincidence of diseases in this island in 1783, xxiii. 166.

GREW, Dr. biographical sketch of, xxviii. 319.

GRIFFITH, Mr. on the influenza of 1803, x. 101.

GRIFFITHS, Mr. of Cardiff, on the influenza of 1803, x. 197.

———, Mr. of London, his two cases of natural small-pox after vaccination, xiv. 5.

GRIMSHAW, Rev. Mr. in vindication of the moral character of Ann Foulkes, with the result of his inquiry, xxxi. 19; Mr. Pulley's reply to the above, and to Dr. Yeats, 197.

GRIMSTONE, Mr. his successful treatment of a case of tetanus, xxv. 359.

GRIMSTONE, Mr. E. on the treatment of tetanus, xxxiv. 427.

GRIPE, the Paris influenza of 1803 so called, report on the, ix. 419.

GRIVEL, M. his account of a fœtus found in the abdomen of a woman eighty-three years of age, xv. 276.

GROIN, a fatal case of abscess in the, v. 219.

GROS CAILLOU, near Paris, experiments made at, for dis-infecting the wards of hospitals, &c. by diffusing the vapours of the oxygenated *muriatic* acid, xi. 110.

GROSE, Mr. on the medical use of yeast, iii. 105; on vaccine inoculation, 294; on the use of yeast and cold affusion in typhus fever, iv. 401; on the efficacy of yeast in typhus fever, 539; on the safety and beneficial effects of yeast in the cure of typhus fever, v. 270; on cow-pox, with nine cases of inoculation, 452; on hæmorrhagia petechialis, with three cases, vi. 233.

GROSVENOR, Honourable Robert, his doubtful case of small-pox after vaccination, xxvi. 82.

GROUND-IVY, medical properties of, xvii. 562.

GROUND-PINE, an ingredient in the Portland or gout powders, xvii. 467.

GROVE, Mr. his successful treatment of two cases of suspended animation, xx. 235.

GUAIACUM, gum, observations on, xxii. 360.

GUERIN, M. his new instrument for performing the operation of lithotomy, described, viii. 386.

GUEUDEVILLE, Dr. his chemical and medical experiments on the diabetes sugar, ix. 12; on the physical properties of diabetic urine, xi. 338.

GUILLOTIN, M. his letter on the subject of vaccine inoculation, v. 356.

GUINEA, particulars concerning the interior of, xxi. 73.

GUINEA-PIG, a new fact relative to the natural history of, xxxii. 61.

GUINEA-WORM, remarks on the, xvi. 79; history of the, and on the method of cure employed by the Hindoos, 177; observations on the, and on the scorpions of India, with the appropriate remedies, xvii. 167; observations on the, xxxiv. 57.

GUINOT, on the effects of the carbonate of potass, or salt of tartar, in puerperal fever, iii. 80, 165, 264, 363.

GUM, one of those primary elements of vegetables which can be separately exhibited as objects of sense, without undergoing any change of their native or inherent qualities, i. 75; virtues of that of the wild cherry-tree, xvi. 166.

GUM ELASTIC, its intimate combination with the matters of resin, albumen, and fat, i. 77; on the mode of dissolving, xvii. 454; a tube of, advantageously employed in a case of dysphagia, as a passage to the stomach, xix. 248; process for dissolving, xxvi. 383.

—— KINS, experiments on the substance vulgarly so called, xi. 276.

——, liquid, from Botany-Bay, analysis of the substance so called, xxix. 474.

—— RESINS, analysis of, xxv. 320.

GUMS, historical account of the, xxi. 475; xxii. 33, 358.

GUN-SHOT WOUNDS, method of treating them in the 15th century, ii. 55; Hieronymus Bramschwrig treated them as poisonous, 55, 56; improved treatment of them by Ambrose Parè, 57; treatise of Botelli on the subject, ibid.; observations on the treatment of them, 442; on the turning course of balls in them, and the effects, 444; cases of wounds in the lungs and abdominal viscera followed by complete recovery, 445; remarkable case of the lungs perforated, ibid.; farther observations, with cases, iii. 34; extraordinary treatment of them in the Prussian service, 37; bad practice among the Russians, 39; directions for the treatment of, 141; poultices recommended in, 142; on the turning course of bullets, 144; economy of nature in, 150; case of a bad one in the thorax successfully treated, 353; on the forceps commonly used for the extraction of balls, ix. 2; description of a new forceps for that purpose, 3; remarkable case of one in the face, 165; general observations on their treatment at sea, xi. 388; bleeding seldom necessary, 391; on amputation, 393; on wounds of the thorax, 394; on those of the joints, 395; on that of Sir Ralph Abercrombie, and the treatment, xii. 173, 243; review of Mr. Chevalier's treatise on, 174; farther observations on the case of Sir Ralph Abercrombie, 421, 423; method of extracting balls, 547; case of compound fracture of the humerus, xiv. 267; description, with a plate, of that which killed Lord Nelson, xv. 73; benefit of nitre in a case of, 97; description of a new instrument for extracting balls from them, with an engraving, 455; on the use of nitre in the treatment of, 504; on the mortification attending sloughing in, 505; fatal one in the peritoneum, xvi. 515; case of amputation where the os humeris was broken by a musket-ball, xxiii. 109; case of the man who was wounded on Tower-hill, 440; amputation not necessary in gangrene of, 456; case of tetanus successfully treated after a pistol-shot in the hand, xxv. 359; case of a pistol-shot in the throat, xxvii. 265; on amputation at the shoulder-joint in cases of, 378; on their fatal termination in the navy, xxviii. 89, 93; case of tetanus from gun-shot wound successfully treated with laudanum and the warm bath, xxix. 100; abstract of a treatise on that subject, by M. Richerand, xxx. 377; those wounds characterized by the disorganization of surface, 378; Ambrose Parè the first who established a true theory of them, ibid.; on the oblique action of balls, 379; balls may have one or two outlets, ibid.; on the term of wounds, ibid.; on the livid yellow colour of them, 380; jaundice occurs suddenly from, ibid.; foreign bodies lodged in them first to be sought, ibid.; description of the best forceps, 381; singular deviation of balls, ibid.; balls in such cases not easily discovered, 382; on the necessity of enlarging the orifices of wounds, ibid.; on dilatation of wounds in parts not fleshy, 458; when dilitation is indicated, ibid.; how performed, 459; on the discovery of Parè, 460; general mode of treatment after the extraction of foreign bodies, ibid.; utility of emetics, ibid; danger of fractured bones, 461; in what cases amputation is necessary, ibid.; different opinions respecting amputation on the field of battle, 462; method of treatment where the injury does not require amputation, 463; one or two bleedings necessary in young vigorous persons, but not otherwise, 464; modification of the treatment according to the organs wounded, 465; symptoms resembling tic douloureux succeeding a wound in the arm, xxxi. 499; muriate of ammonia successfully used in gangrene of gun-shot wounds, 518; case shewing the influence of the nerves on the treating of the arteries, xxxii. 248; successful case where the lower jaw was severely wounded by a grape-shot, xxxiii. 515; case of fracture of the parietal bone, xxxiv. 52; case of tetanus from a pistol-shot, 429; observations on the treatment of, 431.

GUTHRIE, Mr. his case of the cure of hydrocephalus internus, iv. 218.

GUTTA SERENA, on, and on the utility of electricity in a case of, xiv. 481.
GUTTA SERENA, or Amaurosis, essay on, i. 24.
GUY, Mr. on the preferable mode of tapping in ascites, xx. 262; his new instrument for extracting the fœtus, xxii. 104.

